# Receptor tyrosine kinase targeted therapies in glioblastoma: a systematic review

**DOI:** 10.3389/fmed.2026.1928491

**Published:** 2026-09-17

**Authors:** Audrey Z. Fu, Oliver D. Mowforth, Renuka Chintapalli, Samuel Brown, Francesca Hardyman, Richard Mair

**Affiliations:** 1 School of Clinical Medicine, University of Cambridge, Cambridge, United Kingdom; 2 Division of Neurosurgery, Department of Clinical Neurosciences, University of Cambridge, Cambridge, United Kingdom; 3 Cancer Research UK Cambridge Institute, University of Cambridge, Cambridge, United Kingdom

**Keywords:** genomics, glioblastoma, glioma, personalised therapy, receptor tyrosine kinase, survival

## Abstract

**Introduction:**

Glioblastoma is the most common primary central nervous system malignancy and has a median survival of 14.6 months. Receptor tyrosine kinase (RTKs) signalling consitutes one of the three major pathways driving glioblastoma, implicating them as a therapeutic target. However, no individual study has yet demonstrated significant survival benefit of RTK-directed therapy. The aim of this systematic review was to synthesise the current evidence on the survival efficacy of RTK-directed therapies in human glioblastoma.

**Methods:**

Studies reporting survival outcomes for human glioblastoma patients receiving any RTK-directed therapy were included. PRISMA guidelines were followed. MEDLINE, Embase, Web of Science and Scopus were searched from inception to May 2025. Citation searching of all included studies was performed for additional eligible studies. Duplicate title/abstract screening, data extraction and risk of bias assessments were conducted.

**Results:**

A total of 135 studies were included in the review, 66.7% (90/135) of which studied recurrent glioblastoma. Studies included 9,029 patients, 51.1% (4612/9029) of which were male. A total of 47 different RTK-directed therapies were assessed. Multikinase inhibitors (40.7%, 55/135), EGFR-directed therapies (28.9%, 39/135) and VEGFR inhibitors (20%, 27/135) were the most studied therapies. An additional 6.7% (9/135) of studies investigated combined EGFR-directed therapy and VEGFR inhibition. A total of 4.4% (6/135) of studies demonstrated a statistically significant survival benefit, whilst therapies appeared beneficial to survival in an additional 11.1% (15/135) of studies. There was no consistent evidence supporting survival efficacy for any RTK-directed therapy.

**Conclusion:**

Whilst there is no strong evidence for survival benefit of any RTK-directed therapy, there are encouraging results in a small proportion of studies. Future directions include identifying and validating novel biomarkers, standardising reporting, and improving clinical trial design to produce more robust and interpretable data.

**Systematic Review Registration:**

PROSPERO CRD42022366607.

## Introduction

Glioblastoma is the most common primary central nervous system (CNS) malignancy, with an annual incidence of 3.19 per 100,000 people in the United States (1). Prognosis is extremely poor, with a five-year survival rate of 5.1% (2). The current standard of care is maximal safe surgical resection followed by adjuvant radiotherapy (RT) and temozolomide (TMZ), known as the Stupp protocol. This is associated with a median overall survival of 14.6 months (3).

There have been no advances in glioblastoma care that have improved survival or prevented progression/recurrence for decades. At recurrence, only approximately 20%–30% of patients are eligible for further surgery (4). Non-surgical interventions include palliative re-irradiation, temozolomide re-challenge and other chemotherapy regimens including lomustine in patients with *MGMT* methylation (4).

With a growing interest in precision therapies, many trials in the last decade have focused on new druggable targets in commonly mutated molecular pathways. The Cancer Genome Atlas (TCGA), a large-scale publicly available cancer genome analysis programme, elucidated three major genetic events in glioblastoma, including aberrations in the receptor tyrosine kinase (RTK) pathway (5). The RTK family is a class of transmembrane proteins which act as receptors for growth factors, including epidermal growth factor (EGF), platelet-derived growth factor (PDGF), vascular endothelial growth factor (VEGF), insulin-like growth factor (IGF) and hepatocyte growth factor (HGF) (6). Signal transduction occurs upon ligand binding to the extracellular domain of the receptor, leading to dimerisation and autophosphorylation of the intracellular domain, which then recruits adaptor proteins to trigger further downstream signalling cascades (7).

Signalling through several classes of RTK has been shown to promote tumorigenesis and progression in glioblastoma (8–10). Overexpression of epidermal growth factor receptor (EGFR) is found in approximately 50% of glioblastoma cases, of which 50% harbour an *EGFRvIII* mutation, manifest as the deletion of exons 2 through 7. This results in constitutive activation of the receptor, conferring a growth advantage to tumour cells (11, 12). Recent studies support early emergence of *EGFR* on extrachromosomal DNA (ecDNA), with variant *EGFR-*ecDNAs arising from pre-existing wild type *EGFR-*ecDNAs (13). Additionally, *EGFR*-ecDNA has been found to harbour a unique methylation pattern that promotes differentiation towards mesenchymal-like and astrocyte-like phenotypes (14). Aberrant platelet-derived growth factor receptor (PDGFR) expression, also driven by amplification in ecDNA, has also been found to play a role in the induction and transformation of malignant glial cells, and when co-amplified with *EGFR* provides redundancy for signalling through the PI3K pathway (15–17). In addition, signalling through vascular endothelial growth factor (VEGF) is a key driver of the extensive neovascularisation seen in glioblastoma (18).

The role of RTKs in tumour biology has therefore made them attractive therapeutic targets, and preclinical studies inhibiting these pathways have been encouraging (19–22). A number of approaches have been taken to target RTKs. This includes signalling inhibition by monoclonal antibodies and small molecular tyrosine kinase inhibitors (TKIs), receptor-mediated internalisation for drug delivery by antibody-drug conjugates, induction of adaptive anti-tumour immunity by vaccines and T-cell mediated cytotoxicity by chimeric antigen receptor (CAR) T-cell therapies (23–26).

Given that glioblastoma remains a cancer of significant unmet need, it is important to carefully evaluate current evidence to guide future directions. Early phase glioblastoma trials with RTK-directed therapies have often been limited by sample sizes, delivered using untargeted approaches and executed in the recurrent disease setting, with biomarkers obtained at initial surgery. Moreover, agent potency and brain penetrance has improved since initial studies were performed. Individual studies may therefore not be able to offer definitive conclusions on efficacy, especially in the context of more modern agents. The aim of this systematic review was to synthesise the current evidence of the survival efficacy of RTK-directed therapies in human glioblastoma.

## Methods

### Study design

A systematic review was prospectively registered on PROSPERO (CRD42022366607) and conducted with reference to the Preferred Reporting Items for Systematic Reviews and Meta-Analyses (PRISMA) 2020 checklists (27).

### Eligibility criteria

Inclusion criteria

- Adult ≥ 18 years
- Human study
- English language
- Supratentorial glioblastoma population
- Any RTK targeted or precision or personalised or stratified therapy
- Clinical trial (randomised or non-randomised) or observational study
- Survival outcome (overall, progression-free, disease-free)

Exclusion criteria

- Review or meta-analysis
- Case report
- Letter
- Editorial
- Opinion article
- Correction

Studies that exclusively focused on the anti-VEGF monoclonal antibody bevacizumab were excluded due to extensive prior literature reviewing this therapy (28, 29). Studies using bevacizumab in combination with another RTK-directed agent were included.

### Search strategy

The search strategy was developed and piloted using an iterative process (Supplementary Materials). No automated search filters were applied to maximise sensitivity. MEDLINE, Embase, Web of Science and Scopus were searched from inception to 11th May 2025. MEDLINE and Embase searches were performed using the Ovid platform (Ovid Technologies, New York, NY, United States). Six studies known to meet inclusion criteria were used to validate search sensitivity, with all studies captured in the search. The reference lists of all included studies were hand-searched for additional eligible studies.

### Selection process

EndNote (Clarivate, United States) was used to deduplicate search results. Title and abstract screening were completed in duplicate by two blinded reviewers (AF and SB/FH) using Rayyan (Rayyan Systems Inc., United States). A pilot of 100 records were screened by all reviewers to ensure concordance. Full-text screening was subsequently conducted in duplicate by two reviewers (AF and SB/FH).

### Data collection

Manual data extraction was completed in Excel (Microsoft, United States).

### Data items

Survival outcomes, measured as overall survival (OS) and/or progression-free survival (PFS), were sought from the data. Participant and study characteristics were also extracted.

### Risk of bias assessment

Risk of bias of included studies was assessed in duplicate using the Joanna Briggs Institute critical appraisal tools checklists (Supplementary Materials) (30).

### Synthesis methods

Meta-analysis was not feasible due to the heterogeneity in study and participant characteristics and the range of interventions studied. A qualitative synthesis was therefore conducted using a 4-point scale: survival benefit associated, no survival benefit associated, appears beneficial, appears unbeneficial (31). Only studies that reported a comparison between an intervention group and a control group with an appropriate statistical test conducted were scored as associated or not associated with a survival benefit. Where this was not applicable, a score of appears beneficial or unbeneficial was assigned with reference to established survival data following standard care.

## Results

### Study selection

A total of 6,789 records were identified by our search, of which 4,554 remained after de-duplication. A total of 135 studies met criteria for inclusion in the review (Figure 1).

**Figure 1:**
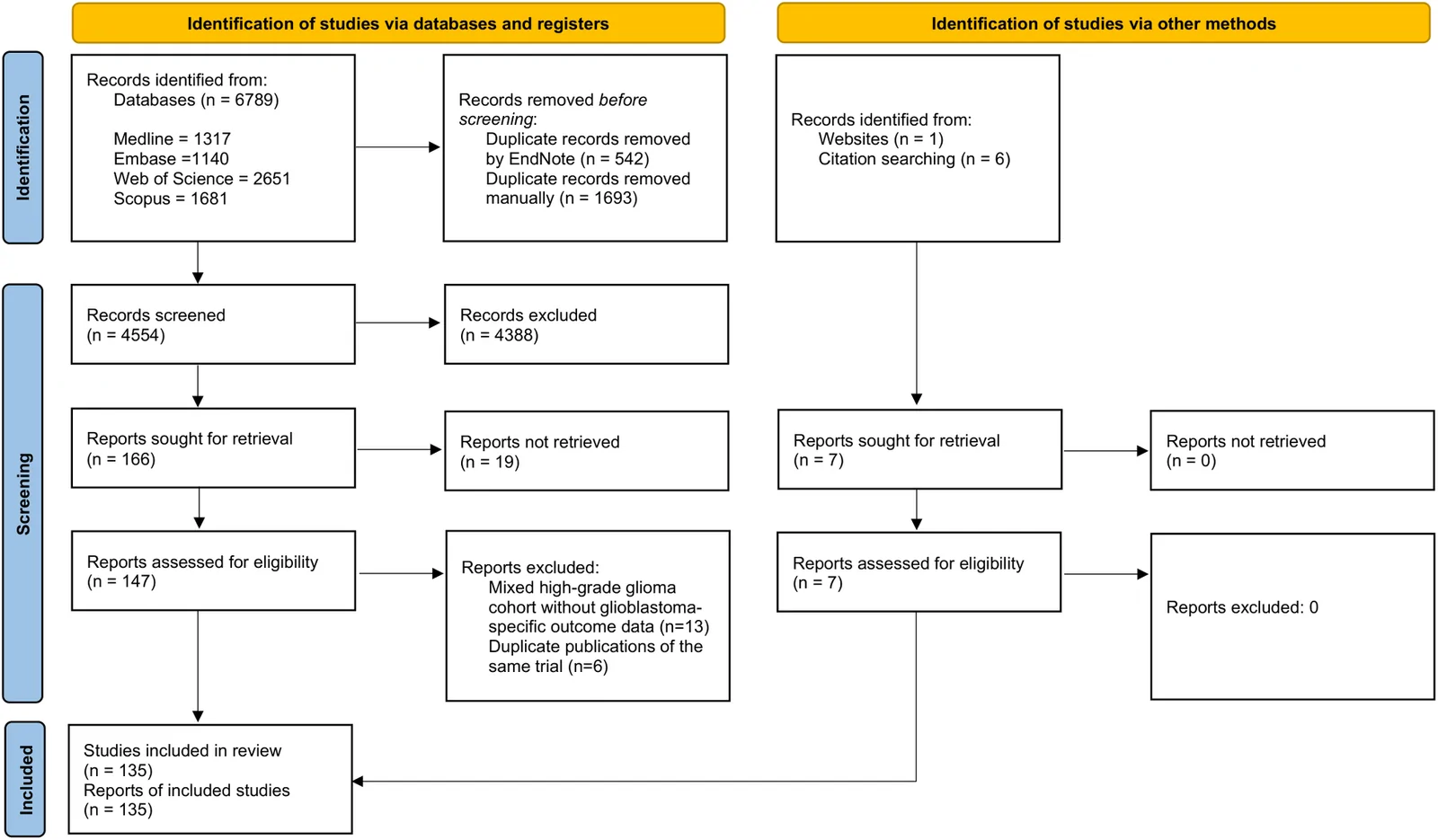
PRISMA flow diagram of study selection. After the removal of duplicate records, title/abstract screening was conducted for 4,554 records. There were 19 reports where the full text could not be retrieved and an additional 19 studies that failed to meet inclusion criteria upon full-text screening. A total of 121 full text articles and 14 conference abstracts were included in the final analysis. Citation searching of included studies was conducted and an additional six studies were included.

### Study characteristics

A total of 47 drugs targeting RTKs were studied, including combinatorial therapies. Multikinase inhibitors (40.7%, 55/135), EGFR-directed therapies (28.9%, 39/135) and VEGFR inhibitors (20%, 27/135) were the most studied therapies (Figure 2A). Amongst the EGFR-directed therapies, 71.8% (28/39) were inhibitors of EGFR signalling and 28.2% (11/39) used EGFR expression in glioblastoma to deliver drugs or immunologically target cells. Recurrent disease was more frequently studied than newly diagnosed glioblastoma with 66.7% (90/135) of studies exclusively studying patients at recurrence (Figure 2B). Of the 135 included studies, 85.2% (115/135) were interventional trials and 14.8% (20/135) were observational cohort studies. Of the interventional studies, 87.0% (100/115) were phase I, I/II or II trials, 4.3% (5/115) were phase III trials, and 8.7% (10/115) were pilot studies or multi-centre basket trials (Figure 2C). The majority of therapies studied appeared unbeneficial to survival (Figure 2D).

**Figure 2:**
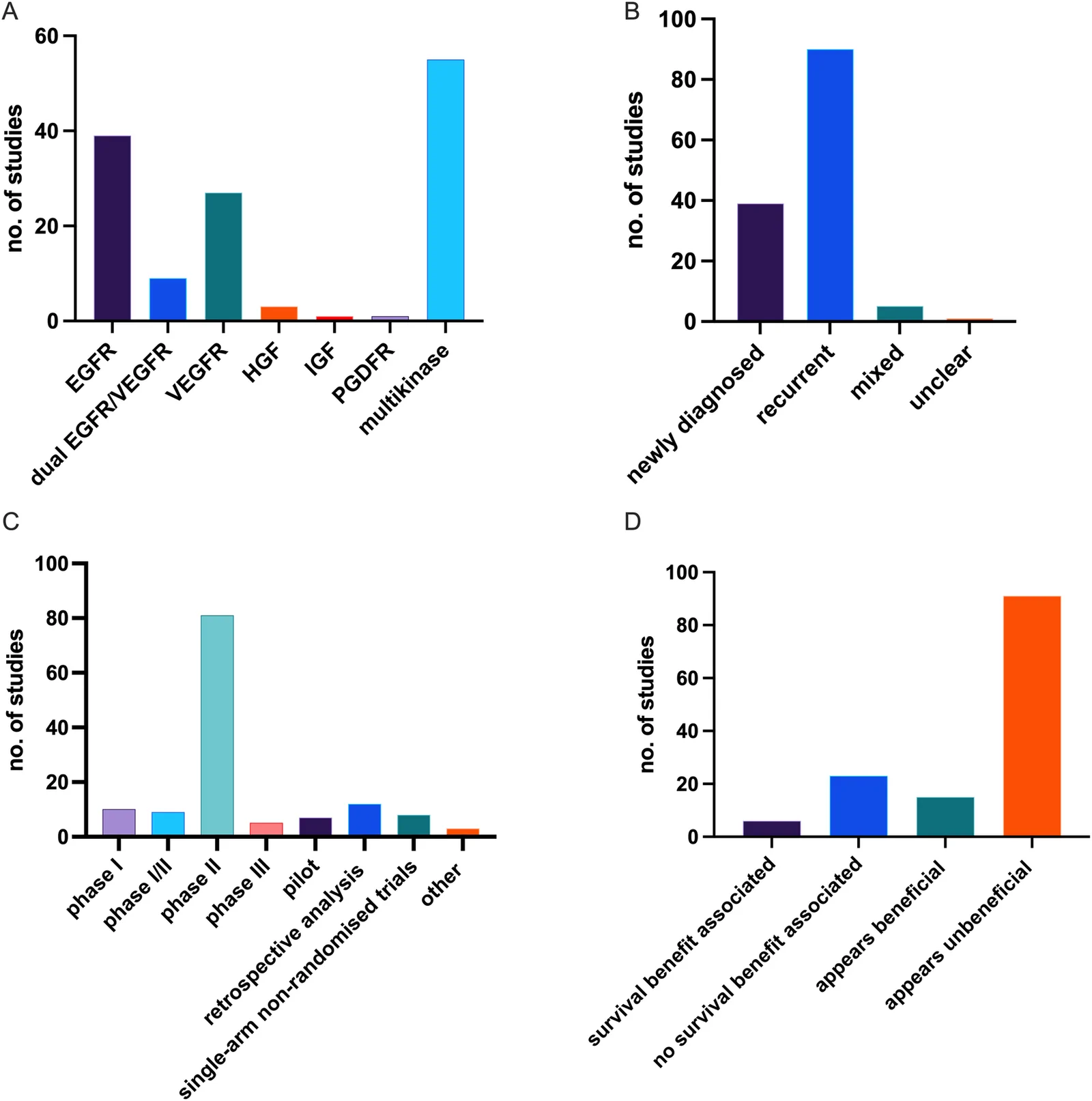
**(A)** The most studied drugs were multikinase inhibitors, comprising (40.7%, 55/135) of total studies. This was followed by EGFR (28.9%, 39/135), VEGFR (20%, 27/135) and combined EGFR/VEGFR (6.7%, 9/135) targeted drugs. **(B)** Newly diagnosed disease was studied in 28.9% (39/135) and recurrent disease in 66.7% (90/135). The included cohort was mixed in five studies and unclear in one study. **(C)** Phase II trials made up the majority of included studies (60%, 81/135). **(D)** A total of 4.4% (6/135) of studies demonstrated a survival benefit and 17.0% (23/135) studies demonstrated no survival benefit. Therapies appeared unbeneficial to survival in 67.4% (91/135) of studies and beneficial in 11.1% (15/135) of studies.

### Patient characteristics

Studies included a total of 9,029 patients, 51.1% (4612/9029) of which were male. The mean number of patients per study was 67. Prior and concurrent therapies received by patients were not consistently reported. Time from initial diagnosis to starting an RTK-directed therapy ranged from 1.9 to 26.9 months. Studies were conducted in 18 different countries (Figure 3).

**Figure 3:**
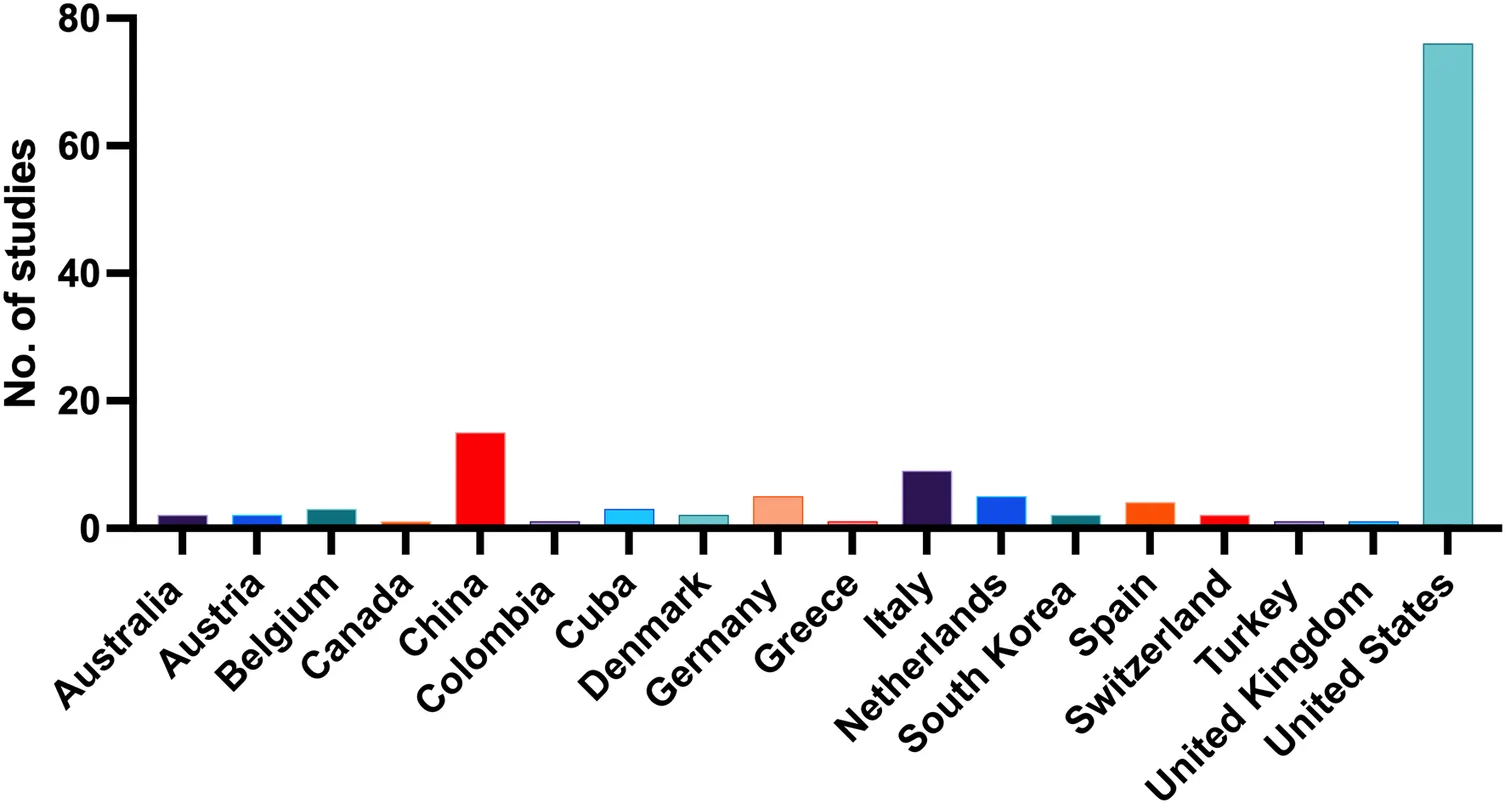
The majority 56% (76/135) of studies were conducted in the United States, followed by China with 11% (13/135) and Italy with 6.7% (9/135).

## RTK targets

### Epidermal growth factor receptor (EGFR)

Erlotinib, a first-generation small molecule tyrosine kinase inhibitor (TKI), was the most studied of these drugs. There were several studies of erlotinib for recurrent glioblastoma, as monotherapy or in combination with radiotherapy (RT) and temozolomide (TMZ) (32–37). Notably, a randomised trial of 110 patients with recurrent disease found no significant survival benefit for erlotinib monotherapy compared to either TMZ or carmustine (32). There were mixed results for newly diagnosed patients: three studies with a total of 145 patients found that erlotinib in combination with RT or TMZ appeared unbeneficial for survival, while another study of erlotinib and RT reported a median OS of 19.3 months and median PFS of 8.2 months, which were significantly longer than the historical control cohort (38–41). There was a further significant survival benefit on stratification of the 16 patients with *MGMT* promoter methylation, however *EGFR* mutation status was not analysed for either the treatment or historical control group (38).

Other first-generation EGFR TKIs studied include gefitinib (Table 1). Four non-randomised trials studied gefitinib as a monotherapy or in combination with RT or everolimus, none of which appeared beneficial to survival (42–45).

**Table 1:** Classes of EGFR-directed therapies included.

Class of EGFR-directed therapy	Name of therapy
1st generation EGFR inhibitor	Erlotinib, gefinitib
2nd generation EGFR inhibitor	Afatinib, dacomitinib
Monoclonal antibodies	Nimotuzumab, Mab-425, Sym004, Panitumumab, GC1118
Antibody-drug conjugates	Depatuximab mafodotin
Vaccine	Rindopepimut
CAR-T therapy	Anti-*EGFRvIII* CAR-T cells

Second generation EGFR TKIs studied include afatinib and dacomitinib. A randomised phase II trial of 119 patients with recurrent disease receiving afatinib found no significant difference in median PFS or OS for patients who received afatinib monotherapy or in combination with TMZ compared to TMZ monotherapy (46). Dacomitinib was studied in two non-randomised phase II trials that recruited patients with known *EGFR* amplification, with OS ranging from 6.7 months to 9.9 months (47, 48). However, neither trial found an association between clinical benefit and *EGFRvIII* mutational status.

Of the 39 studies of EGFR-directed therapy, 17 studies reported data on correlation between *EGFR* amplification and *EGFRvIII* status of included patients and survival outcomes (Figure 4). *EGFR* amplification status was determined by fluorescent *in-situ* hybridisation (FISH) while *EGFRvIII* status was determined by either reverse transcription polymerase chain reaction (RT-PCR) or immunohistochemistry. One study found a significant correlation between *EGFRvIII*-positivity and poorer PFS in the erlotinib treatment arm (*p* = 0.003) but not the control arm (*p* = 0.714), and a borderline significantly reduced PFS for *EGFR*-amplified patients for the entire study population (*p* = 0.048) (32). In contrast, one phase I/II study, in which *EGFRvIII* status was assessable in 59% of patients, demonstrated longer median PFS in the afatinib monotherapy and afatinib and TMZ treatment arms for patients with highly positive *EGFRvIII* status compared to *EGFRvIII* negative patients, with one patient reported to remain progression free at 48 months (46). However, this study demonstrated poorer median PFS for both afatinib monotherapy and afatinib and TMZ compared to TMZ (46). All other studies did not find a correlation between *EGFR* mutational status and PFS or OS.

**Figure 4:**
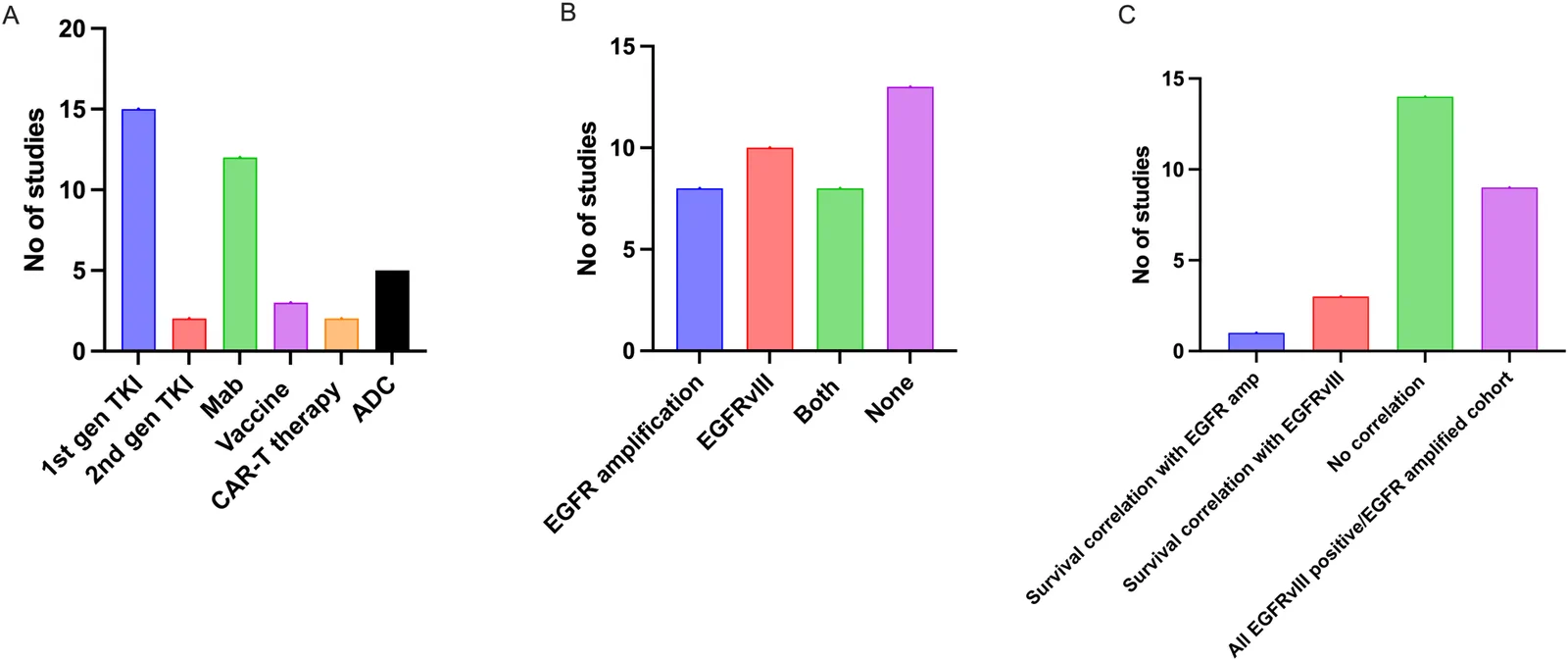
**(A)** of 39 studies of EGFR-directed therapy, 15 were of first-generation small molecule tyrosine kinase inhibitors (TKI), two of second-generation small molecule TKIs, 12 of monoclonal antibodies (Mab), five of antibody-drug conjugates (ADC), three of vaccines and two of CAR-T therapy. **(B)** In terms of target biomarkers for EGFR-directed therapy, eight studies reported *EGFR* amplification, 10 reported *EGFRvIII,* eight reported both amplification and *EGFRvIII* and 13 did not report on any biomarkers. **(C)** Of 39 studies of EGFR-directed therapy, nine studies included only *EGFR* amplified or *EGFRvIII* positive patients and did not perform biomarker survival analysis, while 17/39 studies reported on correlation between *EGFR* status and survival outcome following EGFR-directed therapy. A majority of studies (82%, 14/17) did not find any correlation between *EGFR* status and survival outcome following EGFR-directed therapy.

Monoclonal antibodies targeting EGFR included nimotuzumab, mAb-425, Sym004, panitumumab and GC1118. Nimotuzumab is an EGFR-targeting monoclonal antibody which blocks ligand binding, with a secondary mechanism of triggering tumour cell destruction by antibody-dependent cell mediated cytotoxicity. It had mixed survival results across five non-randomised studies (49–53). In addition, a phase III trial found no survival benefit for nimotuzumab, with subset analysis also demonstrating no survival benefit for EGFR expression-positive patients, defined by immunohistochemistry (54). A phase II trial of Sym004 did not report OS or PFS but reported a progression-free survival at 6 months (PFS6) of 12.5% (55). Two trials studied the adjuvant use of radioiodine labelled monoclonal antibody-425 (mAb-425): a small phase II trial found a similar median OS to that of the Stupp cohort, while a larger phase II trial found a significant prolongation of OS compared with a historical cohort in patients receiving mAb-425 monotherapy, with a further improvement in the TMZ combination therapy group (56, 57). However, the historical cohort had a median OS of 7.2 months, which is notably shorter than that of the Stupp cohort.

Panitumumab appeared unbeneficial in a prospective multicentre basket trial of 27 patients with recurrent disease with a median OS of 4.5 months and no correlation to *EGFR* amplification status (58). A phase II trial of CG1118 in 21 patients with recurrent *EGFR*-amplified glioblastoma also found that the drug appeared unbeneficial to survival with an OS of 5.7 months (59).

Several trials studied Depatuximab mafodotin (Depatux-M/ABT-414), an antibody-drug conjugate comprised of a humanised monoclonal antibody against EGFR conjugated with the anti-microtubule agent monomethyl auristatin F. Early phase non-randomised trials combining Depatux-M and TMZ demonstrated mixed results for both newly diagnosed and recurrent disease (60–62). A phase II trial found no survival benefit of Depatux-M in combination with TMZ in recurrent glioblastoma with confirmed *EGFR* amplification in the primary analysis, although long-term analysis found a significant difference in OS in favour of this treatment arm (HR 0.66, *p* = 0.017), suggesting possible clinical benefit for a subset of patients (63). Four of these studies collected data on either *EGFR* amplification or *EGFRvIII* status, with no correlation found with survival outcomes. A later phase III trial found no survival benefit of Depatux-M, RT and TMZ compared to the control arm for patients with newly diagnosed *EGFR*-amplified glioblastoma in an interim analysis and was terminated early (64). However, there was significant prolongation of the median PFS to 8 months in the interventional group compared to 6.3 months in the control group (*p* = 0.029). This was further prolonged in the *EGFRvIII*-mutant group with a median PFS of 8.3 months compared to 5.9 months in the control group (*p* = 0.002). One non-randomised trial of 11 patients with recurrent glioblastoma of Depatuximab alone (without a drug conjugate) also appeared unbeneficial to survival (65).

Studies of other therapeutics targeting *EGFRvIII* had mixed results. Several trials studied rindopepimut, a vaccine that targets *EGFRvIII* and induces an adaptive immune response, with only *EGFRvIII*-positive patients included in these trials. Early studies showed promising results, including a cohort of 18 patients achieving a median OS of 26 months, which was significantly prolonged compared to a matched historical cohort, and the ACT III single-arm trial which found a median OS of 21.8 months (66, 67). However, the ACT IV trial, a phase III RCT of 745 patients, found no survival benefit for rindopepimut and TMZ compared to placebo and TMZ in a cohort of *EGFRvIII*-positive patients, defined as greater than 10% of sampled cells staining positive for *EGFRvIII* on immunohistochemistry (68). Two other small studies with 26 patients in total found that *EGFRvIII*-directed CAR-T cell therapy appeared unbeneficial to survival (69, 70).

### Vascular endothelial growth factor receptor (VEGFR)

VEGFR TKIs studied included cediranib, axitinib, apatinib and tivozanib, in addition to the extracellular VEGFR-2 antagonist pegdinetanib.

A phase I trial of 20 patients with recurrent glioblastoma receiving cediranib and the *α*v*β*3/*α*v*β*5 integrin inhibitor cilengitide reported a median OS of 6.5 months and median PFS of 1.9 months (71). Following a phase II study of cediranib monotherapy in recurrent disease, a phase III RCT of 325 patients found no OS or PFS benefit for cediranib monotherapy or in combination with lomustine for recurrent glioblastoma compared with lomustine alone (72, 73). A later phase II trial with 158 patients of cediranib with RT and TMZ for newly diagnosed glioblastoma similarly found no significant survival benefit compared to placebo (74). In a randomised phase II trial of 70 patients in which cediranib was combined with the poly-ADP ribose polymerase (PARP) inhibitor olaparib, there was also no significant survival benefit for patients with recurrent glioblastoma compared to bevacizumab (75). Three of these trials included measurement of plasma biomarkers including circulating VEGF, sVEGFR1 and sVEGFR2; in all three trials there was no association between biomarkers level and survival outcomes (71–73).

Duerinck et al. conducted two phase II RCTs studying axitinib. The first trial of 44 patients compared axitinib monotherapy to physician's choice therapy, and the second trial of 79 patients compared axitinib in combination with lomustine to axitinib monotherapy (76, 77). Neither demonstrated a significant difference in OS, with this ranging from 17 to 29 weeks between the two arms. Another phase II trial included 54 patients receiving axitinib and the programmed death-ligand 1 (PD-L1) inhibitor avelumab using two different dosing regimens, resulting in a median OS of 18 weeks and 26 weeks (78). Apatinib, tivozanib and pegdinetanib (CT-322) were studied in non-randomised trials in which they did not appear beneficial to survival (79–84). The phase II trial of tivozanib found significant changes in MRI and blood biomarkers of angiogenesis after treatment, including plasma sVEGFR2, but no correlation with OS or PFS (81). Biomarker analysis was not reported for the axitinib, apatinib and pegdinetanib studies.

After bevacizumab, thalidomide is the second most studied anti-VEGF drug. Thalidomide and TMZ appeared beneficial in a phase II trial with a mix of 44 patients with newly diagnosed and recurrent glioblastoma with a median OS of 23.7 months (85). Another phase II trial studying the combination of thalidomide, TMZ and RT in a cohort of 67 newly diagnosed glioblastoma patients found a median OS of 16.8 months (86). However, eight other prospective trials with a total of 647 patients did not find an overall survival benefit (87–94). Two trials included biomarker analysis by measuring plasma VEGF. One study found no correlation with survival outcome, while another found higher plasma VEGF levels were negatively associated with PFS and OS, although this was not statistiscally significant (87, 94).

Other drugs in this class include aflibercept, a VEGF trap, ramucirumab, a monoclonal antibody targeting VEGFR-2, and ABT-510, a thrombospondin-1 mimetic. In a phase II trial of 42 patients with recurrent glioblastoma, aflibercept demonstrated a median PFS of 2.7 months, a median OS of 8.9 months and did not meet the PFS-6 efficacy endpoint (95). Tissue samples from original surgery were available for 69% of patients; RNA from these patients was extracted and analysed for genome-wide expression patterns. Notably, *HIF1A* expression, which increases the expression of *VEGF* in hypoxic environments, was found to be correlated with a longer time to progression (*p* = 0.00492) (95). In a trial of 40 patients with recurrent glioblastoma, ramucirumab appeared unbeneficial with a median OS of 11.4 months (96). Biomarker analysis was not reported for this study. Finally, a phase I trial of 24 newly diagnosed patients found that ABT-510 appeared unbeneficial with a median OS of 14.8 months (97). Differential gene expression analysis for patients alive after three years of enrolment and those who were not, identified that low levels of *TIE1* and *FGF1* correlated with longer survival.

### Dual VEGFR/EGFR

A number of studies assessed combinations of VEGFR inhibitors with EGFR inhibitors or EGFR-directed vaccines. One randomised phase II trial of 38 patients found that the combination of cediranib and gefitinib for recurrent glioblastoma was not associated with a significant improvement in survival, with a median OS of 7.2 months compared to 5.5 months in the control arm (98). Recruitment was terminated early due to negative results from a phase III trial of cediranib monotherapy (72).

A preliminary study found that the combination of bevacizumab and erlotinib appeared beneficial for survival in a cohort of 4 patients with *EGFRvIII* positive newly diagnosed glioblastoma with a median OS of 17.0 months (99). A later preliminary study by the same group selected 14 patients with recurrent glioblastoma with *EGFRvIII* positivity and normal *PTEN* expression to receive bevacizumab and erlotinib and reported a PFS6 of 64.3%. This was higher than that reported in the EORTC26101 trial, however OS was not reported (100). Another phase II trial studying the use of bevacizumab and erlotinib in 25 recurrent glioblastoma patients found a median OS of 10.3 months, and a PFS6 of 29.6% (101). A phase II trial of 59 newly diagnosed patients receiving bevacizumab and erlotinib found that median PFS was increased at 13.5 months compared to 8.6 months in the historical control (*p* = 0.03), however there was no significant prolongation of OS (*p* = 0.33) (102). A retrospective analysis studied 15 patients with *EGFRvIII* positive recurrent glioblastoma who had received a combination of bevacizumab and osimertinib, a third-generation *EGFR* TKI with greater penetration of the blood-brain barrier (BBB) compared to earlier generations (103). This combination therapy appeared unbeneficial to survival with a median OS of 9 months and median PFS of 5.1 months (103).

Whilst monotherapy studies of the EGFR-targeting monoclonal antibody nimotuzumab discussed above generally did not demonstrate a survival benefit, it appeared beneficial in a retrospective analysis of 18 newly diagnosed patients who received it alongside bevacizumab, RT and TMZ, with a median OS of 28 months and median PFS of 18 months (104). Cetuximab (EGFR-targeting monoclonal antibody which blocks ligand binding with a secondary mechanism of triggering tumour cell destruction by antibody-dependent cell mediated cytotoxicity), bevacizumab and irinotecan were studied in a phase II non-randomised study of 43 patients with newly diagnosed glioblastoma and did not appear beneficial for OS and PFS (105).

A phase II RCT (reACT trial) studying the combination of rindopepimut with bevacizumab included 73 patients with *EGFRvIII* positive recurrent glioblastoma and found a significant improvement in OS (106).

### Platelet derived growth factor receptor (PDGFR)

Tovetumab (MEDI-575) and olaratumab are monoclonal antibodies against PDGFR*α*. A phase II trial of tovetumab in 56 patients with recurrent glioblastoma found a median OS of 9.7 months (107). Similarly, olaratumab was associated with a median OS of 7.9 months in a trial of 40 recurrent glioblastoma patients (96).

### Insulin-like growth factor receptor (IGFR)

A randomised phase Ib trial of IGV-001, an autologous cell vaccine produced by incubating glioblastoma cells with antisense oligonucleotide against IGF-1R, in 68 newly diagnosed glioblastoma patients found that it appeared beneficial to survival. The median OS of all patients who received the vaccine was 17.3 months, which increased to 22.3 months and approached statistical significance compared to historical cohorts receiving standard care when stratifying for Stupp-eligible patients (16.2 months, *p* = 0.08). A further survival benefit was found in the cohort randomised to the highest vaccine exposure compared with historical cohorts, with a median OS of 38.2 months (*p* = 0.044) (108).

### Hepatocyte growth factor receptor (HGFR)

Monoclonal antibodies targeting this pathway include rilotumumab and onartuzumab, which both aim to abrogate HGF/MET signalling. There were two phase II non-randomised trial of rilotumumab for recurrent disease. One study with 36 patients observed a median OS of 11.2 months, while the second study with 60 patients reported a median OS of 6.5 months for a higher dose and 5.4 for a lower dose (109, 110). Onartuzumab was not associated with survival benefit compared to the control arm for either PFS or OS in a phase II randomised trial of 129 patients (111).

### Multikinase inhibitors

Multikinase inhibitors studied include regorafenib, anlotinib, sorafenib, imatinib, sunitinib, vandetanib and lenvatinib.

The REGOMA phase II randomised trial of 119 patients found that regorafenib monotherapy for recurrent glioblastoma was associated with a survival benefit with a median OS of 7.4 months compared to 5.6 months for the lomustine control arm (HR = 0.5, *p* = 0.0009) (112). However, the OS of the control arm was notably shorter than previously reported data. There were four subsequent observational studies of regorafenib in recurrent glioblastoma with encouraging results. A retrospective analysis including 54 patients receiving regorafenib as second-line therapy after progressing on the Stupp protocol demonstrated a median OS of 10.2 months (113). The REGOMA-OSS study, a larger multicentre prospective observational study with 190 patients also found a median OS similar to that of the REGOMA trial of 7.9 months (114). A retrospective analysis of 65 patients who had progressed on second-line bevacizumab and were treated with third-line regorafenib found a slightly reduced median OS of 4.1 months, while another retrospective analysis with 41 patients receiving regorafenib as second or third-line therapy at recurrence reported a median OS of 22 months (115, 116).

Anlotinib appeared beneficial in combination with TMZ and RT in a phase II trial of newly diagnosed patients, with a median OS of 17.3 months (117). Furthermore, a retrospective analysis of 17 patients with recurrent disease treated with anlotinib reported a similar OS of 17 months (118). However, it was studied in six other trials of recurrent disease with a total of 122 patients where it did not appear beneficial to PFS or OS (119–124).

Sorafenib added to RT and TMZ for 47 patients with newly diagnosed glioblastoma did not appear beneficial to OS in a pilot study (125). A retrospective study of 24 recurrent glioblastoma patients who received sorafenib monotherapy reported a median OS of 6 months (126). Other regimens were tested in non-randomised trials for recurrent disease, including a phase II trial of 43 patients receiving sorafenib and TMZ, a phase II trial of 54 patients receiving a combination of sorafenib and bevacizumab, and a phase I/II trial of 115 patients receiving sorafenib and temsirolimus, all of which appeared to find no benefit to OS and PFS (127–129). In addition, two phase II trials of erlotinib and sorafenib studying a total of 75 recurrent glioblastoma patients found a similar median OS of approximately 5 months, with one study also finding no prolongation of OS compared to a matched historical cohort (130, 131).

A phase III randomised trial compared the use of imatinib with hydroxyurea monotherapy in 240 patients with recurrent glioblastoma who had progressed on standard dose TMZ and found no benefit in PFS and OS (132). All other imatinib trials were non-randomised and found that it did not appear beneficial to survival (133–140). Similarly, sunitinib appeared unbeneficial to survival in five phase II single-arm trials with 168 patients in total, and one phase II/III trial of 55 patients with recurrent glioblastoma identified no survival benefit for sunitinib compared to lomustine (141–146).

A number of other multikinase inhibitors were assessed by a smaller number of studies. Notably, crizotinib showed encouraging results in a phase Ib randomised trial of 38 patients with newly diagnosed glioblastoma, with a median OS of 22.6 months. In addition, there was a significantly prolonged survival for the *MGMT* methylated subgroup with a median OS of 31.4 months compared to 18.6 months for the unmethylated subgroup (*p* = 0.001) (147). Subgroup analysis for patients with positive c-MET alterations was not conducted due to limited number of patients.

A phase II trial in 15 patients with recurrent glioblastoma found that ponatinib appeared unbeneficial to survival with a median OS of 3.22 months (148). The study noted that no patients in their cohort with molecular testing available had the *FGFR-TACC* chromosomal translocation which has been associated with sensitivity to FGFR inhibitors in preclinical trials (149).

A randomised trial of lenvatinib in 80 patients with recurrent disease found no significant survival benefit compared to bevacizumab (150). A single-arm phase II trial of lenvatinib with pembrolizumab with 101 patients also appeared unbeneficial with a median OS of 8.6 months (151). A phase I/II trial of the multikinase inhibitor pazopanib with the EGFR inhibitor lapatinib reported a median PFS of 8–9 weeks and was terminated due to lack of efficacy (152). A phase II trial of pazopanib alone for recurrent glioblastoma reported a median OS of 8 months and median PFS of 2.76 months (153).

A phase II trial with 115 patients with newly diagnosed glioblastoma found that vandetanib in combination with TMZ and RT had no associated survival benefit compared with RT and TMZ (154). Similarly, a phase II trial of 62 newly diagnosed patients receiving vandetanib found no survival benefit compared with carboplatin (155). Other trials of vandetanib had similar results (156, 157).

A randomised phase II trial of 204 newly diagnosed patients who received dasatinib, TMZ and RT found no survival benefit compared to the control arm of TMZ, RT and placebo, with a median OS of 15.6 and 19.3 months respectively (158). Similarly, two non-randomised phase II trials and one retrospective study including a total of 80 recurrent glioblastoma patients found that dasatinib did not appear beneficial to survival (159–161).

There were also non-randomised trials of a number of other multikinase inhibitors including nintedanib, dovitinib, tandutinib, and cabozantinib. These appeared unbeneficial to survival for recurrent glioblastoma (162–167).

## Discussion

### Summary of main findings

The aim of this review was to synthesise the current evidence on the survival efficacy of RTK-directed therapies in glioblastoma. We found that most studies reported interventions that appeared unbeneficial to survival.

There appears to be promise with more modern multi-kinase inhibitors, including anlotinib, which in conjunction with radiotherapy has good blood-brain barrier permeability in preclinical studies with clinical efficacy in treating brain metastases from lung cancer, and regorafenib, which has high potency despite relatively poor brain penetrance in malignant glioma patients (168–171). The personalised autologous immunotherapy IGV-001, has also produced promising survival outcomes in early phase trials and will be further explored in larger trials (108, 172).

RTK therapy is highly targeted, and with few existing prognostic or predictive factors for therapy response, and diverse mechanisms of action, it is important to investigate which patients are most likely to benefit from a particular RTK-targeted therapy. Despite preclinical and clinical data suggesting increased susceptibility and response to EGFR-targeted drugs in *EGFR*-amplified or *EGFRvIII*-positive glioblastoma, overall there appears to be no strong evidence to suggest an increased survival benefit for these patients (173–175). Many EGFR-targeted therapy trials performed subgroup analyses based on mutational status and some trials only included patients with a mutation; most did not find a correlation between *EGFR* amplification or mutational status and survival outcome (27, 61). However, whether subgroup analyses were sufficiently powered was often unclear.

For trials selecting patients based on *EGFR* status, there is uncertainty whether patients with recurrent disease still had the target mutation at the time of treatment as trials were typically stratified based on tissue samples obtained at time of diagnosis. During the time elapsed from the biopsy, genetic instability, clonal evolution, and selection pressures from therapies may lead to altered tumour mutational status. One study on spatiotemporal evolution in glioblastoma found that distant recurrences share a third of mutations with the primary tumour, often with different driver mutations (176). Reports of the stability of *EGFRvIII* status at recurrence range from 50% to 84% (177, 178). There is therefore a risk that the mutational profile at biopsy does not reflect the key mutations at the time of treatment, such that efficacy analyses as a function of mutational status may not be interpreted accurately. The invasiveness of surgical biopsy makes this unattractive, strengthening rationale for development of longitudinal liquid-biopsy based biomarkers (179).

There are various hypotheses on the lack of efficacy of RTK-directed therapies. Firstly, there may be variable delivery across the blood-brain barrier. There is evidence from resected glioma samples from patients receiving RTK therapies that early generation EGFR inhibitors such as erlotinib and gefitinib do not significantly accumulate or inhibit EGFR in tumour tissue (180). Pre-clinical studies in animal models have demonstrated similarly low permeability of the blood-brain barrier for several other drugs including sorafenib, imatinib and cediranib (125, 181, 182). Notably, disruption of the BBB by tumour growth and radiotherapy may result in drug penetrance that is greater than predicted preclinically (183). However, while the integrity of the BBB is similarly reduced in regions of glioblastoma, there is still a significant tumour burden with an intact BBB that require drugs with greater permeability to be adequately treated (184).

Newer generations of EGFR inhibitors have demonstrated improved blood-brain barrier penetrance and clinical efficacy in treating brain metastases (185–187). BBB penetrance of the monoclonal antibodies studied is largely unclear (53). Notably, a study of patient derived xenografts implanted in animal models found that Depatux-M demonstrated significant survival benefit in heterotopic flank tumours, but variable benefit in orthotopic intracranial tumours, with heterogenous distribution of the drug in tumour tissue (188).

For the TKIs, aside from BBB penetrance, the ability to inhibit RTK signalling is also determined by potency. Early generation EGFR TKIs generally have high potency for wild type EGFR (189, 190). Osimertinib, a third generation EGFR inhibitor with increased BBB penetrance, is specific for the T790M mutation (IC50 = 6 nM) but has lower potency for wild type EGFR (IC50 = 938 nM) (191). Further investigation of drugs with higher potency against wild type EGFR in validated *EGFR*-amplified cohorts may be useful, alongside development of potent drugs with activity against both wild type EGFR and *EGFRvIII* (192). Cediranib and axitnib, the two most frequently studied drugs against VEGFR, display high potency (IC50 of <1 nM), while multikinase inhibitors were more variable (IC50 ranging from 4.2–90 nM) (21, 170, 193–195).

One hypothesis requiring further investigation is that resistance to TKIs occurs due to intratumoural heterogeneity. Glioblastoma consists of distinct subclones with different molecular profiles, each subpopulation possibly driven by distinct RTK mutations, such that single receptor targeting is ineffective (17, 196, 197). There is also significant spatial heterogeneity, with a hypoxic core, immune cells and glial cells interacting within the tumour microenvironment, factors which may contribute to differing treatment response (198–200).

Redundancy amongst RTK pathways has been characterised in preclinical models. For example, in cultured cells with *EGFR* and *PDGFR* amplification, simultaneous inhibition of both EGFR and PDGFR has been shown to be necessary to abrogate PI3 kinase pathway activity (17). While a phase III trial and a large phase II trial combined the multikinase inhibitor imatinib with hydroxyurea, which was suggested to induce the loss of amplified genes, the presence of co-occurring mutations that may contribute to RTK signalling redundancy and therapeutic failure have not been assessed (123, 130). A non-randomised phase II trial of imatinib also found no correlation between survival outcomes and *EGFR* status on immunohistochemistry (130).

Signalling pathways downstream of RTKs play key roles in tumour cell survival and proliferation, and activation of these pathways may also contribute to resistance to RTK inhibitors. For example, aberrant MEK1/2 signalling has been implicated downstream of oncogenic alterations, including activating mutations in BRAF and *EGFR* overexpression associated with an autocrine signalling loop (201). Trametinib, a MEK1/2 inhibitor, has been studied in combination with dabrafenib, a BRAF inhibitor, in glioblastoma patients with the BRAF V600E mutation (201). This combination therapy has been approved for use in paediatric glioma (202). Inhibiting both upstream RTKs and downstream intracellular kinases concomitantly may have promise in overcoming therpeutic resistance.

### Limitations of included trials

Many included trials did not include a suitable control arm for comparison. Of the trials with a control arm, some employed historical cohorts in which patient characteristics were not matched. Matching controls by age, sex, prior treatment and molecular biomarkers of interest is important when making comparisons seeking to interpret the efficacy of molecularly targeted therapies. For studies which included multiple subgroup biomarker analyses, it was not always clear whether appropriate methods were used to account for multiple testing.

Moreover, most trials were non-randomised studies that did not include a matched control cohort. We therefore relied on survival data from the Stupp cohort for newly diagnosed glioblastoma receiving the standard of care as a benchmark of comparison for survival (3). However, with no established standard of care for recurrent glioblastoma, there is a range of reported survival data from different interventions, with larger trials reporting 7.3 months in the REGOMA cohort, 9.2 months in the BRAIN cohort (a phase II trial with 167 patients), and 11.8 months with tumour treating fields (29, 203). This makes a four-point scoring system for the efficacy of the drug a less robust metric for recurrent disease.

Furthermore, there were inconsistencies in the measurement of survival. Only a proportion of studies reported the starting point of survival measurement, which was highly varied, including from diagnosis, randomisation or initiation of treatment. This limits confidence in comparing median OS and PFS between studies. Some studies relied on PFS-6 as an endpoint for an expedient measure of treatment efficacy, without reporting overall survival (94). While PFS-6 has been demonstrated to be a strong predictor of overall survival, true progression can be difficult to differentiate from pseudoprogression due to clinical and radiographic subjectivity. While most studies used either the Macdonald criteria or the Response Assessment in Neuro-Oncology (RANO), the criteria used to assess radiological progression was not reported in some studies (204). Due to surveillance scans on average being three months apart, there is also variability in the accuracy of the survival measurement from recurrence, which is amplified in glioblastoma due to the short average survival (205).

Studies used inconsistent classification systems for glioblastoma. For example, there is ambiguity in the historical nomenclature of “primary” and “secondary”, which have often been used to differentiate glioblastoma of *de novo* origin and progression from a lower grade tumour. Similarly, terms such as “progressive” and “non-progressive”, did not fit our preferred terminology of newly diagnosed and recurrent glioblastoma. Moreover, following the 2021 WHO update to the central nervous system tumour classification system such that only *IDH*-wildtype tumours are classified as glioblastoma, and former *IDH*-mutant glioblastoma are now classified as grade IV astrocytoma (206). Many of the reviewed studies did not provide this level of distinction in terms of the *IDH* mutation status of included patients, and it is likely that many patients would not have met the criteria for glioblastoma based on current definitions. In addition, there were some trials that were excluded due to studying mixed high grade glioma cohorts without reporting outcome data separately for each tumour type.

Trials studying bevacizumab as the sole RTK-targeted therapy were not included given the body of literature on the role of bevacizumab in prolonging PFS, but studies where it was used in combination with another RTK agent were included (207). We therefore note that there is a possible confounding effect of bevacizumab in several studies, notably in two trials studying erlotinib and a study of nimotuzumab which appeared beneficial to OS, and a study of rindopepimut with an observed survival benefit, such that the survival benefit cannot be attributed to the other RTK-directed therapy studied (99, 100, 104, 106).

Finally, this is a rapidly developing field with new trials likely to be published regularly, including possibly in the time since the search was performed for this review.

### Future directions

Future directions in the evaluation of RTK-directed drugs in glioblastoma should focus on the characterisation of predictive biomarkers.

Whilst RTK-directed therapies have generally failed to demonstrate a prolongation of survival with or without subgroup analysis of the effect of common gene alternations, further study of the small number of patients who do experience significant and longstanding benefit may help elucidate biomarkers predictive of response.

More precision in the definition of biomarker enrichment will also provide better evidence regarding its possible effect on treatment response. Enrichment strategy thresholds for target mutations tended to be unspecified or to consist of a relatively low percentage, such as 10% of tumour cells harbouring the target mutation, in the minority of cases where they were defined. Whether this is appropriate is unclear, with optimal target inclusion thresholds needing definition.

Biomarkers should also be identified contemporaneously alongside treatment (176, 178). This is often limited in studies of recurrent disease, for which only a minority of patients receive surgery, with biomarker analysis often relying on samples from the resected primary tumour (208, 209). An important future research goal is therefore to develop minimally invasive methods for monitoring changes in the biomarkers throughout treatment. Liquid biopsies, which include the detection of circulating biomarkers from tumour-derived cell free DNA in urine, blood or cerebrospinal fluid, is a promising minimally invasive method when compared to tissue biopsies (210, 211). Tissue biopsies are also limited by the region of tumour sampled, which may not be representative of the mutational landscape of spatially heterogenous tumours like glioblastoma and can be supplemented by liquid biopsies that ostensibly provide information of the tumour in its entirety (212).

The increasing accessibility of whole genome sequencing will also facilitate the identification of rarer biomarkers which may predict response to existing drugs. While *EGFRvIII* is the most prevalent mutation in glioblastoma, patients with mutations within the EGFR kinase domain may experience significant benefit from RTK-targeted therapy but are often not identified within trials (11). Novel methods such as three-dimensional tumour mapping with tissue and single cell sequencing has delineated drivers of tumour evolution and subclonal growth and identified potential targets which are activated tumour-wide (213). Moreover, novel clinical trial designs also aim to improve the quality of evidence, such as the 5G trial, an adaptive platform trial informed by whole genome sequencing data (214, 215).

Drugs targeting other rarer mutations such as *FGFR3-TACC*, which is present in approximately 3% of glioblastoma patients, could also be further explored (216). Whilst the FGFR pathway is targeted by multikinase inhibitors, our search did not identify trials of drugs specific to this pathway that reported glioblastoma survival outcome data.

More recent studies have also identified interactions between EGFR and MGMT, a DNA repair protein that repairs TMZ-induced DNA damage. A translational study found that EGFR inhibits *MGMT* transcription through NF-*κ*B, and that pre-treatment with EGFR inhibitors downregulates *MGMT* expression and increases TMZ sensitivity (217). As historic studies have largely focused on concomitant or monotherapy RTK-directed therapy regimens, future studies should further explore the role of sequential treatment strategies in patients with selected biomarkers, particularly in *MGMT*-unmethylated tumours where TMZ is less effective.

Finally, the development of novel drug delivery systems offers the possibility for re-evaluation of RTK drugs with poor blood-brain barrier penetrance. Survival benefit in animal models has been seen for nanoparticles, such as lipid-based polymers used to encapsulate drugs and tight junction-targeted nanoparticles with pulsed laser excitation that reversibly increase blood brain barrier permeability for the intravenous delivery of chemotherapy (218–220). Moreover, device-assisted methods such as magnetic resonance guided focused ultrasound provide greater spatial specificity and have demonstrated increased accumulation of chemotherapy drugs in tumour tissue (221). For small molecule RTK inhibitors, drug efflux mechanisms at the blood-brain barrier are a factor limiting distribution into the brain, and reassessment of these drugs alongside efflux transporter inhibitors, such as elacridar, may also be appropriate (181, 222, 223).

## Conclusion

Whilst there is currently no strong evidence that any single or combination of RTK-directed therapy provides superior survival outcomes compared to the current standard of care, a small proportion of therapies have shown encouraging results, including more modern multi-kinase inhibitors. Significant limitations include insufficient biomarker analysis in intervention cohorts and controls, uncertainty about the relevance of biomarkers used for targeted therapy, potency of novel compounds and efficacy of delivery of drugs into the brain. Improved genome sequencing capability, liquid biopsy and novel drug delivery approaches are possible solutions, in addition to better characterisation of the intratumoural heterogeneity contributing to therapeutic resistance.

## Data Availability

The original contributions presented in the study are included in the article/Supplementary Material, further inquiries can be directed to the corresponding author.
