# Supplementary material for: Receptor tyrosine kinase targeted therapies in glioblastoma: a systematic review
Source: Front Med (Lausanne). 2026 Sep 17;13:1928491. doi: 10.3389/fmed.2026.1928491 (PMC13628515; doi:10.3389/fmed.2026.1928491)
Supplement: Supplementary file 1 [file Datasheet1.docx]

Supplementary Table 1: Study Characteristics

Supplementary Table 2: Patient Characteristics

Supplementary Table 3: Survival

Appendix A: Search Strategies

Appendix B: Full Text Screening Exclusions

Appendix C: Patient and Study Characteristics Extracted

Appendix D: Risk of Bias Assessments

Appendix E: Study Funders

Appendix F: PRISMA Checklists

**Supplementary Table 1.** Summary of characteristics of included studies. R = randomised, NR = non-randomised, FT = full text, A= abstract, GB = glioblastoma, AA = anaplastic astrocytoma, RT = radiotherapy, CT = chemotherapy, KPS = Karnfosky Performance Status, P = primary, S = secondary, OS = overall survival, PFS = progression free survival, TTP = time to progression, ORR = objective response rate, DCR = disease control rate, PD = pharmacodynamic, PK = pharmacokinetic.

| Study  (NCT number) | Country | Trial design (Randomised/Non randomised) | Report format | Newly diagnosed or recurrent | Key inclusion criteria | Endpoint | Start of survival measurement |
| --- | --- | --- | --- | --- | --- | --- | --- |
| van den Bent 2009  (NCT00086879) | Netherlands | Phase II R | FT | R | histologically proven GBM recurrent disease after previous radiation no prior chemotherapy for recurrent disease or a maximum of only one prior chemotherapy regimen given as adjuvant treatment KPS ≥ 70 | P: PFS-6 S: response, survival, toxicity | / |
| Raizer 2009 | USA | Phase II NR | FT | Both | age >18 years life expectancy >8 weeks Karnofsky performance status (KPS) ≥60 no prior chemotherapy if newly diagnosed post RT | OS-12 | from registration |
| Yung 2010 | USA | Phase II NR | FT | R | GBM ≥18 years of age with histologically confirmed radiographic evidence of disease progression prior radiotherapy ECOG 0 or 1 archival tissue for EGFR analysis  no prior anti EGFR therapy  life expectancy >12 weeks | P: ORR S: response duration, PFS, PFS-6, survival | from first dose of erlotinib |
| Gallego 2014 | Spain | Phase II NR | FT | R | recurrent GBM  age >18 years life expectancy >8 weeks Karnofsky performance status (KPS) ≥60 no prior anti EGFR therapy | P: PFS-6, response S: OS, toxicity | from registration |
| de Groot 2008 | USA | Phase II NR | FT | R | age ≥ 18 years recurrent GBM no more than two prior chemotherapy regimens, failed radiation therapy with an interval of at least 4 weeks from completion of radiation treatment life expectancy greater than 8 weeks KPS ≥60 | PFS | from registration |
| Wen 2014  (NCT00112736) | USA | Phase II NR | FT | R | adults histologically confirmed supratentorial high-grade gliomas with tumor recurrence on MRI interval of ≥12 weeks from the completion of radiotherapy to study entry KPS ≥60 life expectancy ≥8 weeks | P: PFS6 | from registration or date of first post-surgery treatment for surgical arm |
| Peereboom 2010  (NCT00335764) | USA | Phase II NR | FT | ND | newly diagnosed GBM who were within 28 days of their diagnostic biopsy or resection at least 18 years old KPS at least 60 no prior administration of TMZ, tyrosine kinase inhibitors, cranial radiation, or concurrent EIAEDs , normal organ function | P: PFS, OS S: toxicity, feasibility of pharmacokinetics of dose | from study entry |
| Brown 2008  (NCT00039494) | USA | Phase I/II NR | FT | ND | newly diagnosed GBM age ≥ 18 years between 1-4 weeks after maximal surgical resection | OS-12 | from study registration |
| Prados 2009 | USA | Phase II NR | FT | ND | age ≥ 18 years of age KPS ≥ 60 newly diagnosed intracranial GBM or GS confirmed by biopsy or resection no more than 5 weeks before treatment. No prior treatment | P: OS  S: TTP | from diagnosis |
| Krishnan 2006 | USA | Phase I NR | FT | ND | newly diagnosed GBM age 18 years or older ECOG status 0,1,2 at least 1 week after but not more than 4 weeks after maximal surgical resection | P: MTD  S: TTP, OS, ORR | from study registration |
| Chakravarti 2013  (NCT00052208) | USA | Phase I/II NR | FT | ND | Age 18 or above Zubrod Performance scale 0-1 histopathologically confirmed supratentorial GBM estimated survival of at least 8 weeks no prior chemo or RT | / | / |
| Kreisl 2008 | USA | Pilot | FT | R | GBM KPS ≥70 age ≥18 years no limited on prior therapies | P: radiographic RR  S: PFS, molecular profile | / |
| Rich 2004 | USA | Phase II NR | Abstract | R | GBM in first relapse at least 18 years of age life expectancy of greater than 12 weeks KPS ≥ 60% nonincreasing dose of corticosteroids for at least 1 week | PFS-6; EFS, OS, tumour response rate, drug toxicity | from registration |
| Uhm 2011 | USA | Phase II NR | FT | ND | newly diagnosed adult (≥18 years)   ≥2 weeks but ≤5 weeks since the completion of radiation, and ≤15 weeks total since surgery/biopsy COG 2 or better | OS-12 | from initial surgery |
| Reardon 2015  (NCT00727506) | USA | Phase II randomised | FT | R | age of at least 18  histologically confirmed WHO grade 4 malignant glioma at first recurrence after temozolomide chemoradiotherapy bidimensionally measurable disease (tumor ≥10 mm in one diameter) KPS ≥70% | P: PFS-6 ORR, PFS, PKs, molecular determinants | from randomisation |
| Chi 2020 | USA | Phase II NR | FT | R | age ≥ 18 years histologically confirmed primary or secondary GBM EGFR amplification confirmed by FISH KPS >70% | P:PFS6 S: PFS, OS, radiographic RR, safety and tolerability | from study initiation |
| Sepulveda-Sanchez 2017 (NCT01520870) | Spain | Phase II NR | FT | R | age over 18 years histologically confirmed recurrent GB with EGFR amplification KPS ≥70 | P: PFS-6 S: safety, tolerability, OS, antitumour response | from registration |
| Solomon 2013 | Cuba | Phase II Randomised | FT | ND | maximal resection at least 4 weeks before inclusion age older than 18 KPS ≥ 60 | OS; PFS, response rate, safety, immunogenicity | / |
| Solomon 2014 | Cuba | Observational | FT | ND | age older than 18 y KPS greater or equal to 50 time from surgery between 4 and 6 wk | safety | / |
| Westphal 2015 | Germany | Phase III RCT | FT | ND | histologically confirmed GBM age 18-70 KPS ⩾70 | P: PFS S: OS, toxicity, tumour response, quality of life | / |
| Wang 2016 | China | Prospective | FT | ND | GBM diagnosed by histology ≥18 years old patients  KPS ≥50 | PFS-6 | from initiation of RT |
| Du 2019 | China | Phase II NR | FT | ND | age 18-75 newly diagnosed supratentorial GBM with EGFR expression >50% of the gross tumor volume removed by surgery  KPS ≥ 60 | P: OS, PFS S: ORR, toxicity | from surgery |
| Ramos 2006 | Cuba | Phase I/II | FT | Unknown | GBM or AA undergone biopsy alone or debulking surgery  age more than 18 years KPS >70 no previous anti EGFR antibodies | / | from first treatment |
| Desjardins 2017  (NCT02540161) | USA | Phase II NR | Abstract | R | recurrent GBM, EGFR amplified (>15% of cells exhibiting >5 copies of EGFR loci); ≤3 prior progressions; ≥4 weeks after chemotherapy, or study drug; ≥12 weeks after radiation; adequate organ function; and KPS ≥70% | PFS-6 | / |
| Brady 1992 | USA | Phase II NR | FT | ND | GBM with no prior treatment | Survival | / |
| Li 2010  (NCT00589706) | USA | Phase II R | FT | ND | newly diagnosed GBM | OS, toxicity | / |
| Spiekman 2023  ([NCT02925234](https://clinicaltrials.gov/ct2/show/NCT02925234)) | Netherlands | Prospective multicentre basket | FT | R | clinical and radiological evidence for refractory RAF/RASwt recurrent glioblastoma with molecular testing demonstrating no mutations in either BRAF, KRAS, or NRAS and stable or decreasing dosage of steroids for at least 7 days prior to the baseline magnetic resonance imaging | Clinical benefit; ORR, PFS, OS | from start of treatment |
| Choi 2023  (NCT02352571) | Korea | Phase II | FT | R | ≥19 years of age with life expectancy of ≥3 months, Karnofsky performance status score ≥70, and adequate bone marrow and end‐organ function | P: PFS6 S: PFS, OS, radiographic response rate, safety | / |
| Gan 2018  (NCT02343406) | Australia | Phase I NR | FT | Both | KPS ≥70 for arm B: with newly diagnosed glioblastoma had completed postoperative RT and concurrent TMZ but had not progressed for arms B and C with recurrent GBM had disease progression and a gap of at least 12 weeks after RT | / | from first dose of depatux-M |
| Lassman 2019  (NCT01800695) | USA | Phase I NR | FT | R | adults EGFR amplified recurrent/progressive GBM  bev naïve KPS of at least 70 | / | from first dose of depatux-M |
| Caccese 2020 | Italy | Retrospective | Abstract | R | Histologically confirmed GBM EGFR-amplified one or more prior systemic therapies ECOG PS<2 | P: OS, safety S: PFS | / |
| Reardon 2013 | USA | Phase I NR | Abstract | R | reccurent, EGFR-amplified GBM with emasurable disease | / | / |
| Van den Bent 2020 (NCT02343406) | Netherlands | Phase II | FT | R | 18 years or older glioblastoma with EGFR amplification relapsing more than 3 months after the end of radiotherapy no prior treatment with nitrosourea, bevacizumab, or EGFR targeting agents for those reoperated for the recurrence needed to have a bidimensionally measurable enhancing lesion with minimal square diameters of 10 mm on MRI stable or decreasing dose of steroids | P:OS  S: OS in EGFRvIII cohort, ORR, PFS | from randomisation |
| Lassman 2023 (NCT02573324) | USA | Phase III RCT | FT | ND | age ≥18 years old KPS≥70 RT and chemotherapy naïve  unifocal GBM EGFR-amp | P: OS S: PFS, moelcular subgroup | from randomisation |
| Schuster 2015 (NCT00458601) | USA | Phase II | FT | ND | 18 years of age with newly diagnosed, de novo glioblastoma confirmed EGFRvIII+, KPS > 70% | P: PFS5.5  S: OS, safety, immune response | from study entry |
| Sampson 2010 | USA | Phase II | FT | ND | adults with newly diagnosed EGFRvIII-expressing GBM with a gross total resection (> 95%) and a Karnofsky performance status (KPS) ≥ 80% who had no radiographic evidence of progression after standard of care external beam radiation therapy and concurrent temozolomide | PFS | from histological diagnosis |
| Weller 2017 (NCT01480479) | Switzerland | Phase III RCT | FT | ND | patients aged 18 years and older newly diagnosed EGFRvIII-expressing glioblastoma undergone maximal surgical resection and have completed standard radiotherapy (up to 60 Gy) with concomitant temozolomide (75 mg/m2 per day) | P: OS PFS: PFS, safety | from randomisation |
| Goff 2019 (NCT01454596) | USA | Pilot | FT | R | adult EGFRvIII+ glioblastoma with radiologic recurrence after initial surgical resection or chemoradiotherapt KPS ≥60% | safety, PFS | / |
| Bagley 2024 (NCT03726515) | USA | Phase I | FT | ND | histopathologically confirmed GBM according to the revised World Health Organization 2021 classification19 and had already undergone resection, adequate performance status, no severe or uncontrolled autoimmune disease and tumor tissue negative for MGMT promoter methylation | P: safety S: PFS, OS | from registration |
| Gerstner 2015 ([NCT00979862](https://clinicaltrials.gov/ct2/show/NCT00979862)) | USA | Phase I | FT | R | recurrent GBM, age ≥18 years, Karnofsky performance status score ≥60%, mini-mental score >15, at least 1 cm of residual enhancing disease, at least 3 months since radiation, ≤2 prior tumor relapses, stable steroid dose for 5 days prior to baseline MRI, and adequate bone marrow/organ function | P: S S: OS, PFS-6, radiographic response | from start of treatment |
| Batchelor 2010 ([NCT00305656](http://clinicaltrials.gov/show/NCT00305656)) | USA | Phase II NR | FT | R | Recurrent glioblastoma  age ≥ 18 years KPS ≥ 60 MMSE ≥ 15 prior therapy with radiation treatment with ≤ 2 chemotherapy regimens stable dose of corticosteroids for ≥ 5 days before the first baseline MRI scan  ≥ 3 months since completion of radiation f ≥ 3 weeks since completion of a non-nitrosourea chemotherapy   ≥ 6 weeks since completion of a nitrosourea-based chemotherapy | P: APF6 S: radiographic RR, OS, toxicity | / |
| Batchelor 2013  ([NCT00777153](https://clinicaltrials.gov/ct2/show/NCT00777153)) | USA | Phase III RCT | FT | R | Pathological diagnosis of recurrent glioblastoma >18 years prior treatment with temozolomide-containing radiotherapy regimen and radiation MMSE>15 KPS>70 life expectancy >12 weeks | P: PFS S: OS, APF6, radiographic RR, steroid-sparing effects, safety, tolerability, time to deterioration in neurologic status | from randomisation |
| Batchelor 2023 ([NCT01062425](http://clinicaltrials.gov/show/NCT01062425)) | USA | Phase II | FT | ND | age ≥ 18 years, pathological diagnosis of glioblastoma, and Karnofsky Performance Status (KPS) ≥ 70 | P: PFS6 S: PFS, OS, toxicity | from randomisation |
| Hsieh 2022 (NCT02974621) | USA | Phase II | Abstract | R | Bevacizumab-naïve adult patients with first or second recurrence of glioblastoma | / | / |
| Duerinck 2016 (NCT01562197) | Belgium | Phase II | FT | R | histologically confirmed GBM, age ≥ 18, recurrence for progression after surgery, RT and alkylating chemotherapy including TMZ, WO-PS equal or less than 2, life expectancy great erthan 12 weeks | PFS-6 | from randomisation |
| Duerinck 2018 (NCT01562197) | Belgium | Phase II Randomised | FT | ND | histologically confirmed GBM aged ≥ 18 years or older and have tumor recurrence or progression following prior treatment with surgery, radiation therapy and temozolomide | PFS-6 | from randomisation |
| Awada 2020 (NCT03291314) | Belgium | Phase II NR | FT | R | Histologically confirmed diagnosis of WHO Grade IV malignant glioma (glioblastoma or gliosarcoma)  age equal or greater than 18 years estimated life expectancy of at least 3 months ECOG PS 0 or 1 | PFS-6 | / |
| Ge 2020 | China | Retrospective | FT | R | age 18-70 KPS ≥50% histological diagnosis of glioblastoma recurrence was confirmed histologically or by radiographic evidence no previous treatment with bevacizumab or other antiangiogenic drugs treated with at least one cycle of the combined regimen of apatinib and temozolomide | PFS-6, ORR, PFS, OS, 12m-OS | from treatment initiation |
| Wang 2019 ([NCT03660761](https://clinicaltrials.gov/ct2/show/NCT03660761)) | China | Phase II | FT | R | age of 18–70 years  KPS of ≥60 histologically confirmed GBM, World Health Organization Grade IV minimum life expectancy of 8 weeks progressive disease (relapse) on MRI defined by RANO criteria after the standard Stupp protocol the time interval for the start of treatment was at least 12 weeks from prior radiotherapy unless in the presence of histopathologic confirmation of recurrent tumor or new contrast enhancement on MRI outside of the radiotherapy treatment field | P: PFS S: OS, ORR, DCR | from beginning of intervention |
| Zhang 2018 | China | Phase II | Abstract | R | / | PFS6 | / |
| Zhang 2022 | China | Single arm non randomised /retrospective | FT | R | GBM   recurrence or progression after conventional standard treatments including surgery, radiotherapy, and temozolomide at least one measurable or assessable tumor lesion by MRI | / | from apatinib treatment |
| Schiff 2015 (NCT00562419) | USA | Phase II | FT | R | at least 18 years histologically confirmed diagnosis of a recurrent/progressive GBM and presenting in first, second, or third relapse evidence of bi-dimensionally measurable recurrent or residual tumor on MRI  KPS≥70 % | P: PFS6 S:PFS-12, OS-6, OS-12, PFS, OS, ORR | / |
| Kalpathy-Cramer 2017 (NCT01846871) | USA | Phase II | FT | R | histologically confirmed GBM that had progressed or recurred age ≥ 18 years KPS ≥ 60 measurable disease at least 3 months since radiation stable steroid dose for at least 5 days prior to baseline MRI ≤ 3 prior tumor relapses, and mini-mental score > 15 | P: PFS6 OS: radiographic RR, steroid use, toxicity | / |
| Baumann 2004 | Switzerland | Phase II NR | FT | Both (80% ND, 20% R) | 18-75 years histologically proven GBM | Survival, TTP | from diagnosis |
| Chang 2004 | USA | Phase II NR | FT | ND | Age 18 or above, histopathologically confirmed newly diagnosed previously untreated GBM | OS | from diagnosis |
| Marx 2001 | Australia | Phase II NR | FT | R | supratentorial histologically confirmed grade IV glioma (GBM) and radiological evidence of progressive disease age (18–80) KPS (>40) life expectancy of at least 2 months and measurable disease on CT or MRI | / | / |
| Fine 2003 | USA | Phase II NR | FT | R | 18 years of age and older with recurrent high-grade gliomas KPS ≥ 60 previously treated with at least standard fractionated external beam radiation maximum of two prior chemotherapy regimens, including a nitrosourea | PFS, ORR | from start of treatment |
| Fadul 2008 | USA | Phase II NR | FT | Both | age ≥18 years KPS ≥ 60 histologically confirmed GBM | P: tumour response S: toxicity, PFS, OS | from study initiation |
| Riva 2007 | USA | Pilot | FT | ND | adult newly diagnosed, supratentorial GBM | OS | / |
| Groves 2007 | USA | Phase II | FT | R | WHO grade IV GBM with progression after radiation therapy (RT); no more than one adjuvant chemotherapy; no more than one treatment with chemotherapy for recurrence, no prior TMZ or thalidomide, KPS ≥60, ≥18 years old with a life expectancy ≥8 weeks | P: PFS6, S: MTP, radiographic response | from first dose of drug |
| Kesari 2008 (NCT00047294) | USA | Phase II NR | FT | ND | Adult (age 18 or above) glioblastoma, KPS 60-100% | P: 4 month PFS; S: OS | from enrolment |
| Alexander 2013 | USA | Phase II NR | FT | ND | patients 18 years of age or older with untreated supratentorial glioblastoma confirmed by biopsy or excision estimated survival time of at least 8 weeks KPS of 60 or higher | OS, PFS, toxicity | from enrolment |
| Penas-Prados 2015 ([NCT00112502](http://clinicaltrials.gov/show/NCT00112502)) | USA | Phase II NR | FT | ND | histological diagnosis of supratentorial GBM in adults (aged ≥18 years), a KPS ≥ 60%, any extent of resection, and successful completion of standard chemoradiation without tumour progression | P: PFS S: OS | from randomisation |
| Groot 2011 ([NCT00369590](https://clinicaltrials.gov/ct2/show/NCT00369590)) | USA | Phase II | FT | R | histologically confirmed glioblastoma, gliosarcoma, or anaplastic glioma with evidence of unequivocal progression after chemoradiotherapy and no more than one adjuvant temozolomide-containing regimen, KPS ≥ 60 | P: PFS6 S: radiographic response rate, TTP, OS, toxicity | from study registration |
| NCT00895180 2017 | USA | Prospective | Trial results | R | / | / | from start of treatment |
| Nabors 2010 (NCT00584883) | USA | Phase I | FT | ND | 19 years or older, histologically proven GBM recovery from toxicities of previous therapies, treated with a stable dose of corticosteroids for 5 or more days KPS of 60% | / | from start of treatment |
| Brown 2016 ([NCT01310855](https://clinicaltrials.gov/ct2/show/NCT01310855)) | UK | Phase II randomised | FT | R | GBM prior treatment of surgery, cranial radiotherapy + concurrent and adjuvant temozolomide only ≥ 18 years life expectancy ≥ 12 weeks KPS ≥ 70 mini-mental status examinations core ≥ 15; presence of measurable tumor seven days prior to enrolment stable dose of steroids | P: PFS S: OS, Radiological RR | from randomisation |
| D'alessandris 2013 | Italy | Pilot | FT | ND | age ≥ 18 years GBM ≥4 weeks from surgery and prior radio-chemotherapy radiological evidence of recurrence or progression of disease after previous treatments KPS ≥ 70 | P: RR, PFS6 S: PFS, OS | from treatment initiation |
| D'alessandris 2019 | Italy | Pilot | Abstract | R | Recurrent GBM EGFRvIII expression and normal PTEN | PFS-6, RR | / |
| Sathornsumetee 2010 ([NCT00671970](http://clinicaltrials.gov/show/NCT00671970)) | USA | Phase II | FT | R | recurrent WHO grade III (anaplastic astrocytoma, oligodendroglioma, oligoastrocytoma or pleomorphic xanthoastrocytoma) or WHO grade IV MG (GBM or gliosarcoma), ≥18 years; ≥4 weeks from craniotomy (1 week for biopsy), prior radiotherapy, or chemotherapy (6 weeks for a nitrosourea); Karnofsky performance status (KPS) score ≥60; and adequate hematologic, hepatic, and renal function. | PFS-6; OS, radiographic response, pharmacokinetics, correlative biomarkers | time from treatment initiation |
| Clarke 2014 | USA | Phase II | FT | ND | Adult, KPS>60, newly diagnosed surgically confirmed glioblastoma or gliosarcoma, with study treatment starting 3-5 weeks after open surgery or 2-5 weeks after biopsy | OS | / |
| Cardona 2021 | Colombia | Retrospective | FT | R | Adult  GBM with EGFR amplification and EGFRvIII mutation | PFS6, ORR, OS, safety | from first dose of osimertinib/bevacizumab |
| Wu 2025 |  | Retrospective | FT | ND | Histopathological confirmation of GBM, IDH mutational status: Wild type, No prior history of chemotherapy or radiotherapy, KPS ≥ 70, indicating preserved functional status, Normal bone marrow function, Normal liver function | OS, PFS | from diagnosis/date of surgery |
| Hasselbalch 2010 | Denmark | Phase II NR | FT | ND | adult (age ≥18 years) with histologically proven primary GBM (WHO classification) MRI-verified recurrent or progressive disease (PD) WHO performance status 0–2 ≥4 weeks from prior surgery and/or chemotherapy life expectancy >3 months | PFS-6 | / |
| Reardon 2020 (NCT01498328) | USA | Phase II | FT | R | at least 18 years first or second relapse of histologically confirmed glioblastoma following standard therapy EGFRvIII expression in primary or recurrent tumor tissue no previous bevacizumab or VEGF or VEGFR targeting agents KPS >70% | P: PFS-6 S: OS, ORR, steroid dose reduction | / |
| Phuphanich 2017 (NCT01268566) | USA | Phase II NR | Abstract | R | age of at least 18 WHO grade IV astrocytoma previous first-line therapy with radiation and temozolomide ECOG 0 or 1 life expectancy of ≥12 weeks | P: PFS6 S: ORR, OS, safety, tolerability | from start of treatment |
| Andrews 2021 (NCT02507583) | USA | Phase Ib | FT | ND | 18 years or old radiographically diagnosis of unifocal, multifocal, or bihemispheric glioblastoma KPS of at least 60 or ECOG 1/2/3 | Safety, tumour response, PFS, OS | from date of surgery |
| Affronti 2018 ([NCT01113398](https://clinicaltrials.gov/ct2/show/NCT01113398)) | USA | Phase II NR | FT | R | Recurrent malignant glioma WHO grade IV | ORR, OS, PFS, toxicity | / |
| Wen 2011 | USA | Phase II | FT | R | aged ≥18 years, histologically confirmed GBM or gliosarcoma, Karnofsky performance score ≥60%, ≤3 prior relapses or systemic treatments | P: ORR S: adverse events, PFS, OS, time to response, duration response | from first dose of rilotumumab |
| Cloughesy 2017 | USA | Phase II Randomised | FT | R | age at least 18 years KPS ≥ 70%  histologically confirmed glioblastoma at first recurrence after concurrent or adjuvant chemoradiotherapy | PFS, OS, OS-9, PFS-6, OR, duration of response, safety | / |
| Lombardi 2019 ([NCT02926222](http://clinicaltrials.gov/show/NCT02926222)) | Italy | Phase II Randomised | FT | R | histologically confirmed diagnosis of glioblastoma prior first line therapy according to the Stupp protocol age >18 years  ECOG performance status score ≤1 | P: OS S: PFS | from randomisation |
| Lombardi 2021 | Italy | Prospective | FT | R | histologically confirmed diagnosis of glioblastoma prior first line therapy according to the Stupp protocol age >18 years  ECOG performance status score ≤1 | P: OS S: PFS | from start of treatment |
| Caccesse 2024 | Italy | Prospective | FT | R | histological diagnosis of glioblastoma by WHO 2016 criteria with disease progression/recurrence after radiochemotherapy on RANO criteria; age ≥18 years; good general clinical status ECOG PS 0-1) | PFS; OS, ORR, DCR, safetey | from enrolment |
| Tünbekici 2024 | Turkey | Prospective | FT | R | histologically confirmed isocitrate dehydrogenase (IDH)-wildtype glioblastoma, progression after second-line bevacizumab-based treatment, and an ECOG performance status score of ≤2 | / | from start of regorafenib treatment |
| Mazzarella 2022 | Italy | Retrospective | Abstract | R | patients with GBM recurrence treated with regorafenib | / | / |
| Lai 2024 ([NCT04119674](https://clinicaltrials.gov/ct2/show/NCT04119674)) | China | Phase II NR | FT | ND | age 18-75, newly diagnosed GBM, surgery within 6 weeks before enrollment, no pervious exposure to other therapies, KPS ≥ 60 %, had at least one measurable lesion according to RANO criteria, and adequate bone marrow, hepatic, and renal functions | PFS | From enrolment |
| Wang 2024 | China | Retrospective | FT | R | 1) recurrent grade 4 glioma based on WHO classification 2) age ≥18 years old 3) previously standard surgery, radiotherapy, and TMZ chemotherapy; 4) treatment with anlotinib after recurrence; 5) at least one measurable lesion 6) recurrence evaluated according to RANO criteria and 7) no other systemic diseases | / | from first diagnosis |
| Yang 2021 | China | Retrospective | FT | R | histologically confirmed GBM relapsed or progressive  18-75 years of age KPS ≥ 60% | PFS6 | / |
| Guan 2022 ([NCT04197492](https://clinicaltrials.gov/ct2/show/NCT04197492)) | China | Retrospective | FT | R | / | P: ORR S: OS, PFS | from completion of RT |
| Cao 2021 | China | Retrospective | Abstract | R | / | / | / |
| She 2021 | China | Retrospective | FT | R | KPS ≥ 60; ≥18 years old; pathologically confirmed as GBM WHO Grade IV; recurrence treated with anlotinib combined with dose-dense TMZ; previously standard CCRT and AC | PFS6, OS-12 | from start of anlotinib treatment |
| Yang 2022 | China | Phase II | FT | R | histologically confirmed GBM, relapsed or progressive, 18-75 years of age, KPS ≥ 60% and adequate organ function | PFS6 | / |
| Zhao 2024 ([NCT04822805](https://clinicaltrials.gov/ct2/show/NCT04822805)) | China | Phase II | FT | R | Age ≥ 18 years old; ECOG 0-2, Evaluable intracranial lesions defined as contrast-enhancing tumors with a minimum diameter of 10 mm on two axial slices | PFS; OS, ORR | from start of anlotinib treatment |
| Hainsworth 2010 | USA | Pilot | FT | ND | histologically confirmed supratentorial GBM age ≥18 years ECOG status 0 or 1 | PFS | from first day of treatment |
| Hassler 2014 | Austria | Retrospective | FT | R | histologically confirmed GBM | TTP | from start of sorafenib |
| Zustovich 2013 | Italy | Phase II NR | FT | R | age of 18 years or older prior histological diagnosis of GBM documented radiological relapse during or after first-line treatment with radiotherapy plus temozolomide evaluable disease according to RANO criteria  ECOG 2 or less life expectancy longer than eight weeks | PFS6 | / |
| Galanis 2013 (NCT00621686) | USA | Phase II NR | FT | R | age ≥18 years, histologically confirmed GBM stable dose of corticosteroids or no corticosteroids for ≥ 1 week before baseline imaging last chemotherapy treatment ≥ 4 weeks before study entry ECOG performance score 0-2 | PFS-6 | / |
| Schiff 2018 (N0572) | USA | Phase I/II | FT | R | confirmed GBM ≥18 years of age with ≤2 prior systemic chemotherapy regimens, had radiographically confirmed tumour progression ECOG performance status ≤2 ≥12 weeks from completion of radiotherapy ≥4 weeks from prior cytotoxic chemotherapy (≥6 weeks for nitrosoureas), stable or decreasing corticosteroid dose | PFS-6 | from start of therapy |
| Peereboom 2013 (NCT00335764) | USA | Phase II | FT | R | age at least 18 years, measurable, histologically proven GBM that had progressed or recurred following radiation therapy and 0–2 prior chemotherapy regimens, KPS ≥60% and normal organ function, | OS; PFS6, radiographic response rate, toxicity, pharmacokinetics | from first day of treatment |
| Chen 2020 (NCT00335764) | USA | Phase I/II | FT | R | patients ≥18 years of age with histologically confirmed intracranial GBM or gliosarcoma, KPS ≥ 60 | PFS6 | / |
| Dresemann 2010 (NCT00154375) | Germany | Phase III RCT | FT | R | adult  histologically confirmed GBM measurable diseas ECOG equal or less than 2 previously undergone surgery and received radiotherapy and prior chemotherapy | PFS | / |
| Reardon 2005 (NCT00290771) | USA | Phase II NR | FT | R | recurrent GBM ≥ 18 years old have measurable, contrast-enhancing tumor on MRI  KPS ≥ 60% stable corticosteroid dose for ≥ 1 week | PFS6 | from treatment initiation |
| Wen 2006 (NCT00010049) | USA | Phase II NR | FT | R | adults (≥18 years old) with histologically confirmed supratentorial malignant gliomas with unequivocal tumor recurrence by MRI KPS ≥60 life expectancy ≥8 weeks | PFS6, response | from registration |
| Raymond 2008 | Netherlands | Phase II NR | FT | R | age ≥ 18 years a progressive glioma after prior radiotherapy and documented by MRI or CT within 2 weeks from the start of treatment stable or decreasing doses of corticosteroids for at least 2 weeks before baseline scan ECOG performance status ≤ 2 at least one bidimensionally measurable contrast-enhancing target lesion with at least one diameter of at least 2 cm no brain irradiation in the last 3 months and no dosage of radiotherapy more than 65 Gy, no brain surgery within the last 3 months | P: ORR S: duration of response, toxicity, PK analysis | / |
| Hassler 2014 | Austria | Retrospective | FT | R | Recurrent GBM recurrent during or shortly after treatment with alkylating agents equal or less than three months after initial treatment aged 18 or older, performance status ≤ 2 WHO score on | P: OS  S: PFS, PFS-6 | from start of imatinib |
| Razis 2009 | Greece | Phase iI NR | FT | ND | age > 18 y, measurable disease by magnetic resonance imaging  life expectancy of ≥3 mo  GBM | / | / |
| Sautter 2020 | Germany | Phase II NR | FT | Both | age 18 years or older with an ECOG performance status of 0–2 that were either newly diagnosed (arm A) with a measurable tumor (i.e., after incomplete resection or biopsy) or that were diagnosed with progression of a glioblastoma after initial therapy (arm B) | PFS, safety | from initial diagnosis (IG1 primary) or from initiation of imatinib (IG2 reccurent) |
| Reardon 2009 (NCT00290771) | USA | Phase II NR | FT | R | histological confirmation of GBM that was at first recurrence after conventional therapy and measurable disease on gadolinium-enhanced MRI  ECOG score ⩽2 or Karnofsky ⩾60% | P: radiographic response rate S: safety, PFS-6, OS | / |
| Kreisl 2012 | USA | Phase II | FT | R | Adult recurrent glioblastoma | P: PFS6 S: QoL measures | / |
| Dresemann 2005 | Germany | Prospective | FT | R | age 16 or older WHO grade IV glioblastoma multiforme Prior radiotherapy with 50–60 Gy, no radiotherapy treatment option, Prior temozolomide and nitrosourea containing chemotherapy No neuro-surgical treatment option Confirmed tumour progression | / | / |
| Balana 2014 (NCT01100177) | Spain | Phase II NR | FT | ND | age 75 years or under GBM not amenable to surgical resection no previous chemotherapy or radiotherapy, stable doses of dexamethasone over the week prior to inclusion, performance status (PS) ≤2, mini mental status (MMS) ≥25/30, Barthel index (BI) >50 % | P: ORR, sunitinib  S: PFS, OS, OS-12, % patients completing radiotherapy patients free of neurological deterioration pre-radiotherapy | / |
| Pan 2012 (NCT00499473) | USA | Phase II NR | FT | R | age ≥18 years KPS ≥60 pathologically or neuroradiographically recurrent AA or GB and prior pathologic confirmation of primary tumour histology | P: PFS6 S: PFS, OS | from first dose of sunitinib |
| Hutterer 2014 ([NCT00535379](http://clinicaltrials.gov/show/NCT00535379)) | Germany | Phase II NR | FT | R | first recurrence of a histologically proven supratentorial GBM diagnosed by MRI according to Macdonald criteria 14 or by histological confirmation following re-resection, | P: PFS6 S: PFS, OS | from study entry |
| Janssen 2024 ([NCT03025893](https://clinicaltrials.gov/ct2/show/NCT03025893)) | Netherlands | Phase II/III | FT | R | adults with histologically confirmed de novo or secondary glioblastoma with first progression/recurrence, who received one prior line of chemotherapy, KPS ≥70% | PFS | from randomisation |
| Faye 2023 (NCT02928575) | Canada | Phase II | FT | ND | Patients 18–70 years of age with newly diagnosed and histologically confirmed GBM and an unmethylated MGMT promoter status, KPS 70 or higher, no prior history of brain tumors, RT to the brain, chemotherapy or antiangiogenic therapy, and a life expectancy of at least 6 months | P: PFS S: OS, adverse effects | from diagnosis |
| Martinez-Garcia 2022 ([NCT02270034](https://clinicaltrials.gov/ct2/show/NCT02270034)) | Spain | Phase IB | FT | ND | age >18 years diagnosed with histologically confirmed glioblastoma in the 4–7 weeks prior to patient registration KPS ≥60% | P: MTD S: ORR, PFS, OS | / |
| Sharma 2019 (NCT01753713) | USA | Phase II | FT | R | age of at least 18 years, histologically confirmed GBM recurrent after standard external-beam fractionated radiotherapy, temozolomide chemotherapy with or without anti-angiogenic therapy KPS ≥ 60% estimated survival of at least 2 months | P: PFS6, TTP S: toxicity | from start of treatment |
| Lee 2019 (NCT02478164) | USA | Phase II | FT | R | adults (age ≥ 18 years old) with histologically confirmed GBM or variants who progressed following an anti‐VEGF(R) containing regimen KPS ≥60 | P: PFS3 S: radiographic RR, OS, PFS, safety | / |
| NCT01137604 |  | Prospective | Clinical trials.gov results | R | / | PFS6, PFS, ORR, OS, DCR, CBR, safety | from randomisation or first day of treatment |
| Rha 2025 ([NCT03797326](https://clinicaltrials.gov/ct2/show/NCT03797326)) | Korea | Phase II | FT | R | WHO grade IV GBM, ≥3 weeks from prior surgical resection, ≥1 week from stereotactic biopsy, ≥6 months from prior radiotherapy , ≥6 weeks from prior antibody treatment, ≥2 weeks from Optune TTFields treatment, and ≥4 weeks from any investigational agents, cytotoxic therapies, daily administered chemotherapeutics, or any other antitumor therapies. PD‐L1‒evaluable tumour tissue sample, stable neurologically and clinically, ECOG ≤1 | P: ORR, safety S: DCR, duration of response, PFS, OS | from treatment initiation |
| Reardon 2013 (NCT00350727) | USA | Phase I/II NR | FT | R | at least 18 years ECOG status 0 or 1 grade 4 malignant glioma at first or second recurrence no concurrent EIAC therapy | P: PFS-6 S: PFS, pharmacokinetics | / |
| Iwamoto 2010 (NCT00459381) | USA | Phase II | FT | R | ≥18 years old with histologically confirmed glioblastoma or gliosarcoma and unequivocal radiographic tumour progression, KPS ≥60, adequate organ function, life expectance >8 weeks | PFS6 | from registration |
| Lee 2015 ([NCT00441142](https://clinicaltrials.gov/ct2/show/NCT00441142)) | USA | Phase II randomised | FT | ND | age ≥ 18 years, Karnofsky performance status (KPS) ≥ 60, and not on enzyme-inducing antiepileptics | OS, PFS, 12 month PFS, safety | from randomisation |
| McNeill 2014 | USA | Phase II | Abstract | R | / | PFS6 | / |
| Kreisl 2012 ([NCT 00272350](https://clinicaltrials.gov/ct2/show/NCT00272350)) | USA | Phase I/II | FT | R | age ≥18 and histologically confirmed GBM, gliosarcoma, anaplastic astrocytoma, anaplastic oligodendroglioma, anaplastic mixed oligoastrocytoma, malignant glioma/astrocytoma not otherwise specified diagnosed radiographically | PFS6 | from study registration |
| Chheda 2015 (NCT00821080) | USA | Phase I | FT | R | adults (age 18 or above), with recurrent GBM on no CYP3A4-inducing drugs, previously treated with standard therapy naïve to prior anti-VEGF, anti-EGF therapy or mTOR inhibitors KPS ≥60 %  MMSE score >15 at least 3 months from the completion of radiation or radiosurgery and must have been maintained on a stable or decreasing corticosteroid regimen for at least 3 days prior to the start of treatment | MTD, APF6 | / |
| Franceschi 2012 ([NCT 00948389](https://clinicaltrials.gov/ct2/show/NCT00948389)) | Italy | Phase I/II | FT | R | histologically verified GBM recurrent after standard RT with concomitant and adjuvant TMZ | PFS6 | From enrolment |
| Breen 2025 (NCT00869401) | USA | Phase II randomised | FT | ND | Adults (age ≥ 18 years) with newly diagnosed histologic glioblastoma (WHO 2007 classification) with ECOG score of 0–2 | P: OS; S: PFS, toxicity, QoL | From registration |
| Lu-Emerson 2011 | USA | Retrospective | FT | R | Adult patients with histologically confirmed GBM who failed bevacizumab therapy | / | / |
| Lassman 2015 ([NCT00423735](https://clinicaltrials.gov/ct2/show/NCT00423735)) | USA | Phase II NR | FT | R | KPS ≥60%; no concurrent hepatic enzyme-inducing anticonvulsants; prior treatment with surgery, radiotherapy, and temozolomide exclusively; and activation or overexpression of ≥2 putative dasatinib targets in GBM (ie, SRC, c-KIT, EPHA2, and PDGFR) | PFS6, radiographic RR | / |
| Muhic 2013 | Denmark | Phase II NR | FT | R | age >18 years ECOG performance status of 0–1 and histologically proven GBM previously failed standard radiotherapy plus TMZ as first-line therapy (STUPP IG1), or the same regimen with subsequent bevacizumab-based therapy as second-line treatment (BEV IG2) | P: ORR S: PFS, OS, safety, biomarkers | from start of treatment |
| Norden 2015 (NCT01380782) | USA | Phase II NR | FT | R | age of at least 18 years old, KPS 60 or higher, histologically confirmed high-grade glioma with unequivocal evidence of progression and had received treatment for no more than 2 prior relapses | PFS6 | / |
| Wen 2018 (NCT00704288) | USA | Phase II NR | FT | R | age ≥ 18 year  progressive or recurrent GBM confirmed by MRI  prior RT and TMZ KPS ≥ 60 prior angiogenic therapy | P: OSS S: PFS6, OS, safety | from first dose of cabozantinib |
| Odia 2016 ([NCT00667394](https://clinicaltrials.gov/ct2/show/NCT00667394)) | USA | Phase II NR | FT | R | age of at least 18 years pathologically confirmed GBM KPS of 60% at least  expected survival of 2+ months | PFS6 | / |
| Batchelor 2017 ([NCT00379080](https://clinicaltrials.gov/ct2/show/NCT00379080)) | USA | Phase I/II NR | FT | R | Pathological diagnosis of recurrent glioblastoma >18 years prior treatment with radiation MMSE>15 KPS>60 | Radiographic RR, PFS6 | / |

**Supplementary Table 2.** Summary of patient characteristics for included studies. The percentage of studies reporting each data point is in brackets below the column headings. KPS = Karnofsky Performance Status, ORR = objective response rate, MGMT = methylguanine methyltransferase, RT = radiotherapy, TMZ = temozolomide, IG = intervention group, CG = control group.

| Study | No. of patients | Subgroups | Median age | Age range | Baseline median KPS | % White ethnicity | % Male | % Complete resection | % MGMT promoter methylation | % ORR | Average time from diagnosis to therapy |
| --- | --- | --- | --- | --- | --- | --- | --- | --- | --- | --- | --- |
| van den Bent 2009 | 110 | IG=54 CG-56 | IG=55 CG=54 | IG=18.7-71.4 CG=19.5-78.8 | / | / | IG=64.8 CG=66.1 | / | / | / | / |
| Raizer 2009 | 87 | rGBM=42 NP GBM= 45 | rGBM=54 NP GBM = 52 | rGBM=29-78 NP GBM=19-72 | rGBM=80 NP GBM=90 |  | rGBM=57.6 NP GBM=77.8 | GBM=44.4% | / | / | / |
| Yung 2010 | 48 | EGFR amplified = 23 Not amplified=25 | EGFR amplified IG1 =54 Not amplified IG2 =50 | IG1 = 38-70 IG2 = 37-73 | / |  | / | IG1 =47.8 IG2 =32 | / | IG1 =5 IG2 =10.7 | 6.3m |
| Gallego 2014 | 14 | / | 53 | / | 80 |  | 50 | / | / | 10 | / |
| de Groot 2008 | 43 | / | 53 | 23-71 | 80 | / | 65 | 35 | / | / | / |
| Wen 2014 | 42 | / | 50 | 20-69 | 90 | / | / | / | / | / | / |
| Peereboom 2010 | 28 | / | 53 | 18-84 | / | 89.9 | 69.1 | 30 | 14.3 | / | / |
| Brown 2008 | 97 | / | 57 | 30-71 | 90 |  | 57 | 35 | 44 | / | / |
| Prados 2009 | 66 | IG=66 CG=128 | IG=55 CG=53 | IG=22-77 CG=27-77 | / | 90 | / | IG=39 CG=30 | IG=24.2 | / | / |
| Krishnan 2006 | 20 | / | 54 | 32-81 | / | / | 73.7 | 36.8 | / | / | / |
| Chakravarti 2013 | 147 | IG=147 CG=1457 | / | / | / | / | / | 26 | / | / | / |
| Kreisl 2008 | 22 | / | 59 | 22-72 | 80 | / | 50 | / | / | 14 | / |
| Rich 2004 | 57 | / | 54 | 19-74 | 90 | 100 | 63 | 21 | / | 0 | 37.4w |
| Uhm 2011 | 96 |  | 57 | 19-79 | / | / | 67 | 47 | / | / | / |
| Reardon 2015 | 119 | CG= 39 IG1=41 IG2=39 | CG= 56.9 IG1= 56.6 IG2= 55.4 average | / | CG= 80 IG1= 80  IG2= 80 | / | CG= 64.1 IG1= 65.9 IG2=53.8 | / | / | CG= 10.4 IG1= 2.4 IG2=7.7 | CG= 9.2m IG1= 11.7m IG2=11.0m |
| Chi 2020 | 30 | / | 61 | 42-77 | / | 97 | 50 | / | 48 | / | 11.1m |
| Sepulveda-Sanchez 2017 | 49 | IG1 (EGFR amp/EGFRvIII-)=30 IG2 (EGFR amp/EGFRvIII+)=19 | IG1=62 IG2=52 | IG1=41-81 IG2=39-72 | / | / | IG1=66.7 IG2=63.2 | / | IG1=30% IG2=5.3% | IG1=6.6 IG2=5.3 | / |
| Solomon 2013 | 73 | IG=14 CG=15 | IG=44 CG=46.5 | / | IG=90 CG=80 | IG=84.3 CG=81.5 | IG=65.6 CG=50 | IG=17.2 CG=5.4 | / | / | / |
| Solomon 2014 | 19 | / | / | / | / | 54.3 | / | / | / | / | / |
| Westphal 2015 | 142 | IG=71 CG=71 | IG= 55 CG=56 | IG=25-71 CG=30-70 | IG=90.4 CG=88.9 | / | IG=59.2 CG=63.4 | IG=43.7 CG=42.3 | IG=21.1 CG=22.5 | / | / |
| Wang 2016 | 26 | / | 50.5 | 18-76 | 75 | / | 80.8 | 34.6 | / | / | / |
| Du 2019 | 36 | / | 49 | / | / | / | 52.8 | 41.7 | 55.6 | 72.2 | / |
| Ramos 2006 | 16 | GBM=16 AA=12 anaplastic oligodendroglioma =1 | 45 | 20-68 | 80 | / | / | / | / | 31.3 | / |
| Desjardins 2017 | 24 | / | / | / | / | / | / | / | / | / | / |
| Brady 1992 | 15 | / | 54 | 27-68 | 80 | / | 40 | 13.3 | / | / | / |
| Li 2010 | 192 | IG1=132 IG2=60 CG=81 | IG=53 CG=66 | IG1=17-78 CG=25-84 | / | / | IG1=56.8 CG=49.4 | / | / | / | / |
| Spiekman 2023 | 27 | / | 54.5 | 35-73 | / | / | 70 | / | 11 | 8.3 | 14m |
| Choi 2023 | 21 | / | 57 | 37-71 | 70 | / | 52.4 | / | 38.1 | 5.6 | / |
| Gan 2018 | 38 | IG1: 29 (14 primary, 15 recurrent)  IG2: 9 (all recurrent) | G1 primary =52 IG1 recurrent = 50 IG2= 59 | IG1 primary =36-78 IG1 recurrent = 20-71 IG2= 44-76 | 90 | / | 47.4 | / | / | 7.9 | / |
| Lassman 2019 | 60 | / | 56 | 20-79 | 90 | / | 58 | / | / | 13.8 | / |
| Caccese 2020 | 57 | / | 57 | 37-73 | / | / | 69 | / | 64 | / | / |
| Reardon 2013 | 11 | / | 60 | 39-69 | / | / | 81.2 | / | / | / | / |
| Van den Bent 2020 | 260 | IG1=88 IG2=86 CG=86 | IG1=59.2 IG2=58.3 CG=58.8 | IG1=40.1-75.4 IG2=36.3-79.3 CG=34.9-82.3 | / | / | IG1=67 IG2=58.1 CG=67.4 | / | IG1=48.9 IG2=47.7 CG=48.8 | / | IG1=6w IG2=5.81w CG=6.23w |
| Lassman 2023 | 639 | IG=323 CG=316 | IG=59 CG=60 | IG=22-84 CG=29-82 | IG=90 CG=90 | / | 62 | IG=57 CG=57 | IG=37 CG=37 | / | / |
| Schuster 2015 | 65 | / | 56 | 30-83 | 90 | / | 51 | / | 38 |  | 3m |
| Sampson 2010 | 18 | IG=18 Matched cohort/CG=17 | IG= 52 CG=59 | IG=29-67 CG=37-71 | IG=90 CG=90 | / | IG=72 CG=47.1 | / | 44.4 | / | / |
| Weller 2017 | 745 | IG=371 CG=374 | IG=59 CG=58 | IG=51-64 CG=52-64 | / | / | IG=68 CG=61 | / | IG=33 CG=35 | / | IG=2.9m CG=2.8m |
| Goff 2019 | 18 |  | 57 | 43-66 | / | / | 83.3 | / | 22.2 | 0 | / |
| Bagley 2024 | 8 | / | 67.5 | 67-76 | / | / | 75 | / | 0 | / | / |
| Gerstner 2015 | 45 | IG1=20 prior anti-VEGF IG2=20 anti-VEGF naïve | 54 | 22.5-80.4 | 80 | / | 73 | / | / | 8.9 | / |
| Batchelor 2010 | 31 | / | 53 | 20-77 | / | / | 58.1 | / | / | / | / |
| Batchelor 2013 | 325 | IG1=131 IG2=129 CG=65 | IG1=54 IG2=54 CG=54 | / | / | / | / | / | / | IG1=15.3 IG2=17.2 CG=8.9 | / |
| Batchelor 2023 | 158 | IG= 103 CG=55 | 60 | 27-83 | / | 94.6 | 54.4 | 59.1 | 36.2 | / | / |
| Hsieh 2022 | 70 | IG=35 CG=35 | / | / | / | / | / | / | / | / | / |
| Duerinck 2016 | 44 | IG=22 CG=22 | IG=53 CG=56 | IG=31-79 CG=20-71 | / | IG=100 CG=95 | IG=58 CG=77 | / | / | IG=27 CG=23 | / |
| Duerinck 2018 | 79 | IG=31 CG=50 | IG=56 CG=55 | IG=28-75 CG=27-79 | / | / | / | / | IG=25 CG=33 | / | / |
| Awada 2020 | 54 | IG1=27 IG2=27 | 55 | 19-75 | / | 63 | / | / | / | 27.8 | / |
| Ge 2020 | 31 | / | 53 | 21-70 | / | / | 64.5 | / | 16.1 | 26.3 | / |
| Wang 2019 | 20 | / | 50.5 | 27-67 | 70 | / | 65 | / | / | 45 | / |
| Zhang 2018 | 33 | / | / | / | / | / | / | / | / | 25 | / |
| Zhang 2022 | 19 | / | 47 | 19-63 | / | / | 47.4 | 47.4 | 42.1 | 52.6 | 14m |
| Schiff 2015 | 66 | IG1 (CT-322 1mg/kg) = 14 IG2 (CT-322 1mg/kg+ irinotecan)=7 IG3 (CT-322 2mg/kg)=27 IG4 (CT-322 2mg/kg + irinotecan)=16 | / | / | / | / | 62.5 | / | / | IG1 = 14.3 IG2n=0 IG3=3.8 IG4=0 | / |
| Kalpathy-Cramer 2017 | 10 |  | 62 | 51-74 | 90 | / | 40 | / | 10 | 20 | / |
| Baumann 2004 | 44 | IG1=19 IG2=25 | 54 | 26-72 | / | / | 54 | / | / | / | 16 w |
| Chang 2004 | 67 | / | 51 | 27-77 | 90 | / | 58 | 31 | / | / | / |
| Marx 2001 | 42 | / | 55 | 27-77 | 80 | / | 61.9 | / | / | 5 | 32.7w |
| Fine 2003 | 38 | / | 52 | 28-71 | 80 | / | 42.5 | / | / | / | 47w |
| Fadul 2008 | 26 | ND=10 R=16 | ND=58 R=54 | ND=10 R=16 | 80 | / | / | ND=20 R=37.5 | / | / | ND=95 days R=288 days |
| Riva 2007 | 46 | IG=23 CG=23 | IG= 59 CG= 54 | IG=28-70 CG=32-66 | / | / | IG=52.1 CG=65.2 | IG=21 CG=26 | / | / | / |
| Groves 2007 | 44 | / | 53 | 32-84 | 80 | / | 34.9 | 55 | / | 7 | / |
| Kesari 2008 | 50 | / | 54 | 29-78 | 90 | / | 64 | / | / | 0 | / |
| Alexander 2013 | 89 | / |  | / | / | / | 62 | 25 | / | / | / |
| Penas-Prados 2015 | 178 | TMZ+ Thal=18 TMZ+CRA+Thal=17 TMZ+Cel+Thal=20 TMZ+CRA+Cel+Thal=15 | 53 | 18-84 | / | 89.9 | 69.1 | 55.6 | / | / | 3.4m |
| Groot 2011 | 42 | / | 55 | 33-72 | 90 | / | 40.7 | / | / | 18 | / |
| NCT00895180 2017 | 80 | IG1=40 IG2=40 | / | / | / | IG1=95 IG2=92.5 | IG1= IG2= | / | / | IG1=0 IG2=2.5 | / |
| Nabors 2010 | 24 | / | 55 | 20-73 | 90 | 87 | 61 | / | / | / | / |
| Brown 2016 | 38 | IG=19 CG=19 | 57 | 30-71 | 90 | / | 71.1 | / | / | / | 12.1m |
| D'alessandris 2013 | 10 | 4 pts with EGFRvIII expression received erlotinib + bev  6 pts without EGFRvIII only received bev | 52.75 | 30-68 | / | / | 100 |  | / | 100 | / |
| D'alessandris 2019 | 34 | bev + erlotinib = 14 bev only = 16 bev + sirolimus = 4 | / | / | / | / | / | / | / | / | / |
| Clarke 2014 | 59 | / | 54 | 21-76 | 90 | / | / | 33 | / | / | / |
| Sathornsumetee 2010 | 25 | / | 52.4 | 24.1-70.4 | / | / | 52 | / | / | 48 | 34w |
| Cardona 2021 | 15 | / | 56 | 38-70 | / | / | 66.7 | 53.3 | 46.7 | 13.3 | / |
| Wu 2025 | 18 | / | / | 19-75 | 80 | 85 | 44.4 | 50 | 44.4 |  | / |
| Hasselbalch 2010 | 43 | / | 54 | 23-70 | / | / | 58 | / | / | 26 | 266 days |
| Reardon 2020 | 73 | IG=36 CG=37 | IG= 36 CG=37 | IG=44-79 CG=30-75 | IG=80 CG-80 | / | IG=53 CG=59 | IG=39% CG=16% | / | IG=30% CG=18% | / |
| Phuphanich 2017 | 56 | / | 56.5 | 23-79 | / | 89.1 | 66.1 | / | / | 0 | / |
| Andrews 2021 | 68 | IG: 33  CG: 35 | 63 | 32-77 | / | / | 60.6 | 30.3 | 48.5 | / | / |
| Affronti 2018 | 36 | / | 55.5 | 27-74 | / | / | 61.1 | / | / | 27.8 | / |
| Wen 2011 | 60 | IG1=40 IG2=20 | 54 | 19-71 | 80 | 90 | 63 | / | / | IG1=0 IG2=0 | 16m |
| Cloughesy 2017 | 129 | IG=64 CG=65 | IG=57 CG=55 | / | / | IG=92.2 CG=98.5 | IG=68.8 CG=60 | IG=50 CG=58.5 | IG=37.5 CG=48.1 | IG=22.2 CG=23.7 | / |
| Lombardi 2019 | 119 | IG=59 CG=60 | IG=54.8 CG=58.9 | IG=46.8-61.3 CG=51.8-65.2 | / | / | IG=69 CG=72 | / | IG=49 CG=46 | IG=5 CG=3 | IG=10.7m CG=9.8m |
| Lombardi 2021 | 54 | / | 54 | 30-77 | / | / | 70.4 | 52 | 52.8 | 7.4 | 14.3m |
| Caccesse 2024 | 190 |  | 58.5 | / | / | / | 68 | / | 49.7 | 7.3 | / |
| Tunbekici 2024 | 65 | / | 53 | 18-67 | / | / | 60 | 63.1 | 1.3 | 4.6 | / |
| Mazzarella 2022 | 41 | / | 54 | 26-76 | / | / | 48.8 | / | 46.3 | / | / |
| Lai 2024 | 33 | / | 52 | 32-69 | 90 | / | 52 | / | 33 | / | / |
| Wang 2024 | 17 | / | 49 | 16-75 | 70 | / | 76.4 | / | 29.4 | 29.4 | / |
| Yang 2021 | 21 | / | 49 | 16-75 | 60 | / | 61.3 | / | 38.7 | 33.3 | / |
| Guan 2022 | 5 | / | 51 | 43-60 | / | / | 60 | / | 100 | 100 | 10.4m |
| Cao 2021 | 30 | / | / | / | / | / | / | / | / | 76.7 | / |
| She 2021 | 20 | / | 52 | 18-71 | 80 | / | 50 | 90 | 29.2 | 28 | / |
| Yang 2022 | 25 | / | 49 | 23-62 | 80 | / | / | / | 29.2 | 28 | / |
| Zhao 2024 | 21 | IG1= 14 IG2= 7 | / | 35-67 | / | / | 72 | / | / | 33 | / |
| Hainsworth 2010 | 47 | / | 54 | 28-79 | / | / | 66 | / | / | 13 | / |
| Hassler 2014 | 24 | / | 47.5 | 31-71 | / | / | 56.3 | / | / | 10 | 21m |
| Zustovich 2013 | 43 | / | 60 | 36.1-77 | / | / | / | / | / | / | / |
| Galanis 2013 | 54 | / | 55 | 25-76 | / | / | / | / | / | 18.5 | / |
| Schiff 2018 | 115 | IG1=12 IG2=49 IG3=9 IG4=45 | / | / | / | / | / | 52.2 | / | 9 | / |
| Peereboom 2013 | 56 | / | 56 | 31-78 | 80 | / | / | / | / | 5 | / |
| Chen 2020 | 19 | 19 | 52 | 30-76 | 90 | / | 47.4 | / | / | 0 | / |
| Dresemann 2010 | 240 | IG=120 CG=120 | IG=52 CG=51 | 19-73 | / | / | IG=58 CG=68 | / | / | / | 12m |
| Reardon 2005 | 33 | / | 52 | 23-68 | 90 | / | 72 | / | / | 9 | 37.7w |
| Wen 2006 | 34 | / | 52 | 27-73 | 80 | / | 55 | / | / | 6 | / |
| Raymond 2008 | 112 | GBM=51 | / | 19-72 | / | / | 58 | / | 6 | / | / |
| Hassler 2014 | 24 | 53 | 18-72 | / | 55 | / | 41.5 | / | / | 8.3 | 10.5m |
| Razis 2009 | 20 | / | / | / | / | / | 70 | 5 | / | / | / |
| Sautter 2020 | 51 | IG1=19 IG2=32 | IG1=63 IG2=52.5 | IG1=35-78 IG2=29-70 | / | / | / | 0 | / | / | / |
| Reardon 2009 | 231 | / | 56 | 18-80 | / | / | 62.3 | / | 10.8 | 3.4 | 9m |
| Kreisl 2012 | 63 | IG1= 61; IG2 = 62 | / | / | / | / | / | / | / | 4.7 | / |
| Dresemann 2005 | 30 | / | 44 | 16-71 | / | / | 83.3 | 43.3 | / | 20 | 12m |
| Balana 2014 | 12 | / | 65 | 48079 | / | / | 58.3 | / | / | 0 | 24w |
| Pan 2012 | 16 | / | 57.8 | 44-77 | / | 87.5 | 62.5 | 87.5 | / | / | / |
| Hutterer 2014 | 40 | / | 58 | 36-79 | / | / | 72 | 37 | 27.5 | 0 | 9m |
| Janssen 2024 | 55 | IG1=12 CG1=14 IG2=14 CG2=15 | IG1=50 CG1=53 IG2=60 CG2=56 | IG1=34-63 CG1=30-71 IG2=40-77 CG2=39-68 | / | / | IG1=92 CG1=64 IG2=64 CG2=67 | / | IG1=42 CG1=43 IG2=43 CG2=20 | IG1=8 CG1=7 IG2=0 CG2=0 | / |
| Faye 2023 | 37 | / | 50 | 30-76 | 95 |  | 68.6 | 54.3 | 0 | / | / |
| Martinez-Garcia 2022 | 38 | / | 52 | 33.3-76.5 | 90 | / | 44.4 | / | 44.4 | 28.6 | / |
| Sharma 2019 | 33 | IG1=19 IG2=14 | 57 | 26-68 | 80 | / | 64 | 76 | / | 6 | IG1=8.9m IG2=26.9m |
| Lee 2019 | 15 | / | 61 | 27-74 | 80 | 73.3 | 86.7 | / | 0 | / | / |
| NCT01137604 2022 | 80 | IG=42 CG=38 |  |  | / |  | IG=69 CG=65.8 | / | / | IG=21.4 CG=15.8 | / |
| Rha 2025 | 101 |  | 56 | 19-77 | / | 72 | 63 |  | 39 | 20 | / |
| Reardon 2013 | 41 | IG1=19 IG2=22 | 50 | 20-76 | / | / | 75.6 | / | / | IG1=0 IG2=5 | 11.9m |
| Iwamoto 2010 | 35 | / | 53 | 29-73 | 95 | / | 63 | / | / | / | 5.9m |
| Lee 2015 | 114 | IG=70 CG=36 | IG=59 CG=55 | IG=23-83 CG=23-73 | IG=90 CG=90 | IG=91.4 CG=88.9 | IG=62.9 CG=47.2 | IG=56.7 CG=34.5 | / | IG=7.8 CG=17.6 | / |
| McNeill 2014 | 64 | IG=32 CG=32 | / | / | / | / | / | / | / | / | / |
| Kreisl 2012 | 32 (GBM) | / | 48 | 21-70 | 90 | / | 63 | / | / | 12.5 | / |
| Chheda 2015 | 22 | / | 52.5 | 33-71 | 90 | / | 64 | / | / | / | / |
| Franceschi 2012 | 26 | / | 54.8 | 38.8-70.7 | / | / | 76.9 | / | / | / | 1.9m |
| Breen 2025 | 204 | IG=138 CG=66 | IG=60 CG=59.5 | 29-82 | / | 94 | 62.7 | 14.2 | 18.1 | / | / |
| Lu-Emerson 2011 | 14 | / | 55 | 32-66 | 80 | / | / | / | / | / | / |
| Lassman 2015 | 50 | IG1=21 IG2=29 | IG1=51 IG2=51 | IG1=33-81 IG2=26-75 | / | IG1=95 IG2=90 | IG1=48 IG2=59 | IG1=43 IG2=62 | / | / | / |
| Muhic 2013 | 25 | IG1=13 IG2=12 | IG1=55 IG2=52 | IG1=33-72 IG2=31-68 | / | / | IG1=54 IG2=67 | / | / | 0 | / |
| Norden 2015 | 35 | IG1=22 IG2=14 | IG1=54 IG2=52 | IG1=28-75 IG2=32-70 | IG1=90 IG2=90 | / | IG1=31.8 IG2=78.5 | / | / | 0 | / |
| Wen 2018 | 70 | IG1=12 IG2=58 | 52.5 | 23-71 | / | IG1=91.7 IG2=93.1 | 61.4 | / | / | IG1=8.3 IG2=3.4 | IG1=1.07 years IG2=1.16 years |
| Odia 2016 | 41 | / | 55 | 26-75 | 90 | / | 72 | / | / | / | / |
| Batchelor 2017 | 30 | / | 54 | 24-69 | / | 77 | / | / | / | / | / |

**Supplementary Table 3.** Personalised therapies and survival reported in included studies. IG = intervention group, CG = control group, RT = radiotherapy, CT = chemotherapy, TMZ = temozolomide, SC = subcutaneous, IV = intravenous, ATL = autologous tumour lysate, TTP = time to progression, OS = overall survival, PFS = progression free survival.

| Study | RTK drug | RTK target | Therapy details | Median PFS | Median OS | Additional survival outcomes | Interpretation |
| --- | --- | --- | --- | --- | --- | --- | --- |
| van den Bent 2009 | Erlotinib | EGFR | IG=Erlotinib 150 mg with dose escalation to 200 mg daily  CG= TMZ or carmustine | IG=1.8m CG=2.4m | IG=7.7m CG=7.3m (HR 1.14, p=0.91) | / | No survival benefit observed |
| Raizer 2009 | Erlotinib | EGFR | Erlotinib 150mg daily | rGBM=2m | rGBM=6m | GBM OS-12=63%, PFS-12=9% | Appears unbeneficial |
| Yung 2010 | Erlotinib | EGFR | Erlotinib 150 or 300 mg/day orally continuously | IG1 = 8.3m IG2 =10.6m | IG1 = 21.7% IG2 = 18.3% | / | Appears unbeneficial |
| Gallego 2014 | Erlotinib | EGFR | Erlotinib 150mg daily | 3.9m | 7m | PFS-6=20% | Appears unbeneficial |
| de Groot 2008 | Erlotinib | EGFR | Carboplatin on day 1 of 28-day cycle Erlotinib 150mg per day, increased in 25mg every cycle to max dose of 200 mg per day as tolerated | 9w | 6.9m | PFS-6=14% | Appears unbeneficial |
| Wen 2014 | Erlotinib | EGFR | Erlotinib 150 mg (escalated to 200mg) and MTD temsirolimus | 8 weeks | / | PFS-6=13% | Appears unbeneficial |
| Peereboom 2010 | Erlotinib | EGFR | Erlotinib 50 mg /day with escalation by 50 mg/day every 2 weeks + RT 2 Gy/day 5 days per week + TMZ 75 mg/m2/day for 42 days | 2.8m | 8.6m | PFS-6=30% | Appears unbeneficial |
| Brown 2008 | Erlotinib | EGFR | Erlotinib 150mg daily alone for 1 week, then concurrently with tmz (75 mg mg/2 daily) and RT (60gy),concurrently with up to six cycles of adjuvant TMZ (200 mg/m2 daily for 5 days every 28 days) | 7.2m | 15.3m | OS-12 61% | Appears unbeneficial |
| Prados 2009 | Erlotinib | EGFR | IG: erlotinib 100-200mg/d during XRT and 150-300mg/d after XRTof erlotinib  XRT 1.8/2.0 Gy 5 days per wekk to a total of 59.4-60Gy over 6-week course TMZ 75 mg/m2/d continuous 7 days /week  CG = TMZ + thalidomide or isotretinoin | IG= 8.2m CG=4.9m (PFS HR 0.56, p=0.001) | IG=19.3m CG=14.1m (OS HR 0.64, p=0.01) | / | Survival benefit observed |
| Krishnan 2006 | Erlotinib | EGFR | Erlotinib 100 mg/day and increments of 50 mg/day; radiation therapy was delivered once a day, 5 times a week, in standard fractions of 200 cGy. | IG1=27w IG2=23w | IG1= 11.7m IG2= 15.2m | / | Appears unbeneficial |
| Chakravarti 2013 | Gefitinib | EGFR | Gefinitib 500mg + cranial radiotherapy continued post RT for 18 months or until progression | 4.9m | IG=11.5m CG=11.0m (p=0.91) | PFS-6= 40% | No survival benefit |
| Kreisl 2008 | Gefitinib | EGFR | Gefitinib 250 mg daily + everolimus | 2.6m | 5.8m | / | Appears unbeneficial |
| Rich 2004 | Gefitinib | EGFR | Gefinitib 500-1000 mg/d orally | 3.9m | 9.1m | PFS-6=13.2% OS-12=35.6% | Appears unbeneficial |
| Uhm 2011 | Gefitinib | EGFR | Gefitinib 500 mg/day escalated to 1000mg/day | / | / | OS-12=54.2% (historical control of 48.9%) p=0.25 PFS-12=16.7% (historical control 30.3%) =0.05 | Appears unbeneficial |
| Reardon 2015 | Afatinib | EGFR | CG TMZ monotherapy IG1: afatinib monotherapy at 40mg/day IG2: afatinib + TMZ | CG=1.87m IG1=0.99m p=0.032 IG2=1.53 p=0.204 | CG= 10.6m IG1= 9.8m p=0.386 IG2=8 p=0.119 | / | No survival benefit associated |
| Chi 2020 | Dacomitinib | EGFR | Dacomitinib 45mg once daily during a 28-day cycle | 2.04m | 9.9m | PFS-6=30% | Appears unbeneficial |
| Sepulveda-Sanchez 2017 | Dacomitinib | EGFR | Dacomitinib 45 mg/day orally | IG1=2.7m IG2=2.6m | IG1=7.8m IG2=6.7m | IG1 PFS-6=13.3% IG2 PFs-6=5.9% | Appears unbeneficial |
| Solomon 2013 | Nimotuzumab | EGFR | Nimotuzumab IV or placebo weekly for 6 weeks + concurrent RT 180-200 cGy 5 days per week to a total of 5000-6000 cGy | / | IG=8.4m CG=8.36m | / | Appears unbeneficial |
| Solomon 2014 | Nimotuzumab | EGFR | Nimotuzumab administered weekly for 6 weeks, together with radiotherapy, and then treatment continued every 21 days until 1 year. | / | 11.8m | / | Appears unbeneficial |
| Westphal 2015 | Nimotuzumab | EGFR | IG= standard radiation with 60 Gy in 30 fraction with concomitant TMZ (75 mg/m2 daily) followed by six cyclces of adjucant TMZ therapy (150 mg/m2) + IV 400 mg nimotuzumab every week for 12 weeks then every 2 weeks  CG= standard radiochemotherapy alone | IG=7.7m CG=5.8m (p=0.7898) | IG=22.3m  CG=19.6m (p=0.4856) | PFS-12 IG 22%; CG=18% (p=0.5326) | No survival benefit associated |
| Wang 2016 | Nimotuzumab | EGFR | RT 2 Gy/d 5 days per week to a total of 60 Gy over 6 weeks + TMZ 75 mg/m2/day for 40-42 days + Nimotuzumab 200 mg IV per week for 6 weeks | 10m | 15.9m | PFS-6=69.2% OS-6=88.5% OS-12=69.2% OS-24=19.2% | Appears beneficial |
| Du 2019 | Nimotuzumab | EGFR | Nimotuzumab 200mg once weekly for 6 weeks with RT and concomitant and adjuvant temozolomide + individualised adjuvant TMZ based on MGMT expression status | 11.9m | 24.5m | PFS-12: 49.3%; OS-12 83.3% | Appears beneficial |
| Ramos 2006 | h-R3 | EGFR | 6 weekly infusion of h-R3 200mg + RT | / | 17.47m | OS-12=75% | Appears beneficial |
| Desjardins 2017 | Sym004 | EGFR | Sym004 18mg/kg IV every 2 weeks | / | / | 12.5% PFS-6 | Appears unbeneficial |
| Brady 1992 | Mab-425 | EGFR | Iodine-125 labelled Mab-425 after surgical debulking/biopsy and 3-4 weeks radiation therapy for a total of 40-224mCi | / | 15.6m | OS-12=60% | Appears beneficial |
| Li 2010 | Mab-425 | EGFR | Adjuvant 125I-425 mAb as 3 weekly intravenous injections of 50 mCi (1.8 MBq), totaling approximately 150 mCi (5.4 MBq) 4–6 weeks after completion of surgery and radiation therapy IG1= mAb alone IG2= mAb+ TMZ | / | IG1=14.5m IG2=20.4m CG=7.2m | / | Survival benefit observed |
| Spiekman 2023 | Panitumumab | EGFR | 6 mg/kg Panitumumab intravenously (iv) every 2 weeks | 1.7m | 4.5m | / | Appears unbeneficial |
| Choi 2023 | GC1118 | EGFR | GC1118 on Days 1, 8, 15, and 22 of a 28‐day cycle for 6 cycles | 1.7m | 5.7m | PFS6=5.6% | Appears unbeneficial |
| Gan 2018 | Depatuximab | EGFR | RT 2 Gy/d 5 days per week to a total of 60 Gy over 6 weeks + TMZ 75 mg/m2/day for 40-42 days + Nimotuzumab 200 mg IV per week for 6 weeks | IG1 recurrent = 2.3m  IG1 primary = 3.7m IG2 = 2.3m | IG1 recurrent = 10.7m IG1 primary = 17.9m IG2 = 7.2m | PFS-6 IG1 recurrent = 30.8% IG1 primary = 26.7% IG2 = 40% | Appears beneficial |
| Lassman 2019 | Depatuximab | EGFR | Depatux-m (0.5-1.5 mg/kg) on days 1 and 15 + TMZ | 2.1m | 7.4m | PFS-6=25.25% OS-6=69.1% | Appears unbeneficial |
| Caccese 2020 | Depatuximab | EGFR | Depatuxizumab Mafodotin 1.25 mg/kg every 2 weeks + TMZ | 2.3m | 8.04m | OS-12 37%, PFS-6 38% | Appears unbeneficial |
| Reardon 2013 | Depatuximab | EGFR | ABT-806 24 mg/kg IV every other week | 29 days | / | / | Appears unbeneficial |
| Van den Bent 2020 | Depatuximab | EGFR | Depatux-M 1.25 mg/kg IV every 2 weeks with TMZ 150-200 mg/m2 day 1-5 in 28 day cycles (IG1) OR monotherapy with depatux-M (IG2) OR lomustine/TMZ (CG) | IG1=2.7m IG2=1.9m CG=1.9m | IG1=9.6m IG2=7.9m CG=8.2m (IG1 vs CG HR=0.66, p=0.017; IG2 vs CG HR=0.96, p=0.8) | OS-12 IG1= 39.7% IG2=26.7% CG=28.2% | Survival benefit observed |
| Lassman 2023 | Depatuximab | EGFR | Depatux-M 2mg/kg during RT followed by 1.25 mg/kg TMZ on days 1 and 15/28 OR placebo + RT + TMZ | IG=8m CG=6.3m (HR 0.84 p=0.029) | IG=18.9m CG=18.7m (HR 1.02, p=0.63) | / | No survival benefit associated |
| Schuster 2015 | Rindopepimut | EGFR | Rindopepimut was administered in an initial vaccine priming phase (days 0, 14, and 28), and then monthly, concurrent with standard adjuvant temozolomide chemotherapy (targeted dose of 200 mg/m2; days 1–5 of repeated 28-day cycles) | 9.2m | 21.8m | PFS5.5=66% (p=0.0168) | Appears beneficial |
| Sampson 2010 | Rindopepimut/PEPvIII-KLH | EGFR | Vaccination with PEPvIII-KLH every two weeks starting 4 weeks after RT completion for first 3 doses, subsequent doses once monthly until radiographic evidence of tumour progression | IG=14.2m CG=6.3m (p=0.013) | IG=26m CG=15m (p=0.002) | PFS-6=67% OS-6=94% | Survival benefit observed |
| Weller 2017 | Rindopepimut | EGFR | Maintenance TMZ orally 150-200 mg/m2 on days 1-5 of each 28-day cycle + Rindopepimut 500ug with 150ug GM-CSF or placebo 100ug haemocyanin (on days 1 and 15 then monthly on day 22 of each TMZ cycle) | IG=7.1m CG=5.6m (HR 0.94, p=0.51) | IG=17.4m CG= 17.4m (HR0.89, p=0.22) | / | No survival benefit associated |
| Goff 2019 | anti-EGFRvIII CAR-T | EGFR | 2 days of cyclophosphamide (60 mg/kg) followed by five days of fludarabine, autologous CAR-transduced cells were infused the next day | 1.3m | 6.9m | / | Appears unbenficial |
| Bagley 2024 | anti-EGFRvIII CAR-T | EGFR | hypofractionated course of radiation (40 Gy, 15 fractions), afterwards a 200-mg pembrolizumab infusion followed 1 h later by CAR T cells (2 × 108 cells, 10–20 ml min−1) | 5.2m | 11.8m | / | Appears unbeneficial |
| Gerstner 2015 | Cediranib | VEGFR | Cediranib 30mg daily + cilengitide 2000mg IV twice weekly | IG1=1.9m IG2=1.9m | IG1=6.3m IG2=6.5m | / | Appears unbeneficial |
| Batchelor 2010 | Cediranib | VEGFR | Cediranib monotherapy 45 mg/day until progression or unacceptable toxicity | 3.85m | 7.46m | APF6 25.8% | Appears unbeneficial |
| Batchelor 2013 | Cediranib | VEGFR | Cediranib monotherapy (30mg) (IG1) OR Cediranib (20mg) plus lomustine (110mg/m2) (IG2) OR Placebo plus lomustine (110mg/m2) | IG1=3.02m (HR 1.05, p=0.9) IG2=4.11m (HR0.76, p=0.16) CG=2.14m | IG1=8m (HR1.43, p=0.1) IG2=9.4m (HR1.15m p=0.5) CG=9.8m | APF6 IG1=16% IG2=35% CG=25% | No survival benefit associated |
| Batchelor 2023 | Cediranib | VEGFR | Cediranib (20 mg) versus placebo in combination with temozolomide (75 mg/m2/daily) and radiation (60 Gy in 2 Gy fractions) daily for 42 days followed by cediranib (20 mg) versus placebo in combination with temozolomide (150–200 mg/m2 for 5 consecutive days in 28-day cycles) for up 12 cycles maximum | IG=6.25m CG=2.7m (HR 0.67, p=0.03) | IG=14.5m CG=13.8m (HR=0.87, p=0.44) | PFS6 IG=46.6% CG=24.5% (p=0.005) | No survival benefit associated |
| Hsieh 2022 | Cediranib | VEGFR | Cediranib (30mg PO daily)/olaparib (200mg PO twice daily) or bevacizumab (10mg/kg IV every 2 weeks) | IG=3.87m CG=3.02m (HR 1.099, 95% CI 0.6-2, p = 0.76) | IG=8.86m CG=6.31m (HR 0.6892, 95% CI 0.39-1.2, p = 0.2) | / | No survival benefit associated |
| Duerinck 2016 | Axitinib | VEGFR | Axitinib 5mg twice daily (increased to 7-10 mg BID) monotherapy or bev with lomustine (physician's best treatment) | IG=13w CG=10w (p=0.28) | IG=29w CG=17w (p=0.681) | / | No survival benefit associated |
| Duerinck 2018 | Axitinib | VEGFR | Axitinib 5mg twice daily (increased to 7-10 mg BID) monotherapy or combined with lomustine | IG=13w CG=12.4 | IG=27.3w CG=29w | PFS6: CG=26%; IG =24.1% OS-6: CG=54%; IG=53% | No survival benefit associated |
| Awada 2020 | Axitinib | VEGFR | Axitinib 5 mg oral BID + avelumab 10 mg/kg IV Q2W (cohort 1), AVE added after 6 weeks (cohort 2) | IG1=12w IG2=10.7w | IG1=26.62w IG2=18w | OS-12 IG1=22.2% IG2=11.1% PFS6 IG1=22.2 IG2=18.5% | Appears unbeneficial |
| Ge 2020 | Apatinib | VEGFR | Apatinib 500g once daily + TMZ | 4.9m | 8.2m | PFS-6: 44.6% 12m-OS: 30.2% | Appears unbeneficial |
| Wang 2019 | Apatinib | VEGFR | Apatinib 500mg orally once daily + TMZ (7 on 7 off) in 28 day cycles | 6m | 9m | / | Appears unbeneficial |
| Zhang 2018 | Apatinib | VEGFR | Apatinib 500 mg daily + TMZ 200 mg/m2/d 5/28 days, after 12 cycles, apatinib taken as maintenance until progression | 4.9m | 8.3m | PFS-6 GBM=47.8%  OS-12 GBM=36.2% | Appears unbeneficial |
| Zhang 2022 | Apatinib | VEGFR | Apatinib 500 mg/day | 5.1m | 10.4m | PFS-6=38.9% OS-6=68.4% OS-12=36.8% | Appears unbeneficial |
| Schiff 2015 | CT-322/pegdinetanic | VEGFR | CT-322 1-2 mg/kg monotherapy or + irinotecan 125 or 340 mg/m2 intravenously every 2 weeks depending on EIAED usage | IG1 = 1.4m IG2 = 14m IG3 = 1.8m IG4 = 4.1m | / | PFS6 CT-322 monotherapy = 18.6%  PFS6 CT-322 and irinotecan=0% | Appears unbeneficial |
| Kalpathy-Cramer 2017 | Tivozanib | VEGFR | Tivozanib oral 1.5mg daily for first 4 out of 5 week cycle until progression or unacceptable toxicity | 2.3m | 8.1m | PFS6=10% | Appears unbeneficial |
| Baumann 2004 | Thalidomide | VEGFR | Thalidomide, thalidomide + temozolomide (after microsurgical tumour extirpation and radiotherapy) | IG1= 17w  IG2= 36w (0=0.06) | IG1=14.5 IG2=23.7m (p=0.01) | / | Appears beneficial |
| Chang 2004 | Thalidomide | VEGFR | Thalidomide 200mg and escalated by 100-200mg every 1-2 weeks up to max of 1200 mg daily + TMZ + RT | 5.06m | 16.8m | 45% PFS-6, 29% OS-12 | Appears unbeneficial |
| Marx 2001 | Thalidomide | VEGFR | 100 mg/day thalidomide, increased at weekly intervals by 100 mg to a maximum tolerated dose of 500 mg/d | 2.5m | 7.1m | OS-12=35% | Appears unbeneficial |
| Fine 2003 | Thalidomide | VEGFR | Thalidomide 800mg/d for the first 2 weeks, escalated by 200 mg every 2 weeks up to 1200mg/d + BCNU IV 1 hour 200 mg/m2 every 6 weeks | 3.4m | / | PFS6=27.03% | Appears unbeneficial |
| Fadul 2008 | Thalidomide | VEGFR | Thalidomide 100 mg/day + irinotecan 350 or 700 mg/m2 | ND=4.6m | All=10.2m | PFS6 All=27%; Primary=40%; Recurrent=22% | Appears unbeneficial |
| Riva 2007 | Thalidomide | VEGFR | 60 Gy LINAC RT 5 weeks after surgery  TMZ monotherapy or combined with thalidomide (started day 7 after RT with 100 mg daily increased to a final dose of 600-1200 mg) | / | IG=13m CG=12m | / | No survival benefit associated |
| Groves 2007 | Thalidomide | VEGFR | Thalidomide 400 mg orally at bedtime for 28 consecutive days, TMZ 150–200 mg/m2 orally once a day for five consecutive days every 28 days | 15 weeks | / | PFS6 = 23% | Appears unbeneficial |
| Kesari 2008 | Thalidomide | VEGFR | standard radiation therapy, temozolomide for 5 days in 28-day cycles, in combination with daily thalidomide and celecoxib | 5.9m | 12.6m | PFS4= 63% | Appears unbeneficial |
| Alexander 2013 | Thalidomide | VEGFR | Thalidomide 200 mg at bedtime daily beginning first day of RT, increased up to 1200mg as tolerated + RT 5/7 over 6 weeks up to 60 Gy | 3.7m | 10m (Historical control = 12.5, HR= 0.99, p=0.48) | / | No survival benefit associated |
| Penas-Prados 2015 | Thalidomide | VEGFR | Thalidomide 400 mg orally daily; irostretinoin (CRA) 40 mg/m2 orally twice daily on days 1-21, celecoxib (Cel) 400 mg orally twice daily, RT over 6 weeks to a total of 60 Gy, concurrently daily TMZ 75 mg/m2/day | all=11.6m TMZ=10.5m  TMZ+CRA=6.5m  TMZ+Cel=13.4m TMZ+Thal=7.7m  TMZ+CRA+Cel=11.6m TMZ+CRA+Thal=6.2m  TMZ+Cel+Thal=7.9m  TMZ+CRA+Cel+Thal=5.8m | TMZ=21m  TMZ+CRA=12m TMZ+Cel=18m TMZ+Thal=17m TMZ+CRA+Cel=23m TMZ+CRA+Thal=19m TMZ+Cel+Thal=20m TMZ+CRA+Cel+Thal=18 | PFS-6 73% for all | No survival benefit associated |
| Groot 2011 | Aflibercept | VEGFR | Aflibercept 4 mg/kg intravenously on day 1 of every 14-day cycle | 12w | 39w | PFS6=6.6% | Appears unbeneficial |
| NCT00895180 2017 | Ramucirumab, olaratumab | VEGFR/PDGFR | Ramucirumab/olaratumab intravenously (IV) over 1 hour on day 1 of 14 day courses | / | IG1=49.5w IG2=34.3w | / | Appears unbeneficial |
| Nabors 2010 | ABT-510 | VEGFR | ABT-510 20-200 mg/m2 dq daily, TMZ 75 mg/m2 daily, RT 60 Gy | 45.9w | 14.8m | / | Appears unbeneficial |
| Brown 2016 | Gefitinib + cediranib | EGFR VEGFR | Cediranib 30mg every day + 500mg gefitinib or matched placebo | IG=3.6m CG=2.8m (HR 0.72) | IG=7.2m CG=5.5m (HR 0.68) | / | No survival benefit observed |
| D'alessandris 2013 | Erlotinib + bevacizumab | EGFR VEGFR | Erlotinib 150 or 300 mg/day + bev (10 mg/kg iv every 2 weeks in 6-week cycles) | 10.5m | 17m | / | Appears beneficial |
| D'alessandris 2019 | Erlotinib + bevacizumab | EGFR VEGFR | Erlotinib 150 or 300 mg/day + bev (10 mg/kg iv every 2 weeks in 6-week cycles) / bev alone / bev + sirolimus | / | / | PFS-6 = 64.3% | Appears beneficial |
| Sathornsumetee 2010 | Erlotinib + bevacizumab | EGFR VEGFR | Erlotinib orally 200 or 500 mg/day + bev IV every 2 weeks (10mg/kg) | GBM=18w | GBM=44.6w | PFS-6 GBM=29.2% | Appears unbeneficial |
| Clarke 2014 | Erlotinib + bevacizumab | EGFR VEGFR | Fractionated radiotherapy to 60 Gy, with daily TMZ at 75 mg/m2/d and erlotinib 150–200 mg/d (or 500–600 mg/d for patients on enzyme-inducing antiepileptic drugs). Bevacizumab was given at 10 mg/kg every 2 weeks, starting ≥4 weeks after surgery. After radiotherapy, adjuvant TMZ was given at 200 mg/m2/d × 5d per 28-day cycle, with unchanged erlotinib and bevacizumab doses. Treatment continued until progression or for 12 months. | IG= 13.5m  Historical CG = 8.6m (p=0.03) | IG =19.8m  Historical CG= 18m (p=0.33) | / | No survival benefit observed |
| Cardona 2021 | Osimertinib + bevacizumab | EGFR VEGFR | Osimertinib 80 mg/day) and bevacizumab (15 mg/kg, every 3 weeks) | 5.1m | 9m | PFS-6 = 46.7% | Appears unbeneficial |
| Wu 2025 | Nimotuzumab + bevacizumab | EGFR VEGFR | Weekly intravenous infusions of 200 mg nimotuzumab until the end of radiotherapy, Bevacizumab was administered intravenously at a dose of 10 mg per kilogram of body weight every 2 weeks, starting at week 4 of radiotherapy, until disease progression, RT + TMZ | 18m | 28m | PFS6= 94.4%, PFS12=77.8% | Appears beneficial |
| Hasselbalch 2010 | Cetuximab + bevacizumab | EGFR VEGFR | Bevacizumab + irinotecan every 2 weeks + cetuximab on days 1,8,15,22 (loading dose 400 mg/m2 folllowed by 250 mg/m2) | 3.7m | 6.9m | PFS-6=33% | Appears unbeneficial |
| Reardon 2020 | Rindopepimut + bevacizumab | EGFR VEGFR | Vaccination with 500 μg of rindopepimut admixed with 150 μg GM-CSF or control (injection of 100 μg keyhole limpet hemocyanin) + bevacizumab 10mg/kg every 2 weeks | HR=0.72, p=0.22 | HR 0.53, p=0.01 | PFS-6 IG=28 CG=16 p=0.12 | Survival benefit observed |
| Phuphanich 2017 | MEDI-575 (tovetumab) | PDGFR | MEDI-575 25 mg/kg IV over 60 min every 21 days | 1.4m | 9.7m | PFS-6=15.4% | Appears unbeneficial |
| Andrews 2021 | IGF-1R autologous cell vaccine | IGFR | Autologous cell vaccine + antisense molecule against IGF-1R DNA/mRNA (IGF-1R AS ODN),IGF-1R/AS ODN; Surgery with tissue harvest and implantation 20 diffusion chambers in the rectus sheath with IGF-1R/AS ODN within 24 hours of craniotomy, implanted for 48 hours. (Four vaccine exposures were evaluated: lowest (10 chambers implanted for 24 hours); lower (10 / 48 hours); higher (20 / 24 hours); and highest (20 / 48 hours). Standard of care (SOC; ie, radiotherapy and temozolomide) was initiated after 4-6 weeks. Randomization was halted after patient 23 and subsequent patients received the highest exposure. SOC temozolomide + radiotherapy | IG=9.8m CG=6.5m (p=0.0003) | IG=17.3m CG=16.2 (p=0.3) | PFS12 IG=42% CG=28% | Apperas beneficial |
| Affronti 2018 | Rilotumumab | HGF | Rilotumumab (20mg/kg) and Bev (10 mg/kg) every 2 weeks) | 4.8m | 11.2m | / | Appears unbeneficial |
| Wen 2011 | Rilotumumab | HGF | AMG 102 10 or 20 mg/kg by intravenous infusion (over 30–60 min) every 2 weeks | IG1=4.1m IG2=4.3m | IG1=6.5m IG2=5.4m | / | Appears unbeneficial |
| Cloughesy 2017 | Onartuzumab | HGF | Onartuzumab 15mg/kg + bev 15 mg/kg OR placebo + bev 15mg/kg IV on day 1 of 3 weekly cycles until disease progression | IG=3.9m CG=2.9m (HR 1.06, p=0.744) | IG=8.8m CG=12.6m (HR 1.45 p=0.1389) | OS-9= IG 49.7% CG=57.2%; PFS-6 IG=33.9% CG=29% | No survival benefit associated |
| Lombardi 2019 | Regorafenib | Multikinase | regorafenib 160 mg orally once daily for the first 3 weeks of a 4-week cycle OR lomustine 100 mg/m2 once every 6 weeks | IG=2.0m CG=1.9m ( HR= 0.65 p=0.022) | IG=7.4m CG=5.6m (HR 0.6, p=0.0009) | OS-12 IG=38.9%; CG=15.0% PFS-6 IG= 16.9%; CG=8.3% | Survival benefit associated |
| Lombardi 2021 | Regorafenib | Multikinase | regorafenib 160 mg orally once daily for the first 3 weeks of a 4-week cycle | 2.3m | 10.2m | PFS6=18% | Appears unbeneficial |
| Caccesse 2024 | Regorafenib | Multikinase | Regorafenib was 160 mg/day for 3 consecutive weeks with 1 week off | 2.6m | 7.9m | OS12=32.2% PFS6= 13.4% | Appears unbeneficial |
| Tunbekici 2024 | Regorafenib | Multikinase | Regorafenib was administered orally at a dose of 160 mg (four 40 mg tablets) daily for 3 weeks within a 4-week cycle | 2.5m | 4.1m | / | Appears unbeneficial |
| Mazzarella 2022 | Regorafenib | Multikinase | / | 4m | 22m | / | Appears beneficial |
| Lai 2024 | Anlotinib | Multikinase | Radiotherapy (54̃60 Gy administered as 1.8-2.0 Gy fractions, 5 days per week) for 6 weeks, with concurrent oral TMZ (75 mg/m2 QD) and anlotinib (8 mg QD, d1̃d14 per 3 weeks), 28-day break,  adjuvant therapy was started with six cycles of TMZ (150–200mg/m2, d1–5/4wks) and eight cycles of anlotinib (10 mg QD, d1–14/3wks) ，and followed by anlotinib monotherapy until disease progression or intolerable toxicities | 10.9m | 17.3m | PFS-12=48.5% OS-12=81.8% | Appears beneficial |
| Wang 2024 | Anlotinib | Multikinase | 12 mg anlotinib orally once daily on days 1–14 out of 21 day cycle | 7m | 17m | / | Appears beneficial |
| Yang 2021 | Anlotinib | Multikinase | TMZ 150 mg/m2 for 7 days every 2 weeks + anlotinib 12mg for fourteen days every 3 weeks | 4.5m | 6.2m | PFS6=40.2% | Appears unbeneficial |
| Guan 2022 | Anlotinib | Multikinase | anlotinib 12 mg daily during hypofractionated stereotactic radiotherapy , adjuvant anlotinib day 1-14 every 3 weeks | / | / | PFS-12: 60%, 2 year PFS: 40% OS-12: 100%; OS-24: 80% | Appears unbeneficial |
| Cao 2021 | Anlotinib | Multikinase | Anlotinib (12mg qd) + TMZ 200mg/m2, 5 days on with 23 days off until disease progression or intolerable toxicity | 8.3m | 10.8m | / | Appears unbeneficial |
| She 2021 | Anlotinib | Multikinase | Anlotinib (12 mg daily, orally for 2 weeks, discontinued for 1 week, repeated every 3 weeks) combined with dose-dense TMZ (100 mg/m2 daily on a 28-day cycle, orally for 7 days on with 7 days off) | 6.1m | 11.9m | PFS6=55% | Appears unbeneficial |
| Yang 2022 | Anlotinib | Multikinase | TMZ 150 mg/m2 for 7 days every 2 weeks + anlotinib 12mg for fourteen days every 3 weeks | 4.2m | 9.1m | / | Appears unbeneficial |
| Zhao 2024 | Anlotinib | Multikinase | oral anlotinib 12 mg (weight ≥ 50 kg) or 10 mg (weight < 50 kg) on days 1–14, with each cycle repeated every 21 days, alone (IG1) or in combination with bevacizumab | Total= 5m IG1= 4.2m IG2= 8m | Total= 13.6m IG1= 15.0m IG2= 9.8m | / | Appears unbeneficial |
| Hainsworth 2010 | Sorafenib | Multikinase | radiotherapy + TMZ, followed by concurrent oral TMZ and sorafenib 400 mg orally twice daily for 6 months | 6m | 12m | 1 year PFS: 16% | Appears unbeneficial |
| Hassler 2014 | Sorafenib | Multikinase | sorafenib 400mg twice daily continuously after a dose escalation phase of 10 days until evidence of disease progression | 3m | 6m | / | Appears unbeneficial |
| Zustovich 2013 | Sorafenib | Multikinase | 400 mg sorafenib twice daily + TMZ 40 mg/m2/day until disease progression | 3.2m | 7.4m | PFS-6=26% | Appears unbeneficial |
| Galanis 2013 | Sorafenib | Multikinase | sorafenib 200 mg bid 5/7 days per week (escalated to 7/7 days) and bev 5mg/kg IV every 2 weeks | 2.9m | 5.6m | PFS-6= 20.4% | Appears unbeneficial |
| Schiff 2018 | Sorafenib | Multikinase | temsirolimus IV 25mg weekly + sorafenib 200 mg twice daily with dose escalation | IG2=2.7m IG3=4.3m IG4=1.9m | IG2=6.6m IG3=6.7m IG4=3.9m | PFS6 IG2=17.1% IG3=22.2% IG4=6.8% | Appears unbeneficial |
| Peereboom 2013 | Erlotinib + sorafenib | Multikinase | erlotinib 150 mg once daily + sorafenib 400 mg twice daily | HR 0.65, p=0.045 | 5.7m (Historical control= 5.2m p=0.1) | PFS-6=14% | No survival benefit observed |
| Chen 2020 | Erlotinib + sorafenib | Multikinase | patients initially received sorafenib at 200 mg BID and erlotinib at 100 mg QD | 1.8m | 5m | PFS6=18.8% OS12=31.6% | Appears unbeneficial |
| Dresemann 2010 | Imatinib | Multikinase | 600 mg/d Imatinib + 1000 mg/d HU (IG) OR 1500 mg/du HU (CG) | IG=6w CG=6w (p=0.56) | / | PFS-6: imatinib + HU=5%; HU=7% PFS-12: imatinib+HU=2%; HU=2% OS-6: imatinib + HU = 40%; HU=37% | No survival benefit associated |
| Reardon 2005 | Imatinib | Multikinase | imatinib 500 mg twice daily+ hydroxyurea 500mg twice daily on 28-day cycle | 14.4w | 48.9w | PFS-6=27% | Appears unbeneficial |
| Wen 2006 | Imatinib | Multikinase | Imatinib orally once or twice daily (600-800 mg) | / | / | PFS-6=3% | Appears unbeneficial |
| Raymond 2008 | Imatinib | Multikinase | oral imatinib once daily 600-1000 mg/d | 1.8m | 5.9m | OS-6=50% | Appears unbeneficial |
| Hassler 2014 | Imatinib | Multikinase | imatinib 400 mg per day | 3m | 6.2m | PFS-6=3% | Appears unbeneficial |
| Razis 2009 | Imatinib | Multikinase | imatinib 400 mg orally twice daily for 7 days, followed by definitive surgery 6-24 hours after last dose of imatinib | / | 6.2m | / | Appears unbeneficial |
| Sautter 2020 | Imatinib | Multikinase | IG1= imatininb 600 mg/day with hyprofractionated RT (2.5 Gy per fraction, 22 fractions) IG2= imatinib 600 mg/day alone or in combination with re-irradiation  CCNU added in case of tumour progression | IG1=2.8m IG2=2.1m | IG1=5m IG2=6.5m | / | Appears unbeneficial |
| Reardon 2009 | Imatinib | Multikinase | Imatinib 600 mg per day + hydroxyurea 500 mg twice a day | 5.6w | 26w | PFS-6=10.6% | Appears unbeneficial |
| Kreisl 2012 | Sunitinib | Multikinase | Sunitinib 37.5mg/day | / | IG1= 9.4m; IG2= 4.37m | PFS6 IG1 = 10.4%; IG2 = 0% | Appears unbeneficial |
| Dresemann 2005 | Imatinib | Multikinase | Imatinib 400 mg once daily + hydroxyurea 500 mg twice daily | 10w | 19w | PFS-6= 32%; 16% 2 year PFS | Appears unbeneficial |
| Balana 2014 | Sunitinib | Multikinase | Sunitinib monotherapy 37.5 mg/day for 8 weeks | 7.7w | 12.8w |  | Appears unbeneficial |
| Pan 2012 | Sunitinib | Multikinase | Sunitinib 50 mg daily for 4 weeks followed by 2 week rest, up to 9 cycles | 1.4m | 12.6m | PFS-6 GBM=12.5% | Appears unbeneficial |
| Hutterer 2014 | Sunitinib | Multikinase | Sunitinib 37.5mg daily | 2.2m | 9.2m | 12m-OS=27.5% | Appears unbeneficial |
| Janssen 2024 | Sunitinib | Multikinase | Control arm: lomustine once every six weeks at a dose of 110 mg/m2 (maximum 200 mg). Experimental arm: high-dose intermittent sunitinib at a dose of 300 mg Q1W | IG1=1.5m CG1=1.5m (p=0.59) IG2=1.4m CG2=1.6m (p=0.7) | IG1=6.5m CG1=4.7m (p=0.83) IG2=4.7m CG2=7m (p=0.88) | PFS6= 54% | No survival benefit associated |
| Faye 2023 | Sunitinib | Multikinase | 12.5 mg of Sunitinib daily for 1 week prior to (preconcurrent phase) and during chemoradiation (RT + TMZ) | 7.15m | 15m | / | Appears unbeneficial |
| Martinez-Garcia 2022 | Crizotinib | HGF | Daily oral crizotinib (200-400 mg) with standard RT and TMZ, sequential adjuvant TMZ | 10.7m | 22.6m | PFS-6=71.5% PFS-12=38.8% OS-6=91.5% OS-12=79.3% | Apperas beneficial |
| Sharma 2019 | Dovitinib | Multikinase | Dovitinib 500 mg | IG1=2m IG2=1.8m | IG1=8m IG2=4.3m | PFS6 IG1=12% IG2=0% | Appears unbeneficial |
| Lee 2019 | Ponatinib | Multikinase | Ponatinib 45 mg daily | 28 days | 3.22m | PFS-3=0% | Appears unbeneficial |
| NCT01137604 | Lenvatinib | Multikinase | IG= lenvatinib capsules 24 mg taken orally, once daily continuously in 28-day cycles control=bevacizumab 10 mg/kg administered intravenously every 2 weeks in 28-day cycles | IG=2.4m CG=2.8m | IG=7.5m CG=6.0m | PFS6 IG=21.2% CG=11.0% | Appears unbeneficial |
| Rha 2025 | Lenvatinib | Multikinase | oral lenvatinib 20 mg per day plus intravenous pembrolizumab 200 mg every 3 weeks | 3m | 8.6m | PFS6= 22%; OS-12= 33% | Appears unbeneficial |
| Reardon 2013 | Lapatinib + pazopanib | Multikinase | Pazopanib 400mg once daily plus lapatinib 1000 mg once daily | IG1=8w IG2=8.9w | / | PFS-6 IG1=0% IG2=15% | Appears unbeneficial |
| Iwamoto 2010 | Pazopanib | Multikinase | Pazopanib 800 mg orally daily on 28-day cycles | 2.76m | 8m | / | Appears unbeneficial |
| Lee 2015 | Vandetinib | Multikinase | Vandetanib 100mg or placebo once daily beginning 5-7 days before initiating radiation; Radiation with concurrent temozolomide 75 mg/m2 daily for 6 weeks, followed by 4 to 6 weeks of rest, and then temozolomide “maintenance” for 12 cycles (28-day cycle) | IG=7.7m CG=6.2m (p=0.6) | IG=15.9m CG=16.6m (p=0.8) | PFS-6 IG=58% CG=57%; OS-12 IG=68% CG=0.56 | No survival benefit associated |
| McNeill 2014 | Vandetinib | Multikinase | IG = vandetanib (300 mg/d NEIAED, 400 mg/d EIAED) in combination with carboplatin (AUC 6 q 4 weeks); CG= carboplatin alone (AUC 6 q 4 weeks) | IG=1.7m CG=0.9m | IG=5.4m CG=5.2m | / | No survival benefit |
| Kreisl 2012 | Vandetinib | Multikinase | Vandetanib 300 mg orally once daily on a continuous 4-week treatment cycle | 1.3m | 6.3m | PFS6=6.5% OS-6=56.3% | Appears unbeneficial |
| Chheda 2015 | Vandetinib | Multikinase | Vandetinib (dose escalation from 100mg to 300 mg daily) and sirolimus | 2.77m | 8.6m | PFS-6=2.1m | Appears unbeneficial |
| Franceschi 2012 | Dasatinib | Multikinase | Dasatinib 100mg/day escalated to 100mg twice daily  CCNU 110mg/m2 every 6 weeks for max 6 cycles | 1.35m | 6.4m | 7.7% PFS6 | Appears unbeneficial |
| Breen 2025 | Dasatinib | Multikinase | Cycle 1: concomitant RT and TMZ, plus either placebo (Group 1) or dasatinib (Group 2) taken once daily, beginning on the same day as RT and continuing for a total of 42 days. Cycle 2: a 28–42 day rest period. Cycles 3–8: of adjuvant TMZ, plus either placebo taken once daily or dasatinib taken once daily at the same dose level as the end of Cycle 1, and continuing for a total of 28 days per cycle. | IG=6.2m CG=7.85 (p=0.269) | IG=15.6m CG=19.3m (p=0.238) | / | No survival benefit |
| Lu-Emerson 2011 | Dasatinib | Multikinase | dasatinib 70–100 mg twice daily in combination with bevacizumab (n = 14), until tumor progression or unacceptable toxicity | 4w | 11.1w | / | Appears unbeneficial |
| Lassman 2015 | Dasatinib | Multikinase | Dasatinib 100mg twice daily | 1.7m | 7.9m | PFS-6=6% | Appears unbeneficial |
| Muhic 2013 | Nintedanib | Multikinase | Nintedanib 200 mg twice daily | 1m | 6m | / | Appears unbeneficial |
| Norden 2015 | Nintedanib | Multikinase | Nintedanib 200 mg twice daily | IG1=28 days IG2=28 days | IG1=6.9m IG2=2.6m | PFS6 IG1=0% IG2=0% | Appears unbeneficial |
| Wen 2018 | Cabozantinib | Multikinase | Cabozantinib 100 (IG1) or 140 (IG2) mg/day | IG1=3.3m IG2=2.3 | IG1=4.1m IG2=4.6m | / | Appears unbeneficial |
| Odia 2016 | Tandutinib + bevacizumab | Multikinase | Tandutinib monotherapy 500 mg twice daily for 14 days, bevacizumab 10 mg/kg infused on day 1 and 15 | 4.1m | 11m | PFS-6=23% | Appears unbeneficial |
| Batchelor 2017 | Tandutinib | Multikinase | Tandutinib 600mg bd | 1.9m | 8.8m | PFS-6 16% | Appears unbeneficial |

Appendix A:

Medline

1. exp *Glioblastoma/ or glioblastoma*.ti,ab.
2. gbm.ti,ab.
3. high grade glioma*.ti,ab.
4. hgg.ti,ab.
5. (glioma* adj2 grade IV).ti,ab.
6. (astrocytoma* adj grade IV).ti,ab.
7. 1 or 2 or 3 or 4 or 5 or 6
8. Exp *protein-tyrosine kinase/
9. (tyrosin* adj5 (kinase* or inhibitor*)).ti,ab.
10. Exp *protein kinase inhibitors/ or Exp *Receptor protein-tyrosine kinases/ or RTK adj5 inhibitor*.ti,ab. or receptor tyrosine kinase*.ti,ab. or ptk receptor*.ti,ab.
11. Exp *ErbB receptors/ or EGF*.ti,ab. or epidermal growth factor*.ti,ab. or ErbB*.ti,ab. or exp *erlotinib hydrochloride/ or erlotinib.ti,ab. or tarceva.ti,ab. or Exp *gefitinib/ or iressa.ti,ab. or gefitinib.ti,ab. or dacomitinib.ti,ab. or vizimpro.ti,ab. or afatinib.ti,ab. or cetuximab.ti,ab. or infigratinib.ti,ab. or mAb806.ti,ab. or nimotuzumab.ti,ab. or osimertinib.ti,ab. OR AZD9291.ti,ab. OR mereletinib.ti,ab. OR vandetanib.ti,ab. OR AG 556.ti,ab. OR neratinib.ti,ab. OR AEE788.ti,ab. OR lapatinib.ti,ab.
12. Exp *receptors, platelet-derived growth factor/ or (PDGF* or platelet derived growth factor* or platelet-derived growth factor* or thrombocyte growth factor* or imatinib or glivec or Gleevec or tandutinib or MLN518 or AG 1433 or AG1296).ti,ab.
13. Exp *vascular endothelial growth factor A or gd-vegf.ti,ab. or vegf*.ti,ab. or vascular endothelial growth factor*.ti,ab. or vascular permeability factor*.ti,ab. or vasculotropin*.ti,ab. or valatanib.ti,ab. or sunitinib.ti,ab. or cabozatinib*.ti,ab. or cediranib.ti,ab. or bevacizumab.ti,ab. or pazopanib.ti,ab. or thalidomide.ti,ab. or SU 1498.ti,ab. or XL184.ti,ab.
14. Exp *receptor, IGF type 1/ or exp *somatomedins/ or insulin like growth factor 1.ti,ab. or insulin like growth factor I.ti,ab. or IGF-1*.ti,ab. or IGF-I*.ti,ab. or insulin-like growth factor 1.ti,ab. or insulin-like growth factor I.ti,ab. or somatomedin*.ti,ab. or exp *sunitinib/ or sunitinib.ti,ab. or nvp aew541.ti,ab. or PQ 401.ti,ab. or picropodophyllin.ti,ab. or BMS 536924.ti,ab. or BMS 754807.ti,ab. or OSI 906.ti,ab. or AG 1024.ti,ab.
15. Exp *proto-oncogene proteins c-met/ or (met adj5 (protein* or kinase*)).ti,ab. or scatter factor receptor*.ti,ab. or HGF*.ti,ab. or hepatocyte growth factor*.ti,ab. or crizotinib*.ti,ab. or INCB28060.ti,ab. or onartuzumab.ti,ab. or plb-1001.ti,ab.
16. Exp *receptors, fibroblast growth factor/ or fgf*.ti,ab. or fibroblast growth factor* or heparin binding growth factor*.ti,ab. or infigratinib.ti,ab. or AZD4547.ti,ab.
17. 8 or 9 or 10 or 11 or 12 or 13 or 14 or 15 or 16
18. exp antineoplastic agents/ or anticancer agent*.ti,ab. or antineoplastic agent*.ti,ab. or antitumour agent*.ti,ab. or antitumor agent*.ti,ab. or anti-cancer agent*.ti,ab. or anti-neoplastic agent*.ti,ab. or anti-tumour agent*.ti,ab. or anti-tumor agent*.ti,ab. Or medic*.ti,ab.or therap*.ti,ab. or exp Drug Therapy/ or drug*.ti,ab. Or treat*.ti,ab. Or genom*.ti,ab. Or gene*.ti,ab. or exp *Genetic Therapy/ or vaccin*.ti,ab. or exp *Cancer Vaccines/ or chemo*.ti,ab. Or exp *Immunotherapy/ or immunotherap*.ti,ab.
19. 17 and 18
20. surviv*.ti,ab. or exp Treatment Outcome/ or Survival Analysis/ or Survival Rate/ or disease free.ti,ab. Or progress* free.ti,ab. Or death.ti,ab. or Death/ or Mortality/ or mortalit*.ti,ab. Or Life Expectancy/ or life expectancy.ti,ab. Or exp *Remission Induction/ or remission.ti,ab. Or recur*.ti,ab. Or regress*.ti,ab.
21. 7 and 19 and 20
22. Randomized Controlled Trials as Topic/ or Randomized Controlled Trial/ or Random Allocation/ or Double-Blind Method/ or Single-Blind Method/ or Clinical Trial/ or clinical trial, phase i.pt. or clinical trial, phase ii.pt. or clinical trial, phase iii.pt. or clinical trial, phase iv.pt. or controlled clinical trial.pt. or randomized controlled trial.pt. or multicenter study.pt. or clinical trial.pt. or exp *Clinical Trial/ or (clinical adj trial$).tw. or ((singl$ or doubl$ or treb$ or tripl$) adj (blind$3 or mask$3)).tw. or Placebos/ or placebo$.tw. or randomly allocated.tw. or (allocated adj2 random$).tw.
23. case report.tw. or Letter/ or Historical Article/
24. 22 not 23
25. Epidemiologic Studies/ or exp *Case-Control Studies/ or exp *Cohort Studies/ or Case control.tw. or (cohort adj (study or studies)).tw. or Cohort analy$.tw. or (Follow up adj (study or studies)).tw. or (observational adj (study or studies)).tw. or Longitudinal.tw. or Retrospective.tw. or Cross sectional.tw. or Cross-Sectional Studies/
26. 24 or 25
27. 26 and 21

EMBASE

1. Glioblastoma*.ti,ab. or exp *glioblastoma/
2. gbm.ti,ab.
3. (high grade adj2 glioma*).ti,ab.
4. hgg.ti,ab.
5. (grade IV adj2 glioma*).ti,ab.
6. (grade IV adj2 astrocytoma*).ti,ab.
7. 1 or 2 or 3 or 4 or 5 or 6
8. Exp *protein tyrosine kinase/
9. (tyrosin* adj5 (kinase* or inhibitor*)).ti,ab.
10. Exp *protein kinase inhibitors/ or (RTK adj5 inhibitor*).ti,ab. or receptor tyrosine kinase*.ti,ab. or ptk receptor*.ti,ab.
11. Exp *epidermal growth factor receptor/ or Exp *epidermal growth factor receptor kinase inhibitor or EGF*.ti,ab. or epidermal growth factor*.ti,ab. or ErbB*.ti,ab. or exp *erlotinib/ or erlotinib.ti,ab. or tarceva.ti,ab. or exp *gefitinib/ or iressa.ti,ab. or gefitinib.ti,ab. or dacomitinib.ti,ab. or vizimpro.ti,ab. or afatinib.ti,ab. or Cetuximab.ti,ab. or infigratinib.ti,ab. or nimotuzumab.ti,ab. or osimertinib.ti,ab. or AZD9291.ti,ab. or mereletinib.ti,ab. or vandetanib.ti,ab. or AG 556.ti,ab. or neratinib.ti,ab. or AEE788.ti,ab. or lapatinib.ti,ab.
12. Exp *vasculotropin/ or exp *vasculotropin receptor/ or gd-vegf.ti,ab. or glioma derived vascular endothelial cell growth factor*.ti,ab. or glioma-derived vascular endothelial cell growth factor*.ti,ab. or vegf*.ti,ab. or vascular endothelial growth factor*.ti,ab. or vascular permeability factor*.ti,ab. or vasculotropin*.ti,ab. or valatanib.ti,ab. or sunitinib.ti,ab. or cabozatinib.ti,ab. or cediranib.ti,ab. or bevacizumab.ti,ab. or pazopanib.ti,ab. or thalidomide.ti,ab. or SU 1498.ti,ab. or XL184.ti,ab.
13. Exp *somatomedin C receptor/ or insulin like growth factor 1.ti,ab. or insulin like growth factor I.ti,ab. or insulin like growth factor.ti,ab. or IGF-1*.ti,ab. or IGF-I*.ti,ab. or insulin-like-growth factor.ti,ab. or somatomedin*.ti,ab. or exp sunitinib/ or sunitinib.ti,ab. or nvp aew541.ti,ab. or PQ 401.ti,ab. or picropodophyllin.ti,ab. or BMS 536924.ti,ab. or BMS 754807.ti,ab. or OSI 906.ti,ab. or AG 1024.ti,ab.
14. Exp *platelet derived growth factor/ or (PDGF* or plate derived growth factor* or platelet-derived growth factor* or thrombocyte growth factor receptor* or mAb806 or imatinib glivec or Gleevec or tandutinib or MLN518 or AG 1433 or AG 1296).ti,ab.
15. Exp *fibroblast growth factor receptor/ or fgf*.ti,ab. or fibroblast growth factor* or heparin binding growth factor*.ti,ab. or infigratinib.ti,ab. or AZD4547.mp
16. Exp *scatter factor receptor/ or met adj5 (protein* or kinase*).ti,ab. or scatter factor receptor*.ti,ab. or HGF*.ti,ab. or hepatocyte growth factor*.ti,ab. or crizotinib*.ti,ab. or INCB28060.ti,ab. or onartuzumab.ti,ab. or plb-1001.ti,ab.
17. 8 or 9 or 10 or 11 or 12 or 13 or 14 or 15 or 16
18. medic*.ti,ab. Or therap*.ti,ab. Or exp *phase 4 clinical trial/ or exp phase 2 clinical trial/ or exp *controlled clinical trial/ or exp *clinical trial/ or exp *superiority trial/ or exp *pragmatic trial/ or exp *"phase 1 clinical trial (topic)"/ or exp *"controlled clinical trial (topic)"/ or exp *phase 3 clinical trial/ or exp *"phase 4 clinical trial(topic)"/ or exp "randomized controlled trial (topic)"/ or exp randomized controlled trial/ or exp *"phase 2 clinical trial (topic)"/ or exp *non-inferiority trial/ or exp *"phase 3 clinical trial (topic)"/ or exp *phase 1 clinical trial/ or drug*.ti,ab. Or treat*.ti,ab. Or exp *antineoplastic agent/ or antitumor?r agent*.ti,ab. or anticancer agent*.ti,ab. or antineoplastic agent*.ti,ab. or anti-cancer agent*.ti,ab. or anti-neoplastic agent*.ti,ab. or anti-tumo?r agent*.ti,ab. Or genom*.ti,ab. Or gene*.ti,ab. Or vaccination/ or vaccin*.ti,ab. or vaccine/ or exp *chemoradiotherapy/ or chemo*.ti,ab. or exp *cancer chemotherapy/ or exp *immunotherapy/ or immunotherap*.ti,ab.
19. 17 and 18
20. exp *mean survival time/ or exp *survival bias/ or exp *distant recurrence free survival/ or exp *survival time/ or exp *cancer specific survival/ or exp *recurrence free survival/ or exp *cancer free survival/ or exp *short term survival/ or exp *metastasis free survival/ or exp *progression free survival/ or exp *survival rate/ or exp *disease free survival/ or surviv*.ti,ab. or exp *cancer survival/ or exp *median survival time/ or exp *survival analysis/ or exp *overall survival/ or exp *event free survival/ or exp *survival prediction/ or exp *long term survival/ or exp *survival/ or disease free.ti,ab. Or progress* free.ti,ab. Or death/ or death.ti,ab. Or mortality/ or mortalit*.ti,ab. Or life expectancy.ti,ab. or exp life expectancy/ or exp *remission/ or remission.ti,ab. Or recur*.ti,ab. or exp *recurrent disease/ or exp *cancer recurrence/ or regress*.ti,ab.
21. 7 and 19 and 20
22. clinical trial/ or randomized controlled trial/ or controlled clinical trial/ or multicenter study/ or phase 3 clinical trial/ or phase 4 clinical trial/ or exp *randomization/ or single blind procedure/ or double blind procedure/ or crossover procedure/ or placebo/ or randomi?ed controlled trial$.tw. or rct.tw. or (random$ adj2 allocat$).tw. or single blind$.tw. or double blind$.tw. or ((treble or triple) adj blind$).tw. or placebo$.tw. or prospective study/
23. case study/ or case report.tw. or abstract report/ or letter/ or Conference proceeding.pt. or Conference abstract.pt. or Editorial.pt. or Letter.pt. or Note.pt.
24. 22 not 23
25. clinical study/ or case control study/ or family study/ or longitudinal study/ or retrospective study/
26. prospective study/
27. "randomized controlled trial (topic)"/
28. 26 not 27
29. cohort analysis/ or (Cohort adj (study or studies)).ti,ab. Or (Case control adj (study or studies)).tw. or (follow up adj (study or studies)).tw. or (observational adj (study or studies)).tw. or (epidemiologic$ adj (study or studies)).tw. or (cross sectional adj (study or studies)).tw.
30. 25 or 28 or 29
31. 30 or 24
32. 21 and 31

Web of Science

1. TS=(*glioblastoma* OR *gbm* OR "high grade" NEAR/5 glioma OR *hgg* OR "grade IV" NEAR/5 glioma OR "high grade" NEAR/5 astrocytoma* OR "grade IV" NEAR/5 astrocytoma*)
2. TS=((tyrosin* NEAR/5 (kinase* or inhibitor*)) OR receptor tyrosine kinase* OR RTK OR EGF* or epidermal growth factor* OR ErbB* OR erlotinib OR tarceva OR gefitinib OR iressa OR dacomitinib OR vizimpro OR afatinib OR cetuximab OR infigratinib OR mAb805 OR Nimotuzumab OR Osimertinib OR AZD9291 OR mereletinib OR vandetanib OR AG 556 OR neratinib OR AEE788 OR lapatinib PDGF* OR platelet-derived growth factor* OR thrombocyte growth factor receptor* OR imatinib OR glivec OR Gleevec OR tandutinib OR MLN518 OR AG 1433 OR AG 1296 OR VEGF* OR vascular endothelial growth factor* OR vasculotropin OR valatanib OR sunitinib OR cabozatinib OR XL184 OR cediranib OR bevacuzimab OR pazopanib OR thalidomide OR SU 1498 OR IGF-1 or IGF-I OR somatomedin OR insulin-like growth factor 1 OR insulin like growth factor 1 OR insulin-like growth factor I OR insulin like growth factor I OR sunitinib OR nvp aew541 OR PQ401 OR picropodophyllin OR BMS 536924 OR BMS 754807 OR OSI 906 OR AG 1024 OR met NEAR/5 (protein* or kinase*) OR scatter factor* OR crizotinib OR INCB28060 OR onartuzumab OR plb-1001 OR FGF* OR fibroblast growth factor* OR infigratinib OR AZD4547 or hepatocyte growth factor* or HGF*)
3. TS=(medic* OR therap* OR trial* OR drug* OR treat* OR antineoplastic agent* OR antitumo?r agent* OR anticancer agent* OR genom* OR gene* OR vaccin* OR chemo* OR immunotherap*)
4. TS=(surviv* OR disease free OR progress* free OR death OR mortalit* OR life expectancy OR remission OR recur* OR regress*)
5. TS=(randomised OR randomized OR randomisation OR randomization OR placebo* OR (random* AND (allocat* OR assign*)) OR (blind* AND (single OR double OR treble OR triple)))
6. TS=(epidemiologic* OR case control OR cohort OR follow up OR observational OR longitudinal OR retrospective OR prospective OR cross sectional)
7. #2 AND #3
8. #1 AND #7 AND #4
9. #5 OR #6
10. #8 AND #9

Scopus

( ( TITLE-ABS-KEY ( ( glioblastoma* )  OR  ( gbm )  OR  ( "high grade"  W/5  glioma* )  OR  ( hgg )  OR  ( "grade iv"  W/5  glioma* )  OR  ( "high grade"  W/5  astrocytoma* )  OR  ( "grade iv"  W/5  astrocytoma* ) ) )  AND

( ( TITLE-ABS- KEY ( ( tyrosin*  W/5  ( kinase*  OR  inhibitor* ) )  OR  ( rtk  W/5  inhibitor )  OR  ( "receptor tyrosine kinase" )  OR  ( egf* )  OR  ( "epidermal growth factor*" )  OR  ( erbb* )  OR  ( erlotinib )  OR  ( gefitinib )  OR  ( iressa )  OR  ( dacominitib )  OR  ( vizimpro )  OR  ( afatinib )  OR  ( cetuximab )  OR  ( infigratinib )  OR  ( mab806 )  OR  ( nimotuzumab )  OR  ( osimertinib )  OR  ( azd9291 )  OR  ( mereletinib )  OR  ( vandetanib )  OR  ( "AG 556" )  OR  ( neratinib )  OR  ( aee788 )  OR  ( lapatinib )  OR  ( pdgf* )  OR  ( "platelet derived growth factor*" )  OR  ( "platelet-derived growth factor*" )  OR  ( "thrombocyte growth factor*" )  OR  ( imatinib )  OR  ( glivec )  OR  ( gleevec )  OR  ( tandutinib )  OR  ( mln518 )  OR  ( "AG 1433" )  OR  ( "AG 1296" )  OR  ( "vascular endothelial growth factor*" )  OR  ( vegf* )  OR  ( "gd-VEGF*" )  OR  ( "vascular permeability factor*" )  OR  ( vasculotropin* )  OR  ( valatanib )  OR  ( sunitinib )  OR  ( cabozatinib )  OR  ( "XL 1498" )  OR  ( cerdiranib )  OR  ( bevacizumab )  OR  ( pazopanib )  OR  ( thalidomide )  OR  ( "SU 1498" )  OR  ( somatomedin* )  OR  ( "insulin like growth factor 1" )  OR  ( "insulin like growth factor I" )  OR  ( "insulin-like-growth factor 1" )  OR  ( "insulin-like-growth factor I" )  OR  ( "IGF-1*" )  OR  ( "IGF-I*" )  OR  ( sunitinib )  OR  ( "nvp aew541" )  OR  ( "PQ 401" )  OR  ( picropodophyllin )  OR  ( "BMS 536924" )  OR  ( "BMS 754807" )  OR  ( "OSI 906" )  OR  ( "AG 1024" )  OR  ( met  AND w  AND /5  ( protein*  OR  kinase* ) )  OR  ( "scatter factor receptor*" )  OR  ( hgf* )  OR  ( "hepatocyte growth factor*" )  OR  ( crizotinib )  OR  ( incb28060 )  OR  ( onartuzumab )  OR  ( "plb-1001" )  OR  ( fgf* )  OR  ( "fibroblast growth factor*" )  OR  ( "heparin binding growth factor*" )  OR  ( azd4547 ) ) )  AND  ( TITLE-ABS-KEY ( ( medic* )  OR  ( therap* )  OR  ( trial* )  OR  ( drug* )  OR  ( treat* )  OR  ( "antineoplastic agent*" )  OR  ( "anticancer agent*" )  OR  ( "antitumo?r agent*" )  OR  ( genom* )  OR  ( gene* )  OR  ( vaccin* )  OR  ( chemo* )  OR  ( immunotherap* ) ) ) )  AND  ( TITLE-ABS-KEY ( ( surviv* )  OR  ( "disease free" )  OR  ( "progress* free" )  OR  ( death )  OR  ( mortalit* )  OR  ( "life expectancy" )  OR  ( remission )  OR  ( recur* )  OR  ( regress* ) ) ) )  AND  ( ( TITLE-ABS-KEY ( {clinical-trial}  OR  {controlled-trial}  OR  randomi*  OR  randomly  OR  ( random  W/4  ( allocat*  OR  distribut*  OR  assign* ) )  OR  {placebo}  OR  {trial}  OR  {groups}  OR  {subgroups} )  OR  TITLE ( rct ) )  OR  ( TITLE-ABS-KEY ( ( epidemiologic* )  OR  ( "case control" )  OR  ( cohort )  OR  ( "follow up" )  OR  ( observational )  OR  ( longitudinal )  OR  ( retrospective )  OR  ( prospective )  OR  ( "cross sectional" ) ) ) )

Appendix B

| **Paper** |  |
| --- | --- |
| Cai 2024 | Results not stratified for HGG and glioblastoma |
| Guo 2023 | Results not stratified for HGG and glioblastoma |
| Picca 2023 | Results not stratified for HGG and glioblastoma |
| Poklepovic 2024 | Results not stratified for HGG and glioblastoma |
| Sun 2023 | Results not stratified for HGG and glioblastoma |
| Den 2013 | Results not stratified for HGG and glioblastoma |
| Hottinger 2013 | Results not stratified for HGG and glioblastoma |
| Reardon 2011 | Results not stratified for HGG and glioblastoma |
| Short 2001 | Results not stratified for HGG and glioblastoma |
| Snelling 1995 | Results not stratified for HGG and glioblastoma |
| Stragliotto 1996 | Results not stratified for HGG and glioblastoma |
| Wuthrick 2014 | Results not stratified for HGG and glioblastoma |
| Zeiner 2019 | Results not stratified for HGG and glioblastoma |

Appendix C

- name of first author
- year of publication
- title
- country of study
- full text or abstract format
- design
- endpoints
- RTK target
- comparison group
- inclusion criteria
- number of patients
- median age
- biological sex
- ethnicity
- mean Karnofsky Performance Status
- newly diagnosed or recurrent disease
- percentage with complete surgical resection
- *MGMT* promoter methylation status
- median time from diagnosis to therapy
- objective response rate
- median PFS
- median OS
- additional survival measures
- relevant statistical analyses performed

Appendix D

| **Study** | Was true randomization used for assignment of participants to treatment groups? | Was allocation to treatment groups concealed? | Were treatment groups similar at the baseline? | Were participants blind to treatment assignment? | Were those delivering treatment blind to treatment assignment? | Were outcomes assessors blind to treatment assignment | Were treatment groups treated identically other than the intervention of interest? | Was follow up complete and if not, were differences between groups in terms of their follow up adequately described and analyzed? | Were participants analyzed in the groups to which they were randomized? | Were outcomes measured in the same way for treatment groups? | Were outcomes measured in a reliable way? | Was appropriate statistical analysis used? | Was the trial design appropriate, and any deviations from the standard RCT design (individual randomization, parallel groups) accounted for in the conduct and analysis of the trial? | Overall appraisal |  |
| --- | --- | --- | --- | --- | --- | --- | --- | --- | --- | --- | --- | --- | --- | --- | --- |
| Reardon 2015 | Y | U | Y | N | N | N | Y | Y | Y | Y | Y | Y | Y | I |  |
| Westphal 2015 | Y | U | Y | N | N | N | Y | Y | Y | Y | Y | Y | Y | I |  |
| Lassman 2023 | Y | U | Y | U | U | Y | Y | Y | Y | Y | Y | Y | Y | I |  |
| van den Bent 2020 | Y | U | Y | U | U | Y | Y | Y | Y | Y | Y | Y | Y | I |  |
| Weller 2017 | Y | Y | Y | Y | Y | Y | Y | Y | Y | Y | Y | Y | Y | I |  |
| Reardon 2020 | Y | Y | Y | Y | Y | Y | Y | Y | Y | Y | Y | Y | Y | I |  |
| Batchelor 2013 | Y | N | Y | N | N | Y | Y | Y | Y | Y | Y | Y | Y | I |  |
| Hsieh 2022 | Y | U | U | U | U | U | Y | U | U | Y | Y | Y | Y | I | Abstract only |
| Batchelor 2023 | Y | Y | Y | Y | U | Y | Y | Y | Y | Y | Y | Y | Y | I |  |
| Janssen 2024 | Y | N | Y | N | N | N | Y | Y | Y | Y | Y | Y | Y | I |  |
| Duerinck 2016 | Y | N | Y | N | N | N | Y | Y | Y | Y | Y | Y | Y | I |  |
| Duerinck 2018 | Y | U | Y | U | U | U | Y | Y | Y | Y | Y | Y | Y | I |  |
| Lombardi 2019 | Y | N | Y | N | N | N | Y | Y | Y | Y | Y | Y | Y | I |  |
| Dresemann 2010 | Y | N | Y | N | N | N | Y | Y | Y | Y | Y | Y | Y | I |  |
| Cloughesy 2017 | Y | U | Y | Y | Y | Y | Y | Y | Y | Y | Y | Y | Y | I |  |
| Solomon 2013 | Y | Y | Y | Y | Y | Y | Y | Y | Y | Y | Y | Y | Y | I |  |
| Brown 2016 | Y | Y | Y | Y | Y | Y | Y | Y | Y | Y | Y | Y | Y | I |  |
| NCT01137604 2022 | Y | N | Y | N | N | N | Y | Y | Y | Y | Y | Y | Y | I |  |
| Lee 2015 | Y | N | Y | N | N | N | Y | Y | Y | Y | Y | Y | Y | I |  |
| McNeill 2014 | Y | U | U | U | U | U | Y | U | U | Y | Y | Y | Y | I | Abstract only |
| Breen 2025 | Y | Y | Y | Y | U | U | Y | Y | Y | Y | Y | Y | Y | I |  |

| **Study** | **Is it clear in the study what is the ‘cause’ and what is the ‘effect’ (i.e. there is no confusion about which variable comes first)?** | **Were the participants included in any comparisons similar?** | **Were the participants included in any comparisons receiving similar treatment/care, other than the exposure or intervention of interest?** | **Was there a control group?** | **Were there multiple measurements of the outcome both pre and post the intervention/exposure?** | **Was follow up complete and if not, were differences between groups in terms of their follow up adequately described and analyzed?** | **Were the outcomes of participants included in any comparisons measured in the same way?** | **Were outcomes measured in a reliable way?** | **Was appropriate statistical analysis used?** | **Overall appraisal** |  |
| --- | --- | --- | --- | --- | --- | --- | --- | --- | --- | --- | --- |
| Raizer 2009 | Y | N/A | N/A | N | N/A | Y | N/A | Y | Y | I |  |
| Krishnan 2006 | Y | N/A | N/A | N | N/A | Y | N/A | Y | Y | I |  |
| Gallego 2014 | Y | N/A | N/A | N | N/A | Y | N/A | Y | Y | I |  |
| Yung 2010 | Y | Y | Y | N | N/A | Y | Y | Y | Y | I |  |
| Brown 2008 | Y | N/A | N/A | N | N/A | Y | N/A | Y | Y | I |  |
| Peereboom 2010 | Y | N/A | N/A | N | N/A | Y | N/A | Y | Y | I |  |
| Prados 2009 | Y | Y | Y | Y | N/A | Y | Y | Y | Y | I | Historical control |
| Wen 2014 | Y | N/A | N/A | N | N/A | Y | N/A | Y | Y | I |  |
| de Groot 2008 | Y | N/A | N/A | N | N/A | Y | N/A | Y | Y | I |  |
| van den Bent 2009 | Y | Y | Y | Y | N/A | Y | Y | Y | Y | I | Historical control |
| Charkravarti 2013 | Y | Y | Y | Y | N/A | Y | Y | Y | Y | I | Historical control |
| Kreisl 2008 | Y | N/A | N/A | N | N/A | Y | N/A | Y | Y | I |  |
| Rich 2004 | Y | N/A | N/A | N | N/A | Y | N/A | Y | Y | I |  |
| Uhm 2011 | Y | N/A | N/A | N | N/A | Y | N/A | Y | Y | I |  |
| Chi 2020 | Y | N/A | N/A | N | N/A | Y | N/A | Y | Y | I |  |
| Sepulveda-Sanchez 2017 | Y | Y | Y | N | N/A | Y | Y | Y | Y | I |  |
| Solomon 2014 | Y | N/A | N/A | N | N/A | Y | N/A | Y | Y | I |  |
| Wang 2016 | Y | N/A | N/A | N | N/A | Y | N/A | Y | Y | I |  |
| Du 2019 | Y | N/A | N/A | N | N/A | Y | N/A | Y | Y | I |  |
| Brady 1992 | Y | N/A | N/A | N | N/A | Y | N/A | Y | Y | I |  |
| Li 2010 | Y | Y | Y | Y | N/A | Y | Y | Y | Y | I | Historical control, abstract only |
| Reardon 2013 | Y | N/A | N/A | N | N/A | Y | N/A | Y | Y | I |  |
| Ramos 2006 | Y | N/A | N/A | N | N/A | Y | N/A | Y | Y | I |  |
| Goff 2019 | Y | N/A | N/A | N | N/A | Y | N/A | Y | Y | I |  |
| Bagley 2024 | Y | N/A | N/A | N | N/A | Y | N/A | Y | Y | I |  |
| Gan 2018 | Y | N/A | Y | Y | N/A | Y | Y | Y | Y | I |  |
| Lassman 2019 | Y | N/A | N/A | N | N/A | Y | N/A | Y | Y | I |  |
| Caccese 2020 | Y | N/A | N/A | N | N/A | Y | N/A | Y | Y | I |  |
| Desjardins 2017 | Y | N/A | N/A | N | N/A | Y | N/A | Y | Y | I | Abstract only |
| Schuster 2015 | Y | N/A | N/A | N | N/A | Y | N/A | Y | Y | I | Historical control |
| Sampson 2010 | Y | Y | Y | Y | N/A | Y | Y | Y | Y | I |  |
| Spiekman 2023 | Y | N/A | N/A | N | N/A | Y | N/A | Y | Y | I |  |
| Choi 2023 | Y | N/A | N/A | N | N/A | Y | N/A | Y | Y | I |  |
| D'Alessandris 2013 | Y | N/A | N/A | N | N/A | Y | N/A | Y | Y | I |  |
| D'Alessandris 2019 | Y | N/A | N/A | N | N/A | Y | N/A | Y | Y | I | Abstract only |
| Clarke 2014 | Y | Y | N/A | Y | N/A | Y | Y | Y | Y | I | Historical control |
| Cardona 2021 | Y | N/A | N/A | N | N/A | Y | N/A | Y | Y | I |  |
| Hasselbalch 2010 | Y | N/A | N/A | N | N/A | Y | N/A | Y | Y | I |  |
| Sathornsumustee 2010 | Y | N/A | N/A | N | N/A | Y | N/A | Y | Y | I |  |
| Wu 2025 | Y | N/A | N/A | N | N/A | Y | N/A | Y | Y | I |  |
| Marx 2001 | Y | N/A | N/A | N | N/A | Y | N/A | Y | Y | I |  |
| Fine 2003 | Y | N/A | N/A | N | N/A | Y | N/A | Y | Y | I |  |
| Fadul 2008 | Y | N/A | Y | N | N/A | Y | Y | Y | Y | I |  |
| Baumann 2004 | Y | N/A | Y | N | N/A | Y | Y | Y | Y | I |  |
| Chang 2004 | Y | N/A | N/A | N | N/A | Y | N/A | Y | Y | I |  |
| Riva 2007 | Y | Y | Y | Y | N/A | Y | Y | Y | Y | I |  |
| Groves 2007 | Y | N/A | N/A | N | N/A | Y | N/A | Y | Y | I |  |
| Kesari 2008 | Y | N/A | N/A | N | N/A | Y | N/A | Y | Y | I |  |
| Alexander 2013 | Y | N/A | N/A | N | N/A | Y | N/A | Y | Y | I |  |
| Penas-Prados 2015 | Y | Y | Y | N | N/A | Y | Y | Y | Y | I |  |
| Batchelor 2010 | Y | N/A | N/A | N | N/A | Y | N/A | Y | Y | I |  |
| Gerstner 2015 | Y | Y | Y | N | N/A | Y | Y | Y | Y | I |  |
| Balana 2014 | Y | N/A | N/A | N | N/A | Y | N/A | Y | Y | I |  |
| Pan 2012 | Y | N/A | N/A | N | N/A | Y | N/A | Y | Y | I |  |
| Hutterer 2014 | Y | N/A | N/A | N | N/A | Y | N/A | Y | Y | I |  |
| Faye 2023 | Y | N/A | N/A | N | N/A | Y | N/A | Y | Y | I |  |
| Zhang 2018 | Y | N/A | N/A | N | N/A | Y | N/A | Y | Y | I | Abstract only |
| Zhang 2022 | Y | N/A | N/A | N | N/A | Y | N/A | Y | Y | I |  |
| Wang 2019 | Y | N/A | N/A | N | N/A | Y | N/A | Y | Y | I |  |
| Ge 2020 | Y | N/A | N/A | N | N/A | Y | N/A | Y | Y | I |  |
| Awada 2020 | Y | Y | Y | N | N/A | Y | Y | Y | Y | I |  |
| Lombardi 2021 | Y | N/A | N/A | N | N/A | Y | N/A | Y | Y | I |  |
| Mazzarella 2022 | Y | N/A | N/A | N | N/A | Y | N/A | Y | Y | I | Abstract only |
| Tunbekici 2024 | Y | N/A | N/A | N | N/A | Y | N/A | Y | Y | I |  |
| Caccese 2024 | Y | N/A | N/A | N | N/A | Y | N/A | Y | Y | I |  |
| Kalpathy Cramer 2017 | Y | N/A | N/A | N | N/A | Y | N/A | Y | Y | I |  |
| Schiff 2015 | Y | Y | Y | N | N/A | Y | Y | Y | Y | I |  |
| Wen/Cloughesy 2018 | Y | Y | Y | N | N/A | Y | Y | Y | Y | I |  |
| NCT00895180 2017 | Y | Y | Y | N | N/A | Y | Y | Y | Y | I |  |
| de Groot 2011 | Y | N/A | N/A | N | N/A | Y | N/A | Y | Y | I |  |
| Kreisl 2012 | Y | Y | Y | N | N/A | Y | Y | Y | Y | I |  |
| Dresemann 2005 | Y | N/A | N/A | N | N/A | Y | N/A | Y | Y | I |  |
| Reardon 2009 | Y | N/A | N/A | N | N/A | Y | N/A | Y | Y | I |  |
| Reardon 2005 | Y | N/A | N/A | N | N/A | Y | N/A | Y | Y | I |  |
| Wen 2006 | Y | N/A | N/A | N | N/A | Y | N/A | Y | Y | I |  |
| Raymond 2008 | Y | N/A | N/A | N | N/A | Y | N/A | Y | Y | I |  |
| Hassler 2014 | Y | N/A | N/A | N | N/A | Y | N/A | Y | Y | I |  |
| Razis 2009 | Y | N/A | N/A | N | N/A | Y | N/A | Y | Y | I |  |
| Sautter 2020 | Y | Y | Y | N | N/A | Y | Y | Y | Y | I |  |
| Odia 2016 | Y | N/A | N/A | N | N/A | Y | N/A | Y | Y | I |  |
| Batchelor 2017 | Y | N/A | N/A | N | N/A | Y | N/A | Y | Y | I |  |
| Phuphanich 2017 | Y | N/A | N/A | N | N/A | Y | N/A | Y | Y | I |  |
| Andrews 2021 | Y | Y | Y | Y | N/A | Y | Y | Y | Y | I | Historical cohort |
| Martinez-Garcia 2022 | Y | N/A | N/A | N | N/A | Y | N/A | Y | Y | I |  |
| Affronti 2018 | Y | N/A | N/A | N | N/A | Y | N/A | Y | Y | I |  |
| Wen 2011 | Y | Y | Y | N | N/A | Y | Y | Y | Y | I |  |
| Hainsworth 2010 | Y | N/A | N/A | N | N/A | Y | N/A | Y | Y | I |  |
| Zustovich 2013 | Y | N/A | N/A | N | N/A | Y | N/A | Y | Y | I |  |
| Hassler 2014 | Y | N/A | N/A | N | N/A | Y | N/A | Y | Y | I |  |
| Schiff 2018 | Y | Y | Y | N | N/A | Y | N/A | Y | Y | I |  |
| Galanis 2013 | Y | N/A | N/A | N | N/A | Y | N/A | Y | Y | I |  |
| Peereboom 2013 | Y | N/A | N/A | N | N/A | Y | N/A | Y | Y | I |  |
| Chen 2020 | Y | N/A | N/A | N | N/A | Y | N/A | Y | Y | I |  |
| Franceschi 2012 | Y | N/A | N/A | N | N/A | Y | N/A | Y | Y | I |  |
| Lu-Emerson 2011 | Y | N/A | N/A | N | N/A | Y | N/A | Y | Y | I |  |
| Lassman 2015 | Y | Y | Y | N | N/A | Y | Y | Y | Y | I |  |
| Lai 2024 | Y | N/A | N/A | N | N/A | Y | N/A | Y | Y | I |  |
| Cao 2021 | Y | N/A | N/A | N | N/A | Y | N/A | Y | Y | I | Abstract only |
| Yang 2021 | Y | N/A | N/A | N | N/A | Y | N/A | Y | Y | I |  |
| Guan 2022 | Y | N/A | N/A | N | N/A | Y | N/A | Y | Y | I |  |
| Zhao 2024 | Y | Y | Y | N | N/A | Y | Y | Y | Y | I |  |
| Wang 2024 | Y | N/A | N/A | N | N/A | Y | N/A | Y | Y | I |  |
| She 2021 | Y | N/A | N/A | N | N/A | Y | N/A | Y | Y | I |  |
| Yang 2022 | Y | N/A | N/A | N | N/A | Y | N/A | Y | Y | I |  |
| Sharma 2019 | Y | N | Y | N | N/A | Y | Y | Y | Y | I |  |
| Lee 2019 | Y | N/A | N/A | N | N/A | Y | N/A | Y | Y | I |  |
| Nabors 2010 | Y | N/A | N/A | N | N/A | Y | N/A | Y | Y | I |  |
| Kreisl 2012 | Y | N/A | N/A | N | N/A | Y | N/A | Y | Y | I |  |
| Chheda 2015 | Y | N/A | N/A | N | N/A | Y | N/A | Y | Y | I |  |
| Muhic 2013 | Y | Y | Y | N | N/A | Y | Y | Y | Y | I |  |
| Norden 2013 | Y | Y | Y | N | N/A | Y | Y | Y | Y | I |  |
| Reardon 2013 | Y | Y | Y | N | N/A | Y | Y | Y | Y | I |  |
| Rha 2025 | Y | N/A | N/A | N | N/A | Y | N/A | Y | Y | I |  |
| Iwamoto 2010 | Y | N/A | N/A | N | N/A | Y | N/A | Y | Y | I |  |

Appendix E: Study Funders

| **Study** |  |
| --- | --- |
| van den Bent 2009 | N/A |
| Raizer 2009 | 5-U01CA62399-09 (J.J.R., L.E.A., A.B.L., and L.M.D.); NABTC # CA62399 and Member # CA62422, GCRC Grant # M01-RR00079 (S.M.C., K.R.L., and M.D.P.); CA62426 (J.G.K.); CA62412, GCRC Grant # CA16672 (W.K.A.Y. and M.R.G.); U01CA62407-08 (P.Y.W.), U01CA62421–08, GCRC Grant # M01 RR03186 (M.M. and H.I.R.); U01CA62405, GCRC Grant # M01-RR00056 (F.L.); U01 CA62399, GCRC Grant # M01-RR0865 (T.F.C.). |
| Yung 2010 | Accelerate Brain Cancer Cure |
| Gallego 2014 | N/A |
| de Groot 2008 | N/A |
| Wen 2014 | Sontag Foundation, NCI, National Center for Advancing Translational Sciences |
| Peereboom 2010 | N/A |
| Brown 2008 | National Cancer Institute |
| Prados 2009 | National Cancer Institute |
| Krishnan 2006 | N/A |
| Chakravarti 2013 | National Cancer Institute |
| Kreisl 2008 | Novartis, AstraZeneca |
| Rich 2004 | National Cancer Institute |
| Uhm 2011 | N/A |
| Reardon 2015 | Boehringer Ingelheim |
| Chi 2020 | N/A |
| Sepulveda-Sanchez 2017 | Pfizer |
| Solomon 2013 | Center of Molecular Immunology, Cuban Ministry of Health |
| Solomon 2014 | N/A |
| Westphal 2015 | OncoScienceAG |
| Wang 2016 | N/A |
| Du 2019 | Biotech Pharmaceutical Co., Ltd |
| Ramos 2006 | N/A |
| Desjardins 2017 | N/A |
| Brady 1992 | N/A |
| Li 2010 | N/A |
| Spiekman 2023 | Stelvio for Life Foundation, the Dutch Cancer Society (grant number 10014), the Hartwig Medical Foundation (HMF), Amgen, AstraZeneca, Bayer, Bristol-Myers Squibb, Clovis Oncology, Eisai, Janssen, Lilly, MSD, Novartis, Pfizer, Roche. |
| Choi 2023 | GC Biopharma Corp |
| Gan 2018 | AbbVie |
| Lassman 2019 | AbbVie |
| Caccese 2020 | N/A |
| Reardon 2013 | GlaxoSmithKline |
| Van den Bent 2020 | AbbVie |
| Lassman 2023 | AbbVie |
| Schuster 2015 | Celldex Therapeutics, Inc |
| Sampson 2010 | NCI, National Institute of Neurological Disorders and Stroke |
| Weller 2017 | Celldex Therapeutics, Inc |
| Goff 2019 | Centre for Cancer Research |
| Bagley 2024 | Kite Pharma (a Gilead company), the Abramson Cancer Center Glioblastoma Translational Center of Excellence to D.M.O., the Templeton Family Initiative in Neuro-Oncology to D.M.O., the Maria and Gabriele Troiano Brain Cancer Immunotherapy Fund to D.M.O., National Institutes of Health grants R35NS116843 to H.S. and R35NS097370 to G.-L.M. and the Dr. Miriam and Sheldon G. Adelson Medical Research Foundation to G.-L.M |
| Gerstner 2015 | Adult Brain Tumor Consortium and an American Recovery and Reinvestment Act of 2009 (ARRA) grant |
| Batchelor 2010 | NIH |
| Batchelor 2013 | Tracy T. Batchelor, AstraZeneca, Millennium, Pfizer; Paul Mulholland, AstraZeneca; Mario Campone, Novartis; Antje Wick, Boehringer Ingelheim, Apogenix, Eli Lilly; Tom Mikkelsen, Roche, Merck, Celgene; Surasak Phuphanich, AstraZeneca, Genetech Oncology, ImmunoCellular Therapeutics, Diffusion Pharmaceutical, Med-Immune, Boehringer Ingelheim, Myrexis; John DeGroot, Astra Zeneca, Sanofi-Aventis, EMD-Serono; Lawrence Cher, Astra Zeneca, Roche; Juliane M. Jürgensmeier, AstraZeneca; Rakesh K. Jain, MedImmune, Roche, Dyax; A. Gregory Sorensen, Astra Zeneca; Martin van den Bent, Roche |
| Batchelor 2023 | National Cancer Institute, AstraZeneca |
| Hsieh 2022 | N/A |
| Duerinck 2016 | Pfizer |
| Duerinck 2018 | N/A |
| Awada 2020 | Universitair Ziekenhuis Brussel |
| Ge 2020 | Scientific Research Common Program of Beijing Municipal Commission of Education |
| Wang 2019 | N/A |
| Zhang 2018 | N/A |
| Zhang 2022 | Sun Yat-sen University Cancer Centre |
| Schiff 2015 | N/A |
| Kalpathy-Cramer 2017 | National Comprehensive Care Network |
| Baumann 2004 | N/A |
| Chang 2004 | Schering Plough, Celgene |
| Marx 2001 | N/A |
| Fine 2003 | N/A |
| Fadul 2008 | N/A |
| Riva 2007 | N/A |
| Groves 2007 | N/A |
| Alexander 2013 | NCI, CCOP, Radiation Therapy Oncology Group |
| Penas-Prados 2015 | Merck |
| Groot 2011 | N/A |
| NCT00895180 2017 (clinicaltrials.gov) | National Cancer Institute (NCI) Eli Lilly and Company |
| Nabors 2010 | National Cancer Institute |
| Brown 2016 | Astra Zeneca, NIHR/UCH |
| D'alessandris 2013 | N/A |
| D'alessandris 2019 | N/A |
| Sathornsumetee 2010 | NIH |
| Clarke 2014 | Genentech, Inc. |
| Cardona 2021 | Foundation for Clinical and Applied Cancer Research |
| Wu 2025 | N/A |
| Hasselbalch 2010 | Cophenhagen University Hospital |
| Reardon 2020 | Celldex Therapeutics, Inc |
| Phuphanich 2017 | MedImmune |
| Andrews 2021 | Albert F Stevens Foundation, sidney Kimmel Cancer Center, Imvax, Inc. |
| Affronti 2018 | Amgen |
| Wen 2011 | Amgen |
| Cloughesy 2017 | Roche |
| Lombardi 2019 | Istituto Oncologico Veneto, Bayer |
| Lombardi 2021 | Istituto Oncologico Veneto |
| Caccesse 2024 | Italian Ministry of Health Current Research |
| Tunbekici 2024 | N/A |
| Mazzarella 2022 | Associazione Italiana per la Ricerca sul Cancro |
| Lai 2024 | National Natural Science Foundation of China, Natural Science Foundation of Guangdong Province, and Sun Yat-sen University Clinical Research |
| Wang 2024 | National Natural Science Foundation of China and Jiangsu Provincial Science Young Medical Talent Project |
| Yang 2021 | National Natural Science Foundation of China |
| Guan 2022 | National Natural Science Foundation of China |
| Cao 2021 | N/A |
| She 2021 | Nature Science Youth Foundation of Hunan Province |
| Yang 2022 | N/A |
| Zhao 2024 | M5 Project Fund of Hangzhou Cancer Hospital |
| Hainsworth 2010 | N/A |
| Hassler 2014 | N/A |
| Zustovich 2013 | N/A |
| Galanis 2013 | NIH, National Cancer Institute |
| Schiff 2018 | NIH, National Cancer Institute, Fuundation for the National Institues of Health |
| Peereboom 2013 | National Cancer Institute |
| Chen 2020 | NIH |
| Dresemann 2010 | N/A |
| Reardon 2005 | N/A |
| Wen 2006 | Brain Tumor Research Trust, Will Kraft Brain Tumor Research Fund, NIH grant |
| Raymond 2008 | Novartis |
| Hassler 2014 | N/A |
| Razis 2009 | Novartis |
| Sautter 2020 | Novartis |
| Reardon 2009 | N/A |
| Kreisl 2012 | N/A |
| Dresemann 2005 | Novartis |
| Balana 2014 | Pfizer |
| Pan 2012 | National Cancer Institute |
| Hutterer 2014 | Pfizer |
| Janssen 2024 | Pfizer |
| Faye 2023 | Pfizer |
| Martinez-Garcia 2022 | Pfizer |
| Sharma 2019 | Novartis |
| Lee 2019 | Takeda Oncology |
| NCT01137604 2022  (clinicaltrials.gov) | Eisai |
| Rha 2025 | Exelixis, Inc |
| Reardon 2013 | N/A |
| Iwamoto 2010 | National Cancer Institute |
| Lee 2015 | N/A |
| McNeill 2014 | N/A |
| Kreisl 2012 | National Cancer Institute |
| Chheda 2015 | NIH, National Cancer Institute |
| Franceschi 2012 | Bristol-Myers Squibb |
| Breen 2025 | National Cancer Institute |
| Lu-Emerson 2011 | N/A |
| Lassman 2015 | Radiation Therapy oncology group , National Cancer Institute, Bristol-Myers Squibb |
| Muhic 2013 | Boehringer Ingelheim |
| Norden 2015 | N/A |
| Wen 2018 | Exelixis, Inc |
| Odia 2016 | National Cancer Institute |
| Batchelor 2017 | National Cancer Institute |

Appendix F: PRISMA Checklists

PRISMA 2020 Checklist

| **Section and Topic** | **Item #** | **Checklist item** | **Location where item is reported** |
| --- | --- | --- | --- |
| **TITLE** | | |  |
| Title | 1 | Identify the report as a systematic review. | 1 |
| **ABSTRACT** | | |  |
| Abstract | 2 | See the PRISMA 2020 for Abstracts checklist. | 2 |
| **INTRODUCTION** | | |  |
| Rationale | 3 | Describe the rationale for the review in the context of existing knowledge. | 3-5 |
| Objectives | 4 | Provide an explicit statement of the objective(s) or question(s) the review addresses. | 3-5 |
| **METHODS** | | |  |
| Eligibility criteria | 5 | Specify the inclusion and exclusion criteria for the review and how studies were grouped for the syntheses. | 6 |
| Information sources | 6 | Specify all databases, registers, websites, organisations, reference lists and other sources searched or consulted to identify studies. Specify the date when each source was last searched or consulted. | 6-7 |
| Search strategy | 7 | Present the full search strategies for all databases, registers and websites, including any filters and limits used. | 6-7, Appendix A |
| Selection process | 8 | Specify the methods used to decide whether a study met the inclusion criteria of the review, including how many reviewers screened each record and each report retrieved, whether they worked independently, and if applicable, details of automation tools used in the process. | 6-7 |
| Data collection process | 9 | Specify the methods used to collect data from reports, including how many reviewers collected data from each report, whether they worked independently, any processes for obtaining or confirming data from study investigators, and if applicable, details of automation tools used in the process. | 7 |
| Data items | 10a | List and define all outcomes for which data were sought. Specify whether all results that were compatible with each outcome domain in each study were sought (e.g. for all measures, time points, analyses), and if not, the methods used to decide which results to collect. | 7, Appendix C |
|  | 10b | List and define all other variables for which data were sought (e.g. participant and intervention characteristics, funding sources). Describe any assumptions made about any missing or unclear information. | 7, Appendix C |
| Study risk of bias assessment | 11 | Specify the methods used to assess risk of bias in the included studies, including details of the tool(s) used, how many reviewers assessed each study and whether they worked independently, and if applicable, details of automation tools used in the process. | 7 |
| Effect measures | 12 | Specify for each outcome the effect measure(s) (e.g. risk ratio, mean difference) used in the synthesis or presentation of results. | N/A |
| Synthesis methods | 13a | Describe the processes used to decide which studies were eligible for each synthesis (e.g. tabulating the study intervention characteristics and comparing against the planned groups for each synthesis (item #5)). | 7 |
|  | 13b | Describe any methods required to prepare the data for presentation or synthesis, such as handling of missing summary statistics, or data conversions. | 7 |
|  | 13c | Describe any methods used to tabulate or visually display results of individual studies and syntheses. | 7 |
|  | 13d | Describe any methods used to synthesize results and provide a rationale for the choice(s). If meta-analysis was performed, describe the model(s), method(s) to identify the presence and extent of statistical heterogeneity, and software package(s) used. | 7 |
|  | 13e | Describe any methods used to explore possible causes of heterogeneity among study results (e.g. subgroup analysis, meta-regression). | N/A |
|  | 13f | Describe any sensitivity analyses conducted to assess robustness of the synthesized results. | N/A |
| Reporting bias assessment | 14 | Describe any methods used to assess risk of bias due to missing results in a synthesis (arising from reporting biases). | 7 |
| Certainty assessment | 15 | Describe any methods used to assess certainty (or confidence) in the body of evidence for an outcome. | 7 |
| **RESULTS** | | |  |
| Study selection | 16a | Describe the results of the search and selection process, from the number of records identified in the search to the number of studies included in the review, ideally using a flow diagram. | 8, Figure 1 |
|  | 16b | Cite studies that might appear to meet the inclusion criteria, but which were excluded, and explain why they were excluded. | Figure 1, Appendix B |
| Study characteristics | 17 | Cite each included study and present its characteristics. | Supplementary Table 1 |
| Risk of bias in studies | 18 | Present assessments of risk of bias for each included study. | Appendix D |
| Results of individual studies | 19 | For all outcomes, present, for each study: (a) summary statistics for each group (where appropriate) and (b) an effect estimate and its precision (e.g. confidence/credible interval), ideally using structured tables or plots. | Supplementary Table 3, Figure 2 |
| Results of syntheses | 20a | For each synthesis, briefly summarise the characteristics and risk of bias among contributing studies. | 8-17, Supplementary Table 3 |
|  | 20b | Present results of all statistical syntheses conducted. If meta-analysis was done, present for each the summary estimate and its precision (e.g. confidence/credible interval) and measures of statistical heterogeneity. If comparing groups, describe the direction of the effect. | 8-17, Supplementary Table 3 |
|  | 20c | Present results of all investigations of possible causes of heterogeneity among study results. | N/A |
|  | 20d | Present results of all sensitivity analyses conducted to assess the robustness of the synthesized results. | N/A |
| Reporting biases | 21 | Present assessments of risk of bias due to missing results (arising from reporting biases) for each synthesis assessed. | 8-17, Appendix D |
| Certainty of evidence | 22 | Present assessments of certainty (or confidence) in the body of evidence for each outcome assessed. | 8-17,Supplementary table 1-3 |
| **DISCUSSION** | | |  |
| Discussion | 23a | Provide a general interpretation of the results in the context of other evidence. | 18-21 |
|  | 23b | Discuss any limitations of the evidence included in the review. | 18-21 |
|  | 23c | Discuss any limitations of the review processes used. | 18-21 |
|  | 23d | Discuss implications of the results for practice, policy, and future research. | 22-23 |
| **OTHER INFORMATION** | | |  |
| Registration and protocol | 24a | Provide registration information for the review, including register name and registration number, or state that the review was not registered. | N/A |
|  | 24b | Indicate where the review protocol can be accessed, or state that a protocol was not prepared. | N/A |
|  | 24c | Describe and explain any amendments to information provided at registration or in the protocol. | N/A |
| Support | 25 | Describe sources of financial or non-financial support for the review, and the role of the funders or sponsors in the review. | 25 |
| Competing interests | 26 | Declare any competing interests of review authors. | N/A |
| Availability of data, code and other materials | 27 | Report which of the following are publicly available and where they can be found: template data collection forms; data extracted from included studies; data used for all analyses; analytic code; any other materials used in the review. | Supplementary table 1-3 |

PRISMA 2020 for Abstracts Checklist

| **Section and Topic** | **Item #** | **Checklist item** | **Reported (Yes/No)** |
| --- | --- | --- | --- |
| **TITLE** | | |  |
| Title | 1 | Identify the report as a systematic review. | Yes |
| **BACKGROUND** | | |  |
| Objectives | 2 | Provide an explicit statement of the main objective(s) or question(s) the review addresses. | Yes |
| **METHODS** | | |  |
| Eligibility criteria | 3 | Specify the inclusion and exclusion criteria for the review. | Yes |
| Information sources | 4 | Specify the information sources (e.g. databases, registers) used to identify studies and the date when each was last searched. | Yes |
| Risk of bias | 5 | Specify the methods used to assess risk of bias in the included studies. | Yes |
| Synthesis of results | 6 | Specify the methods used to present and synthesise results. | Yes |
| **RESULTS** | | |  |
| Included studies | 7 | Give the total number of included studies and participants and summarise relevant characteristics of studies. | Yes |
| Synthesis of results | 8 | Present results for main outcomes, preferably indicating the number of included studies and participants for each. If meta-analysis was done, report the summary estimate and confidence/credible interval. If comparing groups, indicate the direction of the effect (i.e. which group is favoured). | Yes |
| **DISCUSSION** | | |  |
| Limitations of evidence | 9 | Provide a brief summary of the limitations of the evidence included in the review (e.g. study risk of bias, inconsistency and imprecision). | Yes |
| Interpretation | 10 | Provide a general interpretation of the results and important implications. | Yes |
| **OTHER** | | |  |
| Funding | 11 | Specify the primary source of funding for the review. | N/A |
| Registration | 12 | Provide the register name and registration number. | N/A |

**PRISMA S Checklist**

| **Section/topic** | **#** | **Checklist item** | **Location(s) Reported** |
| --- | --- | --- | --- |
| **INFORMATION SOURCES AND METHODS** | | | |
| Database name | 1 | Name each individual database searched, stating the platform for each. | 6-7 |
| Multi-database searching | 2 | If databases were searched simultaneously on a single platform, state the name of the platform, listing all of the databases searched. | N/A |
| Study registries | 3 | List any study registries searched. | 6-7 |
| Online resources and browsing | 4 | Describe any online or print source purposefully searched or browsed (e.g., tables of contents, print conference proceedings, web sites), and how this was done. | 6-7 |
| Citation searching | 5 | Indicate whether cited references or citing references were examined, and describe any methods used for locating cited/citing references (e.g., browsing reference lists, using a citation index, setting up email alerts for references citing included studies). | 6-7 |
| Contacts | 6 | Indicate whether additional studies or data were sought by contacting authors, experts, manufacturers, or others. | 6-7 |
| Other methods | 7 | Describe any additional information sources or search methods used. | 6-7 |
| **SEARCH STRATEGIES** | | | |
| Full search strategies | 8 | Include the search strategies for each database and information source, copied and pasted exactly as run. | Appendix A |
| Limits and restrictions | 9 | Specify that no limits were used, or describe any limits or restrictions applied to a search (e.g., date or time period, language, study design) and provide justification for their use. | 6-7 |
| Search filters | 10 | Indicate whether published search filters were used (as originally designed or modified), and if so, cite the filter(s) used. | 6-7 |
| Prior work | 11 | Indicate when search strategies from other literature reviews were adapted or reused for a substantive part or all of the search, citing the previous review(s). | N/A |
| Updates | 12 | Report the methods used to update the search(es) (e.g., rerunning searches, email alerts). | N/A |
| Dates of searches | 13 | For each search strategy, provide the date when the last search occurred. | 6-7 |
| **PEER REVIEW** | | | |
| Peer review | 14 | Describe any search peer review process. | 7 |
| **MANAGING RECORDS** | | | |
| Total Records | 15 | Document the total number of records identified from each database and other information sources. | Figure 1 |
| Deduplication | 16 | Describe the processes and any software used to deduplicate records from multiple database searches and other information sources. | 6-7 |
